# The Cowpea Kinome: Genomic and Transcriptomic Analysis Under Biotic and Abiotic Stresses

**DOI:** 10.3389/fpls.2021.667013

**Published:** 2021-06-14

**Authors:** José Ribamar Costa Ferreira-Neto, Artemisa Nazaré da Costa Borges, Manassés Daniel da Silva, David Anderson de Lima Morais, João Pacífico Bezerra-Neto, Guillaume Bourque, Ederson Akio Kido, Ana Maria Benko-Iseppon

**Affiliations:** ^1^Laboratory of Molecular Genetics, Genetics Department, Federal University of Pernambuco, Recife, Brazil; ^2^Laboratory of Plant Genetics and Biotechnology, Genetics Department, Federal University of Pernambuco, Recife, Brazil; ^3^Centre de Calcul Scientifique, Université de Sherbrooke, Sherbrooke, QC, Canada; ^4^Génome Québec Innovation Centre, McGill University, Montréal, QC, Canada

**Keywords:** plants, mechanical injury, virus, kinase, root dehydration

## Abstract

The present work represents a pioneering effort, being the first to analyze genomic and transcriptomic data from *Vigna unguiculata* (cowpea) kinases. We evaluated the cowpea kinome considering its genome-wide distribution and structural characteristics (at the gene and protein levels), sequence evolution, conservation among Viridiplantae species, and gene expression in three cowpea genotypes under different stress situations, including biotic (injury followed by virus inoculation—CABMV or CPSMV) and abiotic (root dehydration). The structural features of cowpea kinases (VuPKs) indicated that 1,293 *bona fide* VuPKs covered 20 groups and 118 different families. The RLK-Pelle was the largest group, with 908 members. Insights on the mechanisms of VuPK genomic expansion and conservation among Viridiplantae species indicated dispersed and tandem duplications as major forces for VuPKs’ distribution pattern and high orthology indexes and synteny with other legume species, respectively. *K*_*a*_/*K*_*s*_ ratios showed that almost all (91%) of the tandem duplication events were under purifying selection. Candidate *cis*-regulatory elements were associated with different transcription factors (TFs) in the promoter regions of the RLK-Pelle group. C2H2 TFs were closely associated with the promoter regions of almost all scrutinized families for the mentioned group. At the transcriptional level, it was suggested that VuPK up-regulation was stress, genotype, or tissue dependent (or a combination of them). The most prominent families in responding (up-regulation) to all the analyzed stresses were RLK-Pelle_DLSV and CAMK_CAMKL-CHK1. Concerning root dehydration, it was suggested that the up-regulated VuPKs are associated with ABA hormone signaling, auxin hormone transport, and potassium ion metabolism. Additionally, up-regulated VuPKs under root dehydration potentially assist in a critical physiological strategy of the studied cowpea genotype in this assay, with activation of defense mechanisms against biotic stress while responding to root dehydration. This study provides the foundation for further studies on the evolution and molecular function of VuPKs.

## Introduction

Protein kinases (PKs) comprise the largest family of “molecular switches” responsible for orchestrating protein activities. These actors are associated with almost all essential cellular functions, modifying other proteins by chemically adding phosphate groups (i.e., phosphorylation), resulting in their activation (Rauch et al., 2011). Members of the PK superfamily are involved in the signaling pathways of plants in response to both abiotic (Jaggi, 2018) and biotic (Zhu, 2016) stresses, developmental processes (McCready et al., 2020), and others, suggesting their critical participation in plant homeostasis and adaptation responses.

Despite the highlighted importance on plant molecular physiology, only a limited set of species have available information on the genomics and transcriptomics of their kinomes (i.e., the whole set of kinases), including *Arabidopsis* (Ritsema et al., 2007; Zulawski et al., 2014), tomato (Singh et al., 2013), maize (Wei et al., 2014), soybean (Liu et al., 2015), *Brassica napus* (Gill et al., 2017), pineapple (Zhu et al., 2018a), grapevine (Zhu et al., 2018b), *Gossypium* spp. (Yan et al., 2018), and wild strawberry (Liu et al., 2020). Other 25 plant species (Lehti-Shiu and Shiu, 2012) present data on their PK classification, evolutionary history, and some considerations on their molecular functions.

So far, only preliminary studies with less sensitive technologies (SuperSAGE tags) have been developed, addressing the PK gene expression in cowpea (*Vigna unguiculata*) (Kido et al., 2011). The mentioned species is a major legume commonly grown on poor soils in warm, dry areas of Africa, Asia, South America, and the United States. Its social role is important, being a relevant source of proteins and minerals for millions of people in sub-Saharan Africa and other developing regions (Boukar et al., 2016). Due to its relevance, significant efforts in the omics field have been carried out for cowpea. Recently, its reference genome was published (Lonardi et al., 2019). The mentioned structure has been fundamental for understanding the physiological superiority of this crop under stresses, for mining of genes with biotechnological potential, and for comparative legume genomics studies.

In Brazil, country responsible for 12% of the world’s cowpea production (Boukar et al., 2016), the Cowpea Genomics Consortium (CpGC) was established. This biotechnological network sequenced genomes from different genotypes, which are in an active process of assembly. Besides this, the CpGC has assembled transcriptomes (RNA-Seq) for tolerant/resistant cultivars under root dehydration (a critical component of the complex effects of drought stress) and under combined stresses [mechanical injury of leaves followed by viral inoculation of cowpea aphid-borne mosaic virus (CABMV) or cowpea severe mosaic virus (CPSMV)—two important pathogenic viruses of the mentioned crop]. The available transcriptomic data at CpGC and the diversity of treatments and tissues studied, added to the public data of the cowpea reference genome, represent a rich source of biological information.

The present study aimed to explore and characterize the cowpea’s kinome. For this purpose, 1,293 loci encoding cowpea kinases (VuPKs) were identified in the reference genome and categorized into groups and families. Additionally, VuPK genes were structurally characterized; their products were analyzed considering subcellular location and protein properties. Further approaches included the study of VuPK expansion mechanisms in cowpea genome; the determination of associated *cis*-regulatory elements in VuPK promoter regions as well as VuPK gene orthology within Viridiplantae and synteny analysis among legumes of socioeconomic importance also were carried out. Finally, the CpGC transcriptomics data were scrutinized for VuPK identification and gene expression studies. The present work provides significant advances in the studied theme and brings a starting point for further experimental research in cowpea molecular biology.

## Materials and Methods

### Genomics Approach

#### Kinase Mining and Identification

The VuPK identification and classification were computationally based on two steps: first, using the iTAK (Zheng et al., 2016) software. This tool has the potential to identify up to 150 kinase families by performing two main actions:

(a) Kinase identification: if the analyzed sequences have a significant hit (at least 50% of the Pfam domain model) to the protein kinase domains (PF00069, PF07714, or PF00481) in the Pfam database.
(b) Kinase classification: after step “a”, the identified VuPKs are classified into gene families by comparing their sequences to a set of Hidden Markov Models (*e*-value cutoff < 1.0e^–5^) developed by Lehti-Shiu and Shiu (2012).

The second strategy was based on a distance analysis using sequences with kinase domain to confirm the iTAK result. For this purpose, a multiple alignment of candidate VuPKs was generated using the ClustalW (Larkin et al., 2007) and a tree, obtained by neighbor-joining (NJ) method (Saitou and Nei, 1987) with a bootstrap of 1,000 replicates (default parameters).

The annotation of a VuPK was considered accurate (*bona fide* VuPK) when members of the same family, according to the result of the iTAK software, grouped with their peers in the obtained NJ tree. If the iTAK results and the NJ tree were not convergent, the divergent element was indicated as “unknown” (UNK).

Considering the reference genome, the mentioned steps were carried out against the predicted cowpea proteome (Vunguiculata_469_v1.1.protein_primaryTranscriptOnly.fa; Phytozome database V12). Only the longest peptide sequence for each cowpea gene was used. The same steps were carried out to identify *bona fide* VuPKs in the cowpea RNA-Seq libraries at CpGC. For gene expression studies, all identified VuPK isoforms were scrutinized.

#### VuPKs Genes: Features and Expansion Mechanisms

The VuPK gene features were analyzed *via* Genestats script^1^. The following parameters were scrutinized: (1) transcript sequence length, (2) number of exons, (3) total exon sequence length, (4) number of introns, (5) total intron sequence length, (6) number of CDS chunks, (7) total CDS sequence length, (8) number of 5’ UTR sequences, (9) total 5′ UTR sequence length, (10) number of 3′ UTR sequences, and (11) total 3′ UTR sequence length. The number of kinase domains (12) was annotated according to the iTAK output.

Multiple Collinearity Scan toolkit (MCScanX; Wang et al., 2012) package—downstream analysis mode: “duplicate_gene_classifier”—was applied to classify the origins of the duplicated VuPK genes. The following procedure was used by MCScanX to assign the duplication mechanisms: (1) All genes were initially classified as “singletons” (i.e., no duplicates within the cowpea genome) and assigned gene ranks according to their order of appearance along chromosomes; (2) BLASTp results were evaluated, and the genes with BLASTP hits to other genes were re-labeled as “dispersed duplicates”; (3) in any BLASTp hit, two genes were re-labeled as “proximal duplicates” if they had a difference of gene rank <20; (4) in any BLASTp hit, two genes were re-labeled as “tandem duplicates” if they had a difference of gene rank = 1; (5) MCScanX was executed. The anchor genes in collinear blocks were re-labeled as “WGD/segmental”; (6) so, if a gene appeared in multiple BLASTp hits, it was assigned a unique class according to the order of priority: whole-genome/segmental > tandem > proximal > dispersed.

Synteny analyses between “cowpea *versus* soybean” and “cowpea *versus* common bean” were carried out using the same software (downstream analysis mode: “dual_synteny_plotter”) and using default parameters.

#### Ratio of Synonymous and Non-synonymous Substitutions per Site for Tandem Duplicated Genes

The ratio of non-synonymous (*K*_*a*_) to synonymous (*K*_*s*_) substitutions (*K*_*a*_/*K*_*s*_) was used to determine the selection pressure among tandem duplication events. ClustalW 2.0 (Larkin et al., 2007) software first aligned the full-length coding sequences of tandemly duplicated VuPK genes. Then, the *K*_*a*_ and *K*_*s*_ rates were calculated with the standard genetic code table by the Nei–Gojobori method (Jukes–Cantor model) implemented on MEGA 7 (Kumar et al., 2016).

The *K*_*a*_/*K*_*s*_ ratio is an indicator of the selection history on genes or gene regions: (i) if its value is lower than “1,” the duplicated gene pairs may have evolved from purifying selection (also called negative selection; conserves the amino acid sequence), (ii) if *K*_*a*_/*K*_*s*_ equals “1,” which means neutral selection (that had no constraint for sequence divergence); and (iii) when *K*_*a*_/*K*_*s*_ is greater than “1,” it means positive selection (that led to different peptides).

#### Orthology of VuPKs

For orthology detection, information available in the Phytozome^2^ was retrieved. In the section PhytoMine (an InterMine interface to data from Phytozome), a query was created in the form of a list with the identities of the genes to be analyzed. Ninety-three assembled and annotated genomes from 82 Viridiplantae species were searched.

The result was generated by InParanoid (Sonnhammer and Östlund, 2015). This software uses pairwise similarity scores, calculated using BLASTp between two complete proteomes, for constructing orthology groups using the similarity criteria described by Ostlund et al. (2010).

#### Candidate *Cis*-Regulatory Element Enrichment Analysis

Sequences of promoter regions (within 1.0-kb range) of VuPK-encoding genes were obtained from the Phytozome v.12^2^ database by Application Programming Interface. The motifs (candidate *cis*-regulatory elements, CCREs) in each promoter region were searched by the software MEME, v5.0.3^3^ (Bailey and Elkan, 1994). For each identified motif, the software reports a corresponding *e*-value. In the present work, an *e*-value < 10^–2^ was adopted as a cutoff point for the characterization of enriched *bona fide* CCREs. The maximum number of motifs analyzed in the present study for a single VuPK promoter region was 10, and the extension ranged from six to 50 nt.

The TomTom software, v4.11.2^4^ (Gupta et al., 2007), was used coupled to the JASPAR (file: JASPAR2018_CORE_plants_non-redundant) database, aiming to assign a transcription factor (TF) to the *bona fide* CCREs. Such software aligns the enriched *bona fide* CCREs identified by MEME against a database (JASPAR) of annotated motifs. These alignments were assessed using the following statistical criteria: *p*-value (cutoff < 10^–2^) and *q*-value [false discovery rate (FDR) cutoff < 10^–2^]. In this work, the identities of TFs associated with the enriched *bona fide* CCREs were related to the best hit obtained.

#### Protein Properties and Subcellular Localization Prediction

The molecular weight (MW) and isoelectric point (pI) for all of the VuPK proteins encoded in the cowpea reference genome (Lonardi et al., 2019) were predicted using the JVir Gel online tool (Hiller et al., 2006). The predicted subcellular localizations of the mentioned VuPKs were determined using the CELLO tool (Yu et al., 2006).

### Transcriptomics Approach

#### Biological Material, Experimental Design, and Stress Application

##### Root dehydration assay

Seeds of *V. unguiculata* cv. Pingo de Ouro (considered tolerant to water deficit and drought; Bastos et al., 2011; Rodrigues et al., 2017) were treated with 0.05% (w/v) Thiram (tetramethylthiuram disulfide) and germinated during 2 days at 25 ± 1°C and 65 ± 5% of temperature and relative humidity, respectively. The seedlings were transferred to a hydroponic system (Rodrigues et al., 2012) (Supplementary Figure 1A) with aerated pH 6.6 balanced nutrient solution (Hoagland and Arnon, 1950). Plantlets were placed in supports so that the roots of the seedlings were wholly immersed in the solution (Supplementary Figure 1A). The plantlets were grown for 3 weeks (developmental stage V3) in a greenhouse under a natural photoperiod of approximately 13/11 h light/dark cycle, temperature of 30 ± 5°C and 60 ± 10% relative humidity. After this period, root dehydration treatment was initiated by withdrawing the nutrient solution from treated plants (Supplementary Figure 1A). The roots were collected after 25 min (RD25) and 150 min (RD150) after solution removal (Supplementary Figure 1B). The tissue was immediately frozen in liquid nitrogen and stored at −80°C until RNA extraction. For each treatment, the respective control plants (Cont.25′ and Cont.150′; Supplementary Figure 1B) were maintained in the nutrient solution and subsequently collected. The experimental design was factorial (cultivar vs. extension of root dehydration period) with three biological replicates (RBs) for each control and treatment implemented (Supplementary Figure 1B). Each RB was composed of two individuals.

Throughout this work, the gene expression contrast for ‘RD25 vs. Cont.25’ was indicated as T25, similar to ‘RD150 vs. Cont.150’, indicated as T150.

The seeds of the cowpea ‘Pingo de Ouro’ genotype (ID: Pingo_de_Ouro_ 1_2) were formally obtained from the ‘Cowpea Germplasm Active Bank’ of the Agronomic Institute of Pernambuco (AIP; Recife, Pernambuco, Brazil). Prof. Dr. Antônio Félix da Costa (AIP) kindly carried out the species/genotype identification.

##### Mechanical injury and virus inoculation assays

The mechanical injury and inoculation with CABMV or CPSMV experiments were carried out under controlled conditions in a greenhouse at the Instituto Agronômico de Pernambuco (IPA; Recife, Pernambuco, Brazil) (Supplementary Figure 2A). For the CABMV assay, the resistant genotype IT85F-2687 (Rocha et al., 1996; Oliveira et al., 2012) was used, whereas for CPSMV, the resistant genotype BR-14 Mulato (Cardoso et al., 1990) was analyzed.

The experimental procedure for both assays was conducted separately and in the same way. Both genotypes were sown and cultivated for 3 weeks (V3 developmental stage) under natural photoperiod and temperature ranging from 28 to 32°C (Supplementary Figure 2A). Young trifoliolate leaves were mechanically injured with carborundum (silicon carbide) to allow virus penetration. After that, the viral inoculum was applied (Supplementary Figure 2A).

Two collection times were performed after the injury/inoculation for each assay (Supplementary Figure 2B): 60 min and 16 h. Each treatment had its respective absolute or mock control (Supplementary Figure 2B). Tissues were immediately frozen in liquid nitrogen and stored at −80°C until RNA extraction.

The experimental design was factorial (cultivar *versus* extension of the post-inoculation period) with three RBs for each control and treatment implemented (Supplementary Figure 2B). Each of these RBs was composed of five plants. Each treatment was carried out in an isolated area to avoid the impact of volatile compounds used by the plants for communication.

The seeds of the cowpea’s ‘IT85F-2687’ and ‘BR-14 Mulato’ genotypes were formally obtained from the ‘Cowpea Germplasm Active Bank’ of the Agronomic Institute of Pernambuco (IPA; Recife, Pernambuco, Brazil). Prof. Dr. Antônio Félix da Costa (AIP) kindly carried out the species/genotype identification.

The differential gene expression of the two tests involving viruses was composed of a combination of two stresses: mechanical injury and viral inoculation. Plant viruses are unable to initiate an infectious process without assistance (from a vector organism or due to certain agricultural practices, for example) since such organisms are incapable of penetrating the plant cell wall. According to Barna and Király (2004), plant viruses do not have specific cell receptors, unlike bacteriophages and viruses that infect animals. Thus, the set ‘mechanical injury and viral inoculation’ aims to mimic the infectious process in a natural environment.

#### RNA-Seq Libraries: Synthesis and Sequencing

Total RNA was isolated using the ‘SV Total RNA Isolation System’ kit (Promega, United States) following the manufacturer’s protocol. Agarose gel (1.5%) and Agilent 2100 Bioanalyzer (Agilent Technologies, EUA) were used to evaluate both the concentration and the quality of total extracted RNA. Only samples with RNA integrity number ≥ 8.0 were sequenced. A ‘RNAm TruSeq^®^ Stranded LT-Set A’ kit (RS-122-2101) (Illumina, San Diego, CA, United States) was employed in messenger RNA purification and cDNA library construction according to the manufacturer’s instructions. Paired-end reads, with 100 pb in length, were generated *via* the Illumina HiSeq^®^ 2500 system using the following kits: ‘HiSeq Rapid PE Cluster Kit v2’ (PE-402-4002), ‘SBS Kit v2’ (200 Cycle; FC-402-4021), and ‘TruSeq^®^ Stranded mRNA LT—Set A’ (RS-122-2101). All sequencing steps were performed at the Center for Functional Genomics, University of São Paulo (Piracicaba, Brazil).

#### RNA-Seq Libraries Assembly and Differential Expression Analysis

The 12 RNA-Seq libraries sequenced for the root dehydration assay were assembled together with the 12 from the duet ‘mechanical injury and viral inoculation by CABMV,’ in addition to the other 12 from a combination of ‘mechanical injury and inoculation by CPSMV.’ Such joint assembly allowed to obtain longer and more robust transcripts. Seventy-two replicates (12 biological for root dehydration + 12 biological for CABMV + 12 biological for CPSMV, with two technical replicates each) were submitted to the pipeline.

Raw reads were assembled with the GenPipes project (Bourgey et al., 2019)—‘RNA-Seq Pipeline’—of the McGill University and Génome Québec Innovation Center (C3G). All differential gene expression analyses were performed independently for each assay (root dehydration, mechanical injury + CABMV inoculation, mechanical injury + CPSMV inoculation). Such action was performed by the edgeR tool (Robinson et al., 2010), implemented in the mentioned GenPipes pipeline. Transcripts with −1 > log2FC > 1, *p* < 0.05, and FDR < 0.05 were considered differentially expressed.

#### Gene Ontology Enrichment Analysis

For the proposed analysis, we used the Network-Based Visualization for Omics (NeVOmics) tool (Zúñiga-León et al., 2018) that identifies statistically over-represented biological process Gene Ontology (GO) terms within a given gene/protein set. NeVOmics adopts GeneMerge statistical algorithm to obtain the over-represented functions or categories in the input list. A hypergeometric distribution (*p* < 0.05) and an FDR correction (FDR < 0.05) were applied to identify the statistically more represented function annotations. For each treatment studied, the whole ‘control *vs*. treatments’ transcriptomes were used as background in the NeVOmics enrichment analyses.

NeVOmics uses an input file in plain text containing a list of genes (KEGG gene ID) or proteins (UniProt Entry ID) obtained by the omics approach. Due to the reduced number (48,364, unreviewed—April| 2020) of proteins annotated for *V. unguiculata* at the UniProt^5^ database, the annotation was performed against *Arabidopsis thaliana*. This plant species has a much more abundant data amount (163877, unreviewed—April| 2020) at UniProt, besides presenting the best information index (16181, manually annotated—April| 2020) among all plants in the database.

To retrieve the *A. thaliana* UniProt IDs, initially, we obtained proteins from transcriptomes of the CpGC database translated *via* TransDecoder^6^, a utility included in Trinity software. Then, a BLASTp was performed (cutoff < e^–10^) between the proteomes of *A. thaliana* (at UniProt) and cowpea (from CpGC). Only the best hit for each alignment was sent for further analysis.

#### qPCR: Setup, cDNA Synthesis, Efficiency Analysis, and Relative Expression

These activities were performed according to the MIQE guidelines (Bustin et al., 2009). To evaluate the transcriptomic data’s accuracy, 10 VuPK transcripts (Supplementary Table 1), with up-regulation indicated in RNA-Seq libraries, were randomly selected for qPCR investigation. Three biological and three technical replicates were used per analyzed treatment, aiming to guarantee the statistical reliability of the process. qPCR reactions were performed in 96-well plates at LineGene 9660 (Bioer) using the SYBR Green detection method.

Aliquots of the same total RNA sample sent for sequencing of the RNA-Seq libraries were employed in this step. Possible contamination with genomic DNA (gDNA) was eliminated *via* digestion with ‘RNase-free DNase I.’ RNA quantity and quality were evaluated, respectively, using the NanoDrop ND-1000 UV–vis spectrophotometer (Thermo Fisher Scientific) and agarose gel electrophoresis 1% (p/v) stained with Green Blue (LGC, São Paulo, Brazil). For each sample studied, total RNA (1 μg) was reverse-transcribed, generating cDNA, using the “Improm-IITM Reverse Transcriptional System” kit (Promega) with oligo primers (dT) and following the manufacturer’s recommendations. qPCR setup, PCR cycling, amplification efficiency assay, primer pair design, and melting curve analysis (Supplementary Appendix 1) were performed according to Amorim et al. (2018).

“Actin” and “ubiquitin-conjugating enzyme E2 variant 1D” (Supplementary Table 1) were selected as reference genes for qPCR root dehydration assay data normalization. These were indicated from tests performed by Amorim et al. (2018) for the same cultivar, tissue, and condition. “F-box protein” and “Polyubiquitin 10” (Supplementary Table 1) were selected as reference genes for qPCR mechanical injury and viral inoculation data normalization. These were indicated from tests performed by CpGC group (data not published) for the same cultivars, tissues, and conditions.

The Rest2009 software (standard mode) was used for relative expression analysis of the target transcripts. Such analysis is based on paired comparisons (of target transcript and reference genes under stress conditions and controls) using randomization and bootstrapping—Pair-wise Fixed Reallocation Randomization Test©; Pfaffl et al., 2002). Hypothesis testing (*p* < 0.05) was used to determine if differences in the expression of target transcripts under control and treated conditions were significant.

## Results

### Genomics of VuPKs

#### Genome-Wide Identification and Classification

**FIGURE 1:**
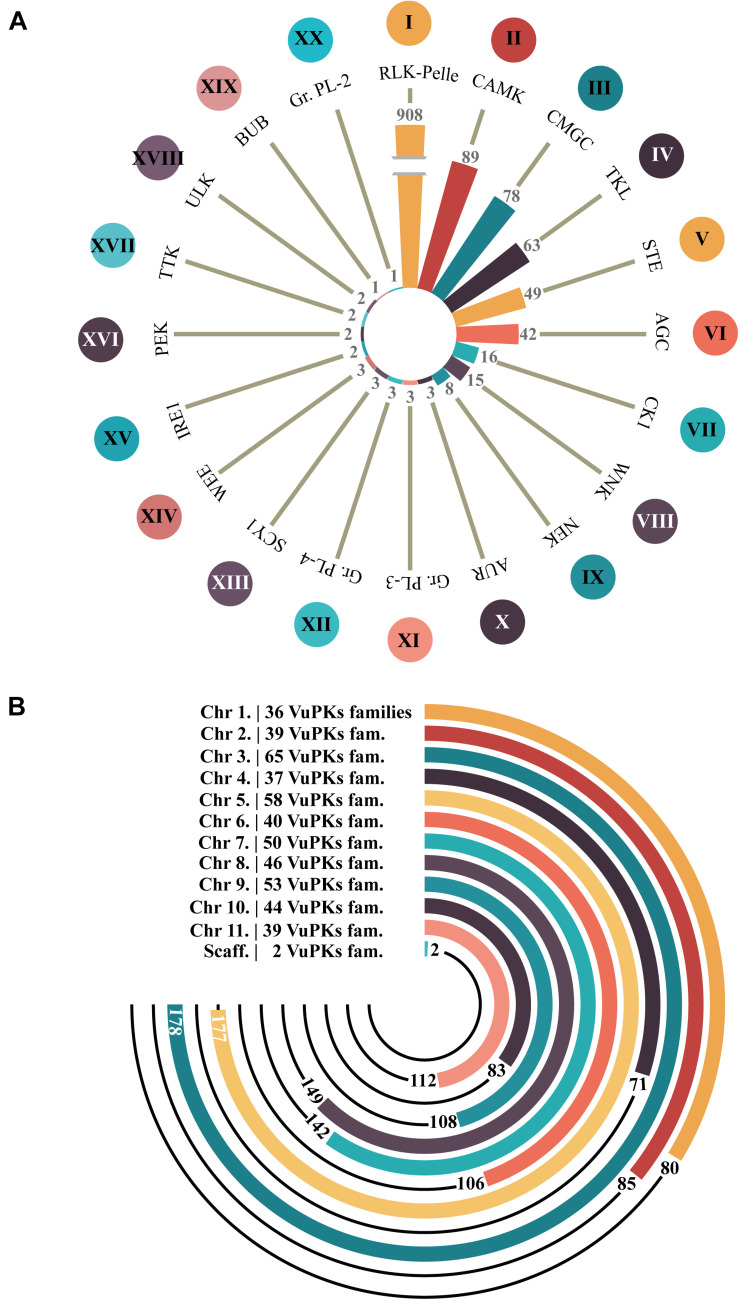
Amount of *bona fide* VuPKs in the cowpea genome. **(A)** Twenty searched groups of VuPKs and their most abundant families (indicated by Roman numerals). **(B)** Chromosomal distribution of *bona fide* VuPK loci and respective number of families covered per chromosome (family and respective number of representatives): I, RLK-Pelle_DLSV (174); II, CAMK_CDPK (40); III, CMGC_CDK-CRK7-CDK9 (19); IV, TKL_CTR1-DRK-2 (14); V, STE_STE11 (33); VI, AGC_RSK-2 (24); VII, CK1_CK1 (11); VIII, WNK_NRBP (15); IX, NEK (8); X, AUR (3), XI, Group-Pl-3 (3); XII, Group-Pl-4 (3); XIII, SCY1_SCYL1 (2); XIV, WEE (1); XV, IRE1 (2); XVI, PEK_GCN2 (1) and PEK_PEK (1); XVII, TTK (2); XVIII, ULK_ULK4 (1) and ULK_Fused (1); XIX, BUB (1); and XX, Group-Pl-2 (1).

After analyzing the iTAK software, 1,338 candidate VuPKs were identified in cowpea genome (Supplementary Table 2). This result was also contrasted by a tree-like network presenting phenetic VuPK relationships (Supplementary Figure 3). A total of 45 candidate VuPKs presented divergences between the iTAK categorization and the obtained phenetic grouping, being classified in the present work as ‘unknown’ (UNK; Supplementary Table 3) and removed from further analysis. The remaining 1,293 *bona fide* VUPKs covered 20 different groups (Figure 1A). Concerning the most abundant, RLK-Pelle (908 loci; ∼70% of the whole kinome) stands out in the cowpea genome. CAMK (89), CMGC (78), TKL (63), STE (49), and AGC (42) come next (Figure 1A).

The 20 groups covered 118 different families (Supplementary Table 4) out of 150 potentially identifiable by the iTAK tool. The most abundant families are represented by Roman numerals in Figure 1A, respectively: I—RLK-Pelle_DLSV (174), II—CAMK_CDPK (40), III—CMGC_CDK-CRK7-CDK9 (19), IV—TKL_CTR1-DRK-2 (14), V—STE_STE11 (33), and VI—AGC_RSK-2 (24) for the six groups mentioned in the paragraph above. In another quantitative context, 20 families were found to be codified by only one locus (Supplementary Table 4). Eighty-two families were encoded by less than 12 loci; only 15 families had more than 24 coding loci (Supplementary Table 4).

Concerning genomic distribution, the 1,293 *bona fide* VuPKs were anchored on all 11 cowpea chromosomes, distributed unevenly. Only two loci (one for each family, AGC-Pl and RLK-Pelle_SD-2b) were located in scaffolds (Figure 1B). Chromosome ‘3’ (Vigun03g) had the largest number of anchored loci, 178 (Figure 1B), associated with 65 different VuPK families (Figure 1B).

#### Gene Structure and Protein Property Characterization

For the study of the VuPK genes’ structural features, 12 parameters (Supplementary Tables 4, 5) were analyzed. This section will cover the number of kinase domains and the number of introns, considered biologically more informative. The average number of each feature for each family as well as the individual data for each VuPK gene are available at Supplementary Tables 4, 5, respectively.

Considering the cowpea kinase families, the average number of kinase domains per protein varied from 1.0 (RLK-Pelle_Singleton, PEK_PEK, BUB, CMGC_CDKL-Cr—about 3.0% of the VuPKs families) to 3.8 (RLK-Pelle_RLCK-XI, AGC_RSK-2—about 2.0% of the VuPK families). About 95% of the different VuPK families have, on average, between 1.9 and 3.6 kinase domains (Supplementary Table 4). Concerning the number of introns, 176 (∼13.5%) VuPK genes, associated with 26 different families, did not show this type of sequence. The remaining (1,117 or ∼86.5%) has one or more introns in its genomic structure. The average number of introns per family varied from zero (RLK-Pelle_LRR-VII-3) to 28 (PEK_GCN2) (Supplementary Table 3). Eleven (∼9.0%) out of 118 searched families had an average number of introns between 0 and 0.9 (Supplementary Table 4), indicating that most of its members do not have introns (Supplementary Table 5). The mentioned 11 families belong to RLK-Pelle group (Supplementary Table 4). Additionally, this group presented a representative (RLK-Pelle_LRR-XIIIb family; average number of introns, 20) among the seven families with an average number of introns ≥ 20 (Supplementary Table 4), demonstrating its structural heterogeneity.

The analysis of intra-family characteristics (for families with more than 10 constituent members) showed that the number of kinase domains presents few variations within each family. However, the number of introns is inconstant and can vary by up to an order of magnitude (ex.: RLK-Pelle_DLSV; Supplementary Table 5). A similar fact can be observed at the group level (ex., TKL, RLK-Pelle, CMGC, CAMK, among others; Supplementary Table 4): almost total conservation of the average number of domains among the families, with subsequent variation in the number of introns.

Subcellular localization determines the environments in which proteins operate. Such parameter influences protein function by controlling access to and the availability of all types of molecular interaction partners. A total of 1,291 *bona fide* VuPKs were divided into seven cellular compartments (Supplementary Table 6): vacuole (five VuPKs), chloroplast (129), mitochondria (150), cytoplasm (162), extracellular compartment (168), nucleus (252), and plasma membrane (425). The plasma membrane prevailed with about 96% of the *bona fide* VuPKs of the RLK-Pelle group (Supplementary Table 6). The other compartments showed a heterogeneous constitution of VuPKs. The CELLO tool did not assign subcellular location only to two VuPKs.

The pIs of the *bona fide* VuPKs varied from 4.10 to 10.00, with MWs ranging from 19,296.34 to 184,869.01 Da (Supplementary Table 6). VuPKs presented highly variable pIs and MWs in intra-family terms (Supplementary Table 6).

#### The Landscape of the Gene Duplications of the Cowpea Kinome

**FIGURE 2:**
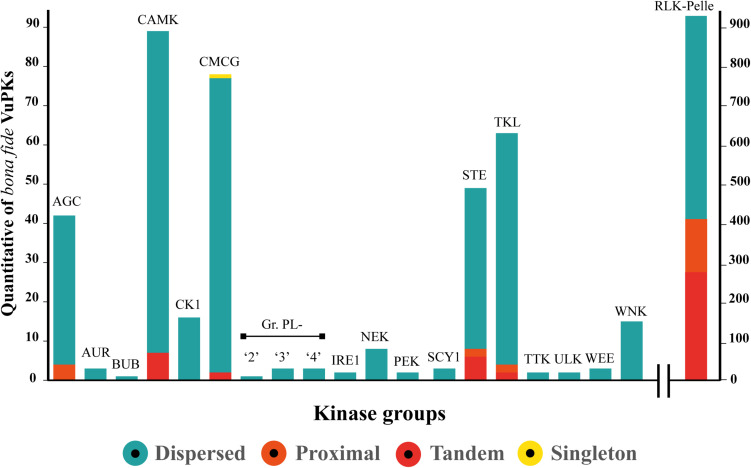
Categories and quantification (represented by the range covered by each color on the axis “*y*” scale) of the main expansion mechanisms of the 20 *bona fide* VuPK groups searched in the cowpea genome.

Gene duplication has long been regarded as an important evolutionary force that provides abundant raw materials for genetic novelty and can occur by several mechanisms. Our data indicate that no VuPK groups have been expanded from the whole genome duplication mechanism. Only one VuPK, belonging to the CMCG group, was considered singleton. A total of 140 VuPKs showed proximal duplication, with 132 (94.3%) associated with the RLK-Pelle group (Figure 2).

Furthermore, 116 VuPK tandem duplication events were found in the cowpea genome, covering a total of 309 genes, composing 190 duplicated gene pairs (Supplementary Table 7). These gene pairs belonged to five distinct groups (Figure 2): CAMK, CMCG, RLK-Pelle, STE, and TKL. Tandem duplication events with the highest number of duplicated genes occurred within the RLK-Pelle group (Supplementary Table 7).

Finally, the dispersed duplication mechanism was indicated as the main apparatus for the VuPK expansion in the cowpea genome, being the most abundant for the 20 analyzed groups (Figure 2). A total of 843 VuPK genes were duplicated by such a mechanism. Additionally, it is the only procedure responsible for expanding 13 of the 20 VuPK groups (Figure 2).

#### Diagnosing the Form of Sequence Evolution on VuPK Tandem Duplicated Genes

The forces that drive natural selection can be inferred from the types of nucleotide substitutions in the coding sequence of genes. An informative parameter of the gene evolution under selection has been the ratio of the rate of non-synonymous substitution (*K*_*a*_—that causes an amino acid change) to the rate of synonymous substitution (*K*_*s*_—that does not cause an amino acid change). After pair-to-pair comparison, the *K*_*s*_/*K*_*a*_ values varied between 0.09 and 4.67 (Supplementary Table 8) and the *K*_*s*_/*K*_*a*_ means of the 190 pairs of duplicated tandem genes was 0.60 (Supplementary Table 8). Ninety-one percent (173) of these gene pairs had *K*_*s*_/*K*_*a*_ < 1, suggesting that these genes are under purifying selection. In another context, it is observed that 9% (17) presented *K*_*s*_/*K*_*a*_ > 1, implying that positive selection played an important role in the evolution of this reduced amount of VuPK gene pairs.

#### VuPK Comparative Genomics

**FIGURE 3:**
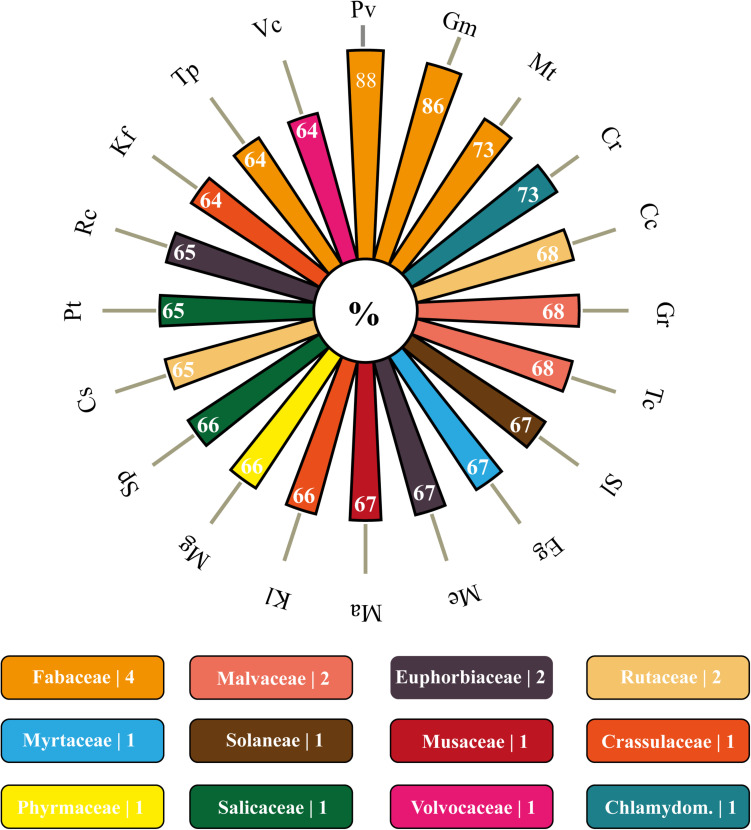
Polar bar chart with 20 species with higher orthology indexes to the *bona fide* VuPKs identified in the cowpea genome. In rectangles below, are the families covered by these 20 species as well as the number of representatives on the graph above. Corresponding colors between items on the polar bar chart and rectangles perform the association species–family. Pv, *Phaseolus vulgaris*; Gm, *Glycine max*; Mt, *Medicago truncatula*; Cr, *Capsella rubella*; Cc, *Citrus clementina*; Gr, *Gossypium raimondii*; Tc, *Theobroma cacao*; Sl, *Solanum lycopersicum*; Eg, *Eucalyptus grandis*; Me, *Manihot esculenta*; Ma, *Musa acuminata*; Kl, *Kalanchoe laxiflora*; Mg, *Mimulus guttatus*; Sp, *Salix purpurea*; Cs, *Citrus sinensis*; Pt, *Populus trichocarpa*; Rc, *Ricinus communis*; Kf, *Kalanchoe fedtschenkoi*; Tp, *Trifolium pratense*; Vc, *Volvox carteri*.

Concerning the orthology, 64 of the 93 genomes of Viridiplantae allocated in the Phytozome database presented orthologous members to the analyzed VuPKs (Supplementary Table 9). Among the searched genomes, the species *Marchantia polymorpha* (Marchantiaceae, Bryophyta) had the lowest orthology index (∼30%; 383/1,293) (Supplementary Table 9). Among the 20 species with the highest rates, the Leguminosae family stood out, presenting three members [*Phaseolus vulgaris* (88%), *Glycine max* (86%), and *Medicago truncatula* (73%); Figure 3], followed by Malvaceae [*Gossypium raimondii* (68%) and *Theobroma cacao* (68%)], Euphorbiaceae [*Manihot esculenta* (67%) and *Ricinus communis* (65%)], and Rutaceae [*Citrus clementina* (68%) and *Citrus sinensis* (65%)] (Figure 3).

**FIGURE 4:**
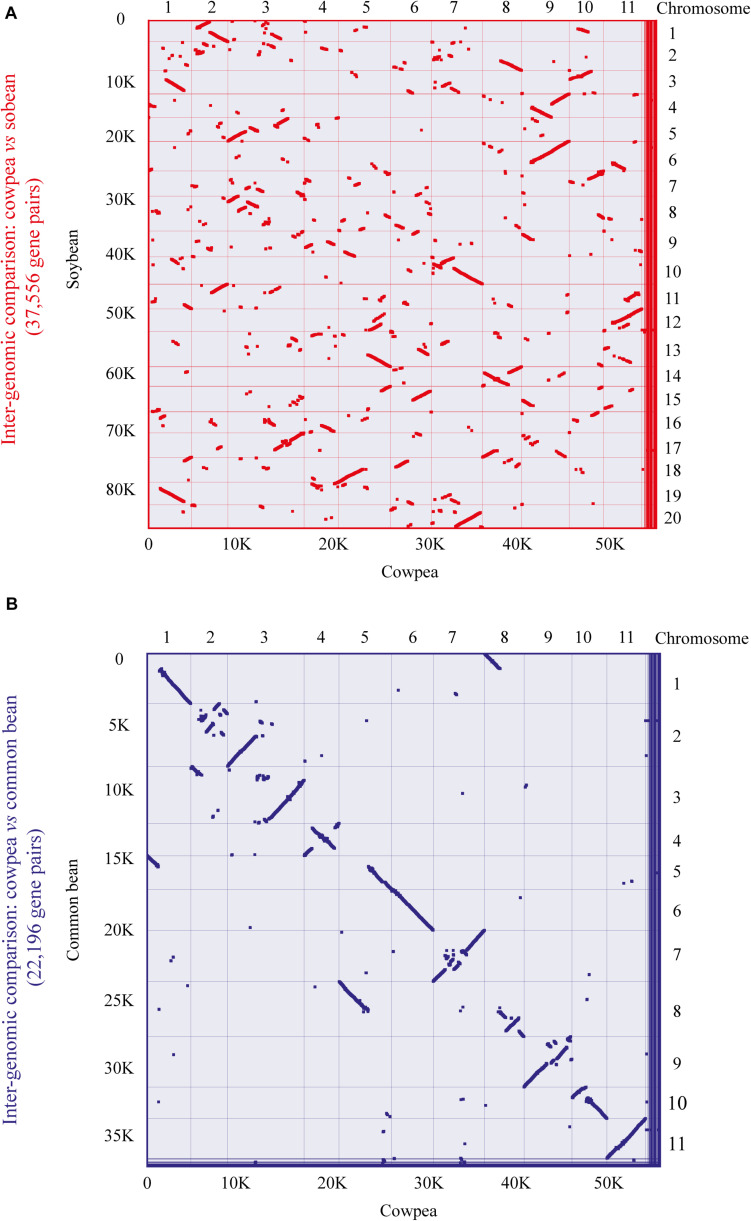
Comparative genomics between cowpea and two other crop legumes. **(A)** Macrosyntenic dot plot of the cowpea and soybean chromosomes. **(B)** Macrosyntenic dot plot of the cowpea and common bean chromosomes. Each red or blue dot represents a syntenic region (comprising at least five genes) between the two respective genomes.

**FIGURE 5:**
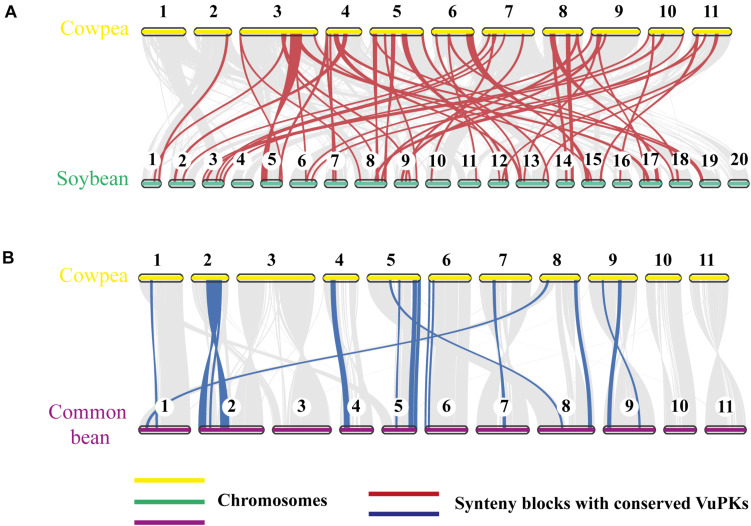
Comparative genomics between **(A)** cowpea *vs.* soybean and **(B)** cowpea *vs.* common bean. The red and blue lines connect syntenic blocks with *bona fide* VuPK-encoding loci. The gray wedges in the background highlight major syntenic blocks spanning at least five genes between the genomes.

To further analyze the aforementioned conservation, a more specific study was carried out by contrasting the cowpea genome against the genomes of the two legume species with higher orthology indexes, aiming at mining kinase-anchoring syntenic blocks. The inter-genomic studies between cowpea and soybean indicated 564 blocks of syntenic genes (Figure 4A). Of these syntenic blocks, 55 had VuPK-encoding loci (Figure 5A). For the ‘cowpea *versus* common beans’ comparison, 192 synthetic blocks were observed (Figure 4B), a lower number than the previous comparison. However, such blocks showed greater length (Figure 5B). Of these, 17 anchored VuPK-encoding loci (Figure 5B).

#### Enriched CCREs Mining in VuPK Gene Promoters: A Focus on RLK-Pelle Group Families

**FIGURE 6:**
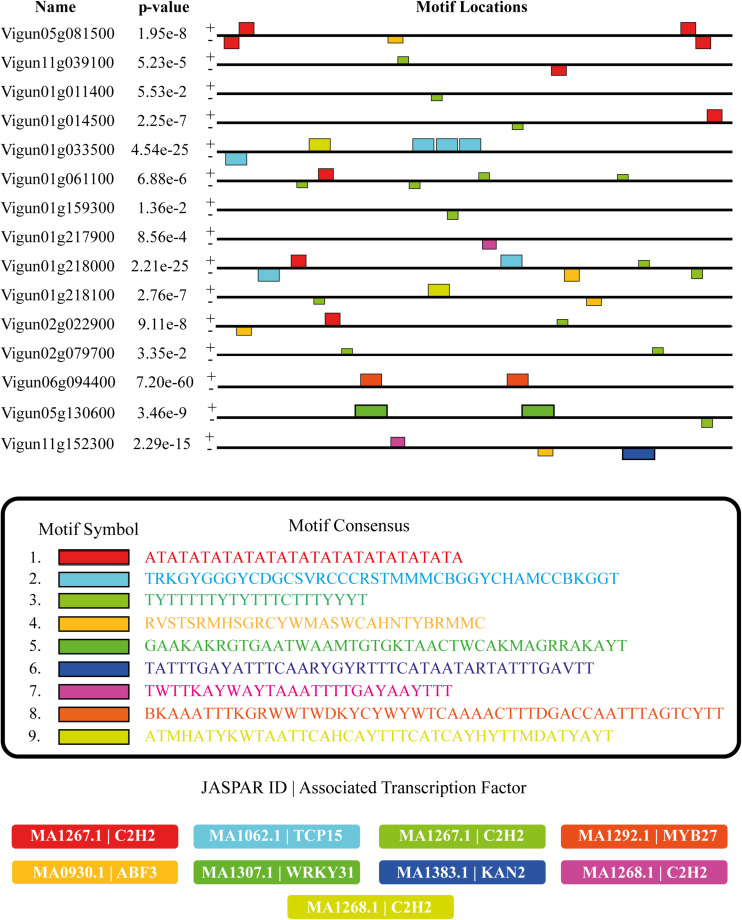
Distribution (colored rectangles) of enriched *bona fide* CCREs anchored in the promoter regions (black lines) of a sample (the plotted gene sample was deliberately chosen to present all the enriched *bona fide* CCRE types found in the complete set of promoters analyzed) of genes encoding RLK-Pelle_DLSV *bona fide* VuPKs as well as information on their motif consensus and associated transcription factors (Matrix JASPAR ID). CCREs, candidate *cis*-regulatory elements. Plus (+) and minus (–) signs indicate sense and antisense DNA strands of the analyzed promoter regions.

The following factors justify the focus on the RLK-Pelle group:

• Comprised the largest and most diverse group of VuPKs searched in the cowpea genome.
• 90% of the 10 most abundant VuPK families belong to this group.
• RLK-Pelle group presented the only families (nine in total) with *bona fide* CCREs enrichment in their promoters: RLK-Pelle_DLSV, RLK-Pelle_LRR-XI-1, RLK-Pelle_LRK10L-2, RLK-Pelle_LRR-III, RLK-Pelle_L-LEC, RLK-Pelle_RLCK-VIIa-2, RLK-Pelle_SD-2b, RLK-Pelle_CrRLK1L-1, and RLK-Pelle_LRR-XII-1 (Figure 6 and Supplementary Table 10).

Identified enriched CCREs are presented in Figure 6 (RLK-Pelle_DLSV family) and Supplementary Table 10 (other analyzed families). In the set of promoters searched, 62 enriched *bona fide* CCREs could be associated with 49 different experimentally defined transcription factor binding sites for plants (Matrix Jaspar ID). The amount varied from three (RLK-Pelle_LRR-III family) to 10 enriched *bona fide* CCREs (RLK-Pelle_LRR-XI-1 and RLK-Pelle_LRK10L-2 families) per family (Supplementary Table 10).

Qualitatively, the families scrutinized showed some specificities of enriched *bona fide* CCREs and associated TFs (Figure 6 and Supplementary Table 10). Otherwise, it was observed that enriched *bona fide* CCREs associated with C2H2 transcription factor class (of which the Dof-type family is part) were present in all families studied, except in RLK-Pelle_CrRLK1L-1 (Figure 6 and Supplementary Table 10).

### Transcriptomics of VuPKs Under Stressful Conditions: General Aspects

The metrics of all RNA-Seq libraries are available in the Supplementary Appendix 2.

CpGC RNA-Seq libraries were used to analyze the VuPK expression under unfavorable conditions. Specifically, three distinct conditions were studied:

(1) Mechanical injury followed by CABMV virus inoculation.
(2) Mechanical injury followed by CPSMV virus inoculation.
(3) Root dehydration.

Encompassing all assays, 5,310 VuPK candidates were identified by iTAK tool. After phenetic analysis processing, 5,195 *bona fide* VuPKs were filtered. From these, 1,178 (∼23%) presented differential expression (up- or down-regulation) in at least one treatment of the three assays performed (Supplementary Table 11). Of these, 819 (∼15%) VuPK representatives (of 18 groups and 105 families) were up-regulated (Supplementary Table 11).

**FIGURE 7:**
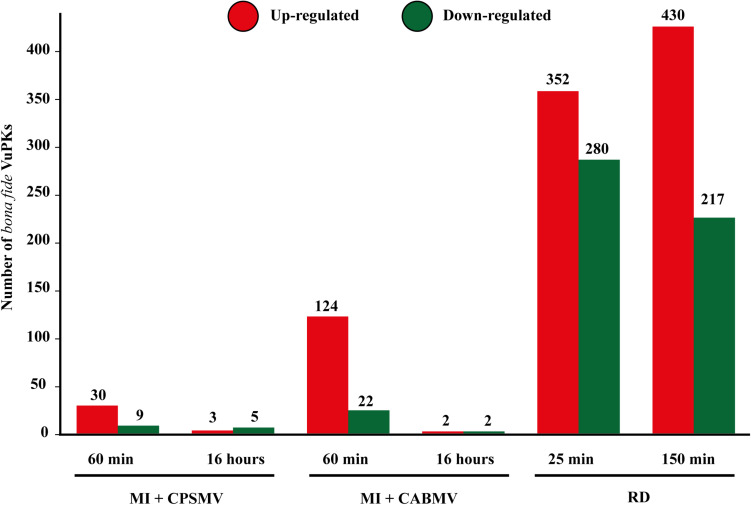
Amounts of *bona fide* VuPKs differentially expressed in the studied treatments. VuPKs, *Vigna unguiculata* protein kinases; MI, mechanical injury; CABMV, cowpea aphid-borne mosaic virus; CPSMV, cowpea severe mosaic virus; RD, root dehydration; min, minutes.

The numbers of differentially expressed VuPKs for each assay are shown in Figure 7, indicating a substantial discrepancy in the up-regulated VuPK amounts under different conditions/treatments. Considering the combined stresses ‘mechanical injury + virus inoculation’ in the leaves, VuPKs tend to be up-regulated in the initial times (Figure 7). In turn, root dehydration stress recruited a higher VuPK transcriptional response in cowpea, presenting an increased tendency to an up-regulation at the later treatment time after stress application (Figure 7).

#### VuPKs Transcriptomics on the “Mechanical Injury and Virus Inoculation” Assays: Crosstalk and Specificities

**FIGURE 8:**
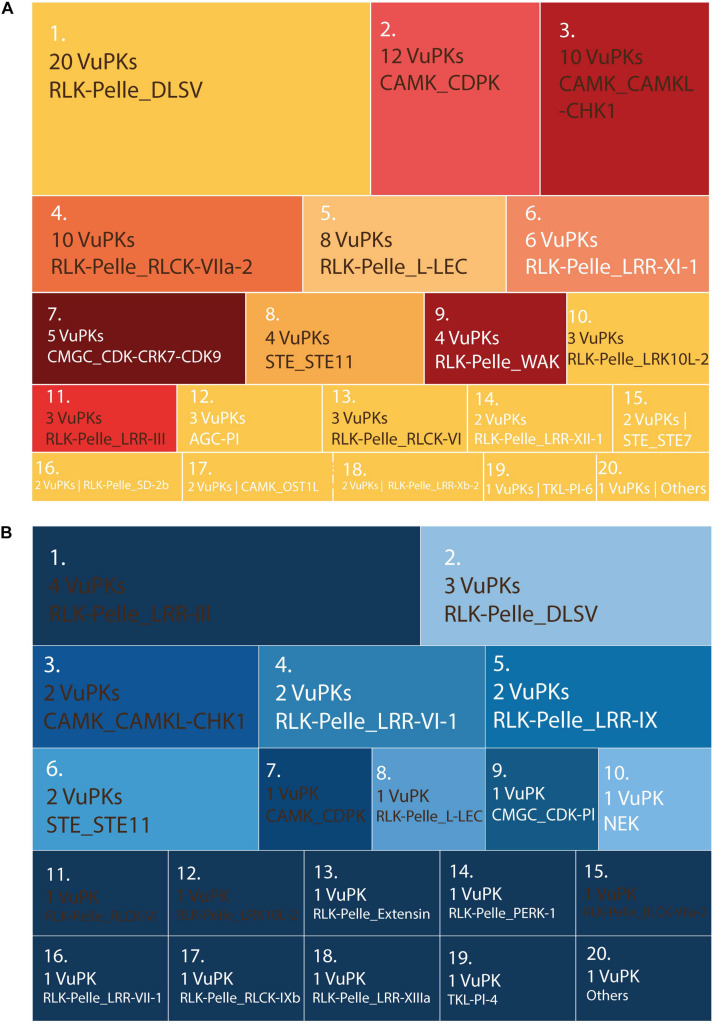
Treemap containing the ranking of the 20 most up-regulated *bona fide* VuPK families on the treatments (60 min and/or 16 h) of the following assays: **(A)** mechanical injury + CABMV inoculation and **(B)** mechanical injury + CPSMV inoculation. Families highlighted in brown occur in **(A)** and **(B)**—differences in quantities indicate differences in the VuPKs that make up the sets. Families highlighted in white occur only in **(A)** or only in **(B)**. VuPKs, *V. unguiculata* protein kinases; CABMV, cowpea aphid-borne mosaic virus; CPSMV, cowpea severe mosaic virus; others in **(A)** (25 families with one up-regulated VuPK each); others in **(B)** (four families with one up-regulated VuPK each).

There were no enriched GO terms for the up-regulated VuPKs set in each ‘mechanical injury and virus inoculation’ assay. Thus, other strategies were used to characterize and compare these VuPKs. The up-regulated kinome analysis in ‘mechanical injury + CABMV inoculation’ and ‘mechanical injury + CPSMV inoculation’ showed us that, despite the presence of some similar families, their up-regulated kinome was peculiar to each assay (Figure 8 and Supplementary Table 11).

The RLK-Pelle_DLSV family (with the largest number of up-regulated kinases for ‘mechanical injury + CABMV inoculation’ and the second for ‘mechanical injury + CPSMV inoculation’; Figure 8) represents well the peculiarity mentioned previously. Despite appearing in the transcriptome of up-regulated VuPKs in both conditions mentioned, most up-regulated kinases were restricted to the ‘mechanical injury and CABMV inoculation’ assay. This family presented crosstalk restricted to two up-regulated transcripts (Supplementary Table 11). Another example is the CAMK_CDPK family. Although it appears as up-regulated transcripts in both comparisons, they regard different isoforms (Supplementary Table 11), that is, it did not display common members up-regulated. Overall, only 12 up-regulated transcripts, associated with eight VuPK families (i.e., RLK-Pelle_DLSV, RLK-Pelle_RLCK-VI, RLK-Pelle_LRR-Xb-2, STE_STE11, CAMK_CAMKL-CHK1, CMGC_CDK-CRK7-CDK9, RLK-Pelle_LRR-III, and RLK-Pelle_LRR-XII-1) exhibited crosstalk among the ‘mechanical injury + virus inoculation’ treatments (Supplementary Table 11). This number is relatively low considering the set of up-regulated transcripts. Thus, it is suggested that the expression of VuPKs in cowpea was virus dependent and/or genotype dependent since these elements vary between treatments.

#### VuPK Response to Root Dehydration Stress

Root dehydration stress showed a higher number of differentially expressed VuPKs (Figure 7) compared to the other assays. Thus, GO enrichment analyses were possible, with consequent identification of enriched terms. In the present work, we chose to study enriched GO terms related to the ‘biological process’ category, aiming to understand the cellular processes activated by the up-regulated VuPKs.

**FIGURE 9:**
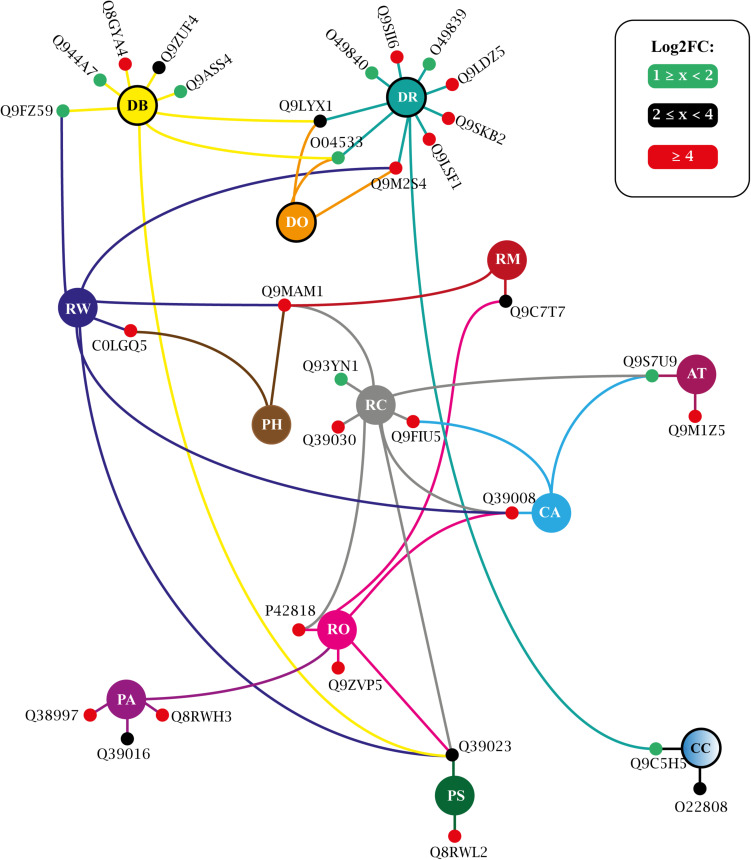
Biological process enriched for T25 up-regulated VuPKs showing the interaction network (colored lines) of the associated elements (proteins). The colored dots (green, black, and red) in the protein Uniprot ID correspond to the fold-change log (log_2_FC) found in the present study and are according to the presented scale. The colors of the lines are associated with the colors of the central nodes that contain the abbreviation of the enriched biological process. Different colored lines connected to a given protein (colored dots—red, black, or green) indicate that this protein participates in different enriched processes. Central nodes with the border highlighted in black represent GO terms associated with the biotic stress response. T25, ‘RD25 vs. Cont.25’; GO terms enriched (AT, auxin transport; CC, cellular response to chitin; CA, cold acclimation; DR, defense response; DB, defense response to bacterium; DO, defense response to oomycetes; PA, positive regulation of abscisic acid-activated signaling pathway; PS, positive regulation of response to salt stress; PH, potassium ion homeostasis; RC, response to cold; RM, response to mannitol; RO, response to osmotic stress; RW, response to wounding; RD, root dehydration; Cont., control). Adapted from NeVOmics tool output.

**FIGURE 10:**
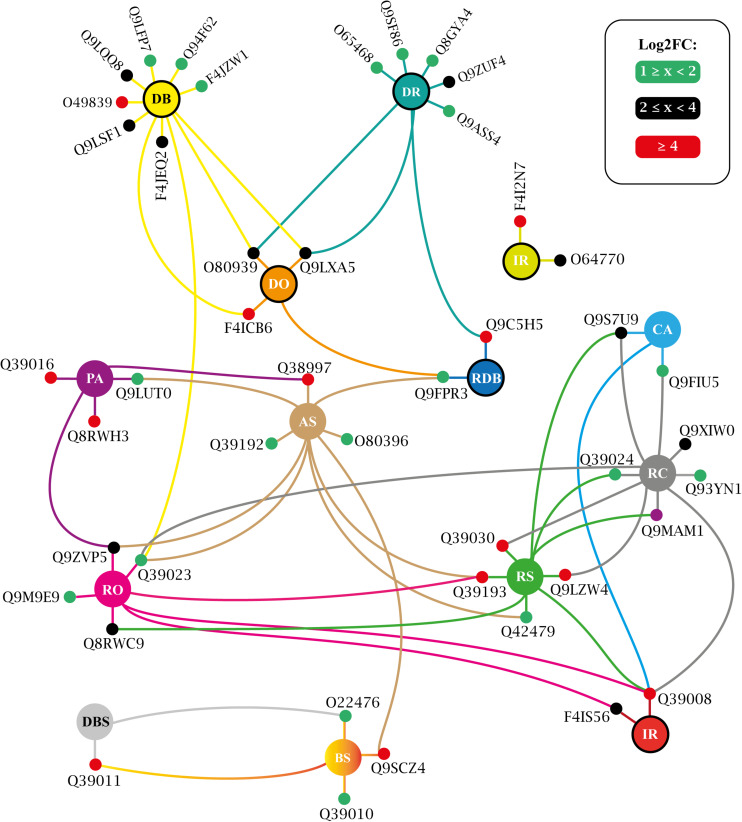
Biological process enriched for T150 up-regulated VuPKs showing the interaction network (colored lines) of the associated elements (proteins). The colored dots (green, black, and red) in the protein Uniprot ID correspond to the fold-change log (log_2_FC) found in the present study and are according to the presented scale. The colors of the lines are associated with the colors of the central nodes that contain the abbreviation of the enriched biological process. Different colored lines connected to a given protein (colored dots—red, black, or green) indicate that this protein participates in different enriched processes. Central nodes with the border highlighted in black represent GO terms associated with the biotic stress response. T150, ‘RD150 vs. Cont.150’; GO terms enriched (AS, abscisic acid-activated signaling pathway; BS, brassinosteroid mediated signaling pathway; CA, cold acclimation; DR, defense response; DB, defense response to bacterium; DO, defense response to oomycetes; DBS, detection of brassinosteroid stimulus; IR, innate immune response; PA, positive regulation of abscisic acid-activated signaling pathway; RDB, regulation of defense response to bacterium; RC, response to cold; RO, response to osmotic stress; RS, response to salt stress; RD, root dehydration; Cont., control). Adapted from NeVOmics tool output.

More than three dozen enriched terms were found for T25 (Supplementary Table 12) and for T150 (Supplementary Table 13). A large portion of these covers generic processes traditionally associated with protein kinases, such as ‘protein phosphorylation,’ ‘signal transduction,’ ‘stress-activated protein kinase signaling cascade,’ etc. (Supplementary Tables 12, 13). Such obviousness was eliminated, and an interaction network was adapted, presenting the biologically informative enriched GO terms and the relationship between their constituent elements. For T25, such a network is shown in Figure 9 and in Figure 10 for T150.

The study of biological processes potentially activated by up-regulated VuPKs included important actions implemented during the adaptation process to root dehydration in the tolerant cowpea genotype. In both treatments (T25 and T150), enriched terms associated with the ABA hormone were observed, such as ‘positive regulation of abscisic acid-activated signaling pathway’ (PA) (Figures 9, 10), indicating a role for the up-regulated VuPKs in this process. Some component elements of the ‘PA’ term have been associated with heavily modulated transcripts (red dots; Figures 9, 10) as Q38997 (T25; Figure 9), an SNF1-related protein kinase, and Q8RWH3 (T150; Figure 10) and YAK1 (a dual-specificity tyrosine-regulated protein kinase). This high modulation was observed for several elements that make up other enriched biological processes (red dots; Figures 9, 10), which have an important impact on plant physiology (see “VuPK Transcriptomics Under Stressful Conditions” in the “Discussion” section).

There were also potentially multi-stress VuPKs. These VuPKs have been characterized as responsive to other stress types, such as those associated with enriched GO terms ‘response to cold’ (RC), ‘response to osmotic stress’ (RO), and related to the salt stress response [as ‘positive regulation of response to salt stress’ (PS) and ‘response to salt stress’(RS)] (Figures 9, 10). In both treatments, VuPKs were still associated with terms related to the response to biotic stresses (central nodes with the black border highlighted in Figures 9, 10), including ‘defense response’ (DR), ‘defense response to bacterium’ (DB), and ‘defense response to oomycetes’ (DO).

Specifically for the T25 treatment, VuPKs potentially activated processes related to auxin transport (AT) as well as those related to potassium ion homeostasis (PH) (Figure 9). For T150 treatment, up-regulated VuPKs were involved in signaling processes associated with the Brassinosteroid hormone due to the presence of enriched GO terms such as ‘brassinosteroid mediated signaling pathway’ (BS) and ‘detection of brassinosteroid stimulus’ (DBS) (Figure 10).

In terms of categorizing the searched VuPK families, 100 of them had at least one up-regulated transcript (Supplementary Table 11) in T25 or T150, demonstrating the complexity of cowpea kinome associated with the root dehydration response. The families RLK-Pelle_DLSV (74), CAMK_CAMKL-CHK1 (45), and CAMK_CDPK (36) were the most abundant. On the crosstalk of up-regulated VuPKs among the three different assays, only five transcripts associated with four different families (RLK-Pelle_LRR-Xb-2, STE_STE11, CAMK_CAMKL-CHK1, and RLK-Pelle_LRR-III) presented this feature (Supplementary Table 11).

#### RNA-Seq Data Validation by qPCR

To analyze the reliability and robustness of *in silico* gene expression (RNA-Seq libraries), a VuPK sample was also scrutinized by qPCR. Ten up-regulated VuPKs (of which three exhibited crosstalk response among the three performed assays) were randomly chosen to analyze the RNA-Seq data quality. The transcript sample covered eight different kinase families (Supplementary Table 1).

The primer pairs (for target transcripts and reference genes) had an amplification efficiency ranging from 90.4 to 109.8% (Supplementary Table 1). The specificity was confirmed by the presence of a single peak in the melting curves (Supplementary Figure 3). All the 20 combinations (made up by 10 VuPK targets analyzed in different stresses and treatment times) scrutinized by qPCR confirmed the RNA-Seq results (Supplementary Figure 4 and Supplementary Appendix 3).

## Discussion

### Genomic Aspects of the Cowpea Kinome: A General View

The recent publication of the cowpea genome (Lonardi et al., 2019) opened up new possibilities and perspectives for the analysis of cowpea kinases, making the present work a pioneer for the mentioned crop. The presence of 1,293 *bona fide* VuPKs was found in the cowpea genome, corresponding to about 4% (1,293 out of 31,948) of the cowpea protein-coding loci. Compared to two recently studied kinomes, this value was similar to that found in grapevine (3.7%; Vitaceae; Zhu et al., 2018a) and superior to that reported in pineapple (2.8%; Bromeliaceae; Zhu et al., 2018b). This number is similar to that found in *A. thaliana* (3.6%; Zulawski et al., 2014). For the Leguminosae family, to which the cowpea belongs, data is available only for soybean, with 4.67% of its protein-coding loci associated with kinases (Liu et al., 2015), a higher proportion than that found in the present study.

All the 20 groups of kinases detectable by iTAK tool were found in cowpea. Of the 150 families, 118 were recognized. RLK-Pelle (about 70% of the analyzed kinome) was the group with the greatest abundance and diversity of families (Figure 1A). This occurred in several other species, such as soybean (Leguminosae family; Liu et al., 2015), grapevine (Vitaceae; Zhu et al., 2018a), pineapple (Bromeliaceae; Zhu et al., 2018b), and *Eucalyptus grandis* (Myrtaceae; Lehti-Shiu and Shiu, 2012) among others. The scientific literature indicates that the high number of RLK-Pelle kinase coding loci in genomes is characteristic of land plants (Lehti-Shiu et al., 2009, 2011). The reduced RLK-Pelle sizes in animals and green algae are likely evidence that the group was very small before the chlorophyte lineage diverged from land plants and related charophyte algae (Hedges, 2002). The great group expansion has been postulated to be crucial for plant-specific adaptations because its genes play roles ranging from growth regulation to defense response [for a review, see Boller (2012) and Stahl and Simon (2012)].

Besides RLK-Pelle, the most abundant kinase groups were CAMK (89), CMGC (78), TKL (63), STE (49), and AGC (42) (Figure 1A). Altogether 95% (1,229/1,293) of the cowpea kinome consist of these VuPKs. The remaining groups, representing 5% of all cowpea kinome, individually presented a small number of loci (≤16; Figure 1A). In this context, we highlight the groups BUB and Gr. PL-2 (with one locus each), IRE1, PEK, TTK, and ULK (two loci each), WEE, SCY1, Gr. PL-4, Gr. PL-3, and AUR (three loci each). Loci encoding these kinase groups represent hot spots for gene silencing assays and functional characterization. For the other groups, such analyses are hampered due to the high content of duplicated coding loci (see section “The VuPK Gene Duplication Mechanisms in Cowpea Genome” below).

Considering the families, RLK-Pelle_DLSV (174) was the most abundant (Figure 1A). This characteristic (high RLK-Pelle_DLSV abundance) remains at the taxonomic genus or even intra-family levels when analyzing some plant groups. In the first case, it was observed that the RLK-Pelle_DLSV family also contained the largest members among the cotton species (*Gossypium raimondii*, *Gossypium arboretum*, *Gossypium hirsutum*, and *Gossypium barbadense*) (Yan et al., 2018). In the second case, data indicate that, for the Leguminosae family [e.g., soybean and *M. truncatula* (Lehti-Shiu and Shiu, 2012; Liu et al., 2015), besides cowpea herein analyzed], RLK-Pelle_DLSV is also the most abundant family. According to Liu et al. (2015), *A. thaliana*, rice (*Oryza sativa*), and maize (*Z. mays*) follow the same abundance tendency. The exceptions regard the grapevine (Zhu et al., 2018a) and pineapple (Zhu et al., 2018b) kinomes.

Considering the spatial distribution, it was observed that, despite the high quantity and diversity of VuPKs on chromosome ‘3’ (Vigun03g; Figure 1B), a random pattern was found, being distributed throughout all chromosomes (and even scaffolds). A similar fact is observed concerning soybean (Liu et al., 2015), grapevine (Zhu et al., 2018a), and pineapple (Zhu et al., 2018b).

### VuPKs: From Gene Structure to Protein Properties

We observed that the proportion of intronless VuPK genes (13.5%) was much less than that of total VuPK genes with introns (86.5%), suggesting that intron gain has significantly contributed to the structural divergence of the VuPKs. A similar fact was found in soybean (Liu et al., 2015), a plant from the same cowpea family. However, about ∼ 67% (127/190 comparisons; Supplementary Table 8) of tandem duplicated genes have identical intron numbers. This result indicates that, in cowpea genome, paralogous arising from duplication by other mechanisms may be more subjected to an expansion of these quantitative structures. A possible explanation for this fact would be that tandem duplicated paralogous are located in an identical genomic context. This would be a restrictive factor, maintaining the number of introns. In accordance with the statement above, the high rate of VuPK duplication of the ‘dispersed’ type (the main apparatus for the VuPK expansion in the cowpea genome; Figure 2) would not suffer the restrictions of a similar genomic context, potentiating the high heterogeneity of the number of introns between VuPK members of the same family. However, such an observation needs further investigation.

Another parameter of biological importance is associated with the number of kinase domains. Usually, domain sites are responsible for a particular function or interaction, contributing to the overall protein role. About 95% of VuPK families have more than one domain in their protein structure. Additionally, at the intra-familial level, domain number and type show little variation. Members in each family generally shared similar conserved domain arrangements, suggesting a common evolutionary history, as reported for pineapple by Zhu et al. (2018b). Ding et al. (2009) observed that kinases could form homo- or heterodimers. Thus, the multiple kinase domain structure of VuPKs may be functionally equivalent to dimerized VuPKs, as suggested by Liu et al. (2015). These authors also suggested that the cooperative activity of multiple kinase domains may be required for specific substrates.

In respect of subcellular location, it was observed that almost all of the VuPKs allocated in the plasma membrane were from the RLK-Pelle group (Supplementary Table 6). These proteins play important roles in perceiving the extracellular ligands and activating the downstream pathways [for a review, see Osakabe et al. (2013)]. They can act as transmembrane proteins, with extracellular receptor domains and intracellular kinase domains (Minkoff et al., 2017). The remaining groups, in addition to some members of the RLK-Pelle group, were distributed throughout the vacuole, chloroplast, mitochondria, cytoplasm, extracellular space, and nucleus. This demonstrates the dynamics and plasticity of these proteins in cowpea, suggesting their action at multiple intracellular sites.

Finally, concerning the VuPK biochemical properties, pI and WM, it was observed that they were variants, even within the family (Supplementary Table 6). This result contrasts with those available for this type of analysis. For instance, in grapevine, Zhu et al. (2018a) observed that such parameters within the same family were generally similar.

### The VuPK Gene Duplication Mechanisms in Cowpea Genome

The present study demonstrated that three main mechanisms, with different levels of performance, were responsible for the expansion of this gene superfamily (Figure 2): tandem, proximal, and dispersed gene duplications. Such mechanisms occurred mainly in the RLK-Pelle group, reinforcing the trend found in land plants. As mentioned earlier, this group expanded continuously throughout land plant evolution. Along the evolutionary scale, it is observed that the early diverging bryophyte species, *Physcomitrella patens* (moss), has 329 RLK-Pelle (Lehti-Shiu et al., 2009). In contrast, there are 610 RLK/Pelle genes in *A. thaliana* (Shiu and Bleecker, 2001), 872 in grapevine (Zhu et al., 2018a), and 1,418 in soybean (Liu et al., 2015).

The most abundant family in the RLK-Pelle group was the RLK-Pelle_DLSV, which comprises about 20% of that group’s members. There were also families with very small numbers, such as RLK-Pelle_C-LEC and RLK-Pelle_Singleton (one locus each; Supplementary Table 4). This indicates that, in cowpea, selective forces act differently on different kinase families. In the legume soybean, the size of the RLK-Pelle subfamilies was also highly variable (Liu et al., 2015).

The dispersed duplication mechanism was mainly responsible for the expansion of the cowpea kinome. In all groups analyzed, the mentioned mechanism was predominant, being the only one responsible for the expansion of 13 specific groups (Figure 2). According to Wang et al. (2012), copies duplicated by dispersion mechanism might arise from transposition, such as ‘replicative transposition,’ ‘non-replicative transposition,’ or ‘conservative transposition.’ The dispersed duplicated genes are prevalent in different plant genomes (Wang et al., 2016). The third most active duplication mechanism was the ‘proximal’ type (Figure 2). This was closely associated with the duplication of the RLK-Pelle group. About 94% of such gene duplications occurred in that group. A gene duplicated by this mechanism might arise from small-scale transposition or tandem duplication and insertion of some other genes (Wang et al., 2012).

Both aforementioned duplication mechanisms—responsible for duplicating about 76% of the cowpea kinome—could be influenced by transposable elements. It is known that all families of transposons are capable of duplicating genes or gene fragments, and such events have been detected in a broad spectrum of organisms (Cerbin and Jiang, 2018). About 39% of the cowpea genome comprises transposable elements, which are largely responsible for the size differences of the genomes of species of the genus *Vigna* (Lonardi et al., 2019).

There was the tandem duplication mechanism also, which occurred by expanding VuPKs from five different groups (CAMK, CMCG, RLK-Pelle, STE, and TKL). This mechanism represented the second largest expansion force of the cowpea kinome, covering 309 VuPKs. However, the largest tandem duplication events involved members of the RLK-Pelle group, both in tandem gene extension and in quantity (it covered 180 of the 190 duplicated gene pairs) (Supplementary Table 8). In cowpea, tandem duplicated genes accounted for ∼24% of the kinome. This percentage is one of the largest reported so far, being higher than that in *Arabidopsis* (9.5%; Zulawski et al., 2014), soybean (10.6%; Liu et al., 2015), pineapple (12,5%; Zhu et al., 2018b), and maize (17.2%; Wei et al., 2014) but lower than that of grapevine (∼34%; Zhu et al., 2018a).

Finally, the *K*_*a*_/*K*_*s*_ substitution rate ratio of the tandem duplicated genes (190 gene pairs) was analyzed. This ratio is an indicator of the selection history of genes or gene regions. The vast majority (91%) of the analyzed gene pairs have *K*_*a*_/*K*_*s*_ < 1, that is, they are under purifying selection, which preserves the amino acid sequences. This finding also suggests that duplicated pairs may have some functional redundancy. This mechanism potentially compensates for stochastic loss-of-function in specific gene family members or paralogous in the course of evolution.

### VuPK Comparative Genomics: From Punctual Orthology to Syntenic Blocks

A total of 64 out of 93 genomes analyzed presented orthology indexes ranging from 30% (*M. polymorpha*; Marchantiaceae) to 88% (*P. vulgaris*; Leguminosae) of the 1,293 *bona fide* VuPKs (Figure 3). This demonstrates a conservation gradient of VuPKs, opening new perspectives of studies concerning their function and influence on the species in which they were conserved.

Leguminosae members had the three highest orthology indexes (Figure 3), a fact already expected since orthology indexes reflect the phylogenetic distance between species (Kaló et al., 2004). The greatest orthology index occurred with common bean, which is considered a cowpea sister group, and both present some divergence when compared to soybean (Lei et al., 2016).

The analysis of orthologous loci among ‘cowpea *versus* soybean’ and ‘cowpea *versus* common bean’ showed that orthologous VuPKs participated in a greater number of syntenic blocks with soybean. This was because ‘cowpea *versus* soybean’ syntenic blocks were more fragmented, smaller, and more numerous. The comparison ‘cowpea *versus* common bean’ revealed longer, more complex, and a lower number of syntenic blocks. According to Ganko et al. (2007), ‘dispersed’ duplicates, like most of the VuPKs, are paralogous that are neither near each other on chromosomes nor show conserved synteny. However, the predominantly dispersed nature of the VuPK duplication in cowpea genome did not preclude their anchoring in syntenic blocks compared with the other analyzed legumes.

### CCRE Enrichment and Associated Transcription Factors on RLK-Pelle Group Gene Promoters

A total of 62 *bona fide* CCREs associated with 49 different TFs were identified (Figure 6 and Supplementary Table 10). The types and quantity of *bona fide* CCREs varied in the different families analyzed, indicating that they participate in different processes coordinated by different transcription factors. This is in line with the heterogeneous range of functions reported for kinases: from abiotic stress response (Ye et al., 2017) and developmental processes (Stahl and Simon, 2012) to plant defense (Lehti-Shiu et al., 2009). Interestingly, the class of C2H2 transcription factors was closely associated with families in the RLK-Pelle group, being enriched in eight of the nine families in the analyzed group. These TFs function as key transcriptional regulators in plant responses to a broad spectrum of stress conditions, including extreme temperatures, salinity, drought, oxidative stress, excessive light, and silique shattering among others [for a review, see Wang et al. (2019)]. Otherwise, some members of the C2H2 class, such as the Dof-type (also enriched for some of the families analyzed), are involved in pathogen defense, such as viruses (Kang et al., 2016). In addition to suggesting VuPK participation in the referred processes, such data assist in characterizing the transcriptional dynamic of these proteins under unfavorable conditions.

### VuPK Transcriptomics Under Stressful Conditions

The qPCR assay corroborated the *in silico* RNA-Seq gene expression data for the scrutinized targets. This indicates good practices during the sequencing and synthesis of the analyzed libraries. It also reinforces the rigor of transcriptome assembly and statistical methods of the differential expression analysis. All these elements together ensure the robustness of the information available in the present work.

We propose that the VuPK transcriptional orchestration occurs in a stress-dependent and/or genotype-dependent and/or tissue-dependent manner since the crosstalk was relatively reduced, and the mentioned elements (stress, genotype, and tissue) varied among the three assays (see “Materials and Methods” section). To date, only tissue-dependent and stress-dependent kinase expression has been reported for angiosperms. Concerning the first one, Zhu et al. (2018a) reported that the tissue-specific RNA-Seq analysis of pineapple kinase genes showed various expression patterns. Regarding the second case, Zhu et al. (2018b) reported for grapevine that the expression of family members as CMGC_CDK-Pl, RLK-Pelle_LRR-XIIIb, and RLK-Pelle_RLCK-IXa were down-regulated in response to salt, PEG, and drought treatments but that these were up-regulated in response to heat stress; Yan et al. (2018) also reported stress-specific up-regulation for *G. hirsutum*.

In terms of VuPK families, the expression of RLK-Pelle_DLSV and CAMK_CAMKL-CHK1 stood out. These VuPK families are among the three most up-regulated in all the transcriptomics assays performed in the present work. There are reports of up-regulation of CAMK_CAMKL-CHK1 in response to stresses that cause dehydration, such as salt, PEG, and drought (Zhu et al., 2018b). However, there was still no information in the literature about the participation of isoforms of these families in response to the combination ‘mechanical injury + virus inoculation’. For RLK-Pelle_DLSV, there were still no reports of its participation in plant stress response (abiotic or biotic). Such results raise certain members of these two families to a higher importance level for functional characterization tests. Additionally, they can become biotechnological targets of great interest in cowpea and related species.

Specifically, in relation to root dehydration stress, 100 families of VuPK had at least one up-regulated isoform throughout the assay. GO terms associated with the positive regulation of the ABA hormone (‘PA’ and ‘AS’), a key hormone in response to drought in plants, were enriched in T25 (Figure 9) and/or T150 (Figure 10). According to Fernando and Schroeder (2016), the role of ABA in drought and salt stress is twofold: water balance and cellular dehydration tolerance. Proteins such as Q38997 (Figure 9), an SNF1-related protein kinase, were among the most modulated for the ‘PA’ term in T25. Umezawa et al. (2004) indicated that a SNF1-related protein kinase is a positive regulator of drought tolerance in *Arabidopsis* roots. Concerning T150, the protein Q8RWH3 (Figure 10), a YAK1 (a dual-specificity tyrosine-regulated protein kinases), was one of the most modulated for the term “PA.” Results of the work by Kim et al. (2016) indicated that AtYak1 plays a role as a positive regulator in ABA-mediated drought response in *Arabidopsis*.

It was also observed that GO terms associated with the biotic stress response were enriched in both treatments (central nodes with the border highlighted in black; Figures 9, 10). This may be related to a natural strategy of the Pingo de Ouro cultivar. Prasch and Sonnewald (2013) suggested that abiotic stress factors significantly altered turnip mosaic virus-specific signaling networks, which led to the deactivation of defense responses and a higher susceptibility of *Arabidopsis* plants. Sinha et al. (2019), in turn, also observed an increased incidence of fungal diseases in chickpea plants under severe drought stress compared to those in well-irrigated field conditions. Thus, the studied cultivar may use defense mechanisms during an unfavorable abiotic condition, aiming to keep on constant alert against different stressors, with VuPKs being important players in the perception and signaling process.

Terms such as ‘response to cold’, ‘response to osmotic stress’, and those related to salt stress response (RS and PS) (Figures 9, 10) were also enriched in T25 and/or T150. This enrichment was possibly because the mentioned conditions (cold and osmotic and salt stress) strongly alter the water availability in the environment. Chinnusamy et al. (2007) observed that cold temperature affects membrane fluidity and disrupts nucleic acid and protein structures besides hindering water and nutrient uptake. Torres et al. (2007) suggest that osmotic and salt stress should be treated as a group of factors imposing alterations in the plant water status. Thus, the up-regulated VuPKs associated with these terms could act in processes related to signaling and response to water availability. An example is Q39193 (a SnRK2), one of the most modulated for the term ‘response to salt stress’ (RS) (Figure 10). According to Fujita et al. (2009), SnRK2 kinases are the main positive regulators of ABA signaling in response to water deficit stress in *Arabidopsis*.

The enriched terms exclusive to each treatment, ‘AT’ (‘auxin transport’) and ‘PH’ (‘potassium ion homeostasis’), stand out for T25 (Figure 9). Regarding ‘AT’, the scientific literature shows that auxin plays an essential role during abiotic stress-induced changes in the root. Developmental modifications to root system architecture (RSA) is vital for tolerance to drought and high salt stresses. It has long been established that RSA requires the phytohormone auxin. Auxin transport in plants also has received much attention [for a review, see Korver et al. (2018)]. The enrichment of the term ‘PH,’ in turn, suggest that up-regulated VuPK may act in the referred process. Maintaining adequate plant potassium is critical for plant drought tolerance. A close relationship between these two factors has been demonstrated (Wang et al., 2013).

Finally, terms associated with brassinosteroid (a class of plant steroidal hormones), ‘DBS’ and ‘BS,’ were specifically enriched from T150 up-regulated VuPKs (Figure 10). From the past decade, additional roles for brassinosteroids have emerged, particularly relating to stress tolerance (Ahammed et al., 2020). However, the two most modulated VuPKs for those terms, Q39011 (shaggy-related protein kinase) and Q9SCZ4 (receptor-like protein kinase FERONIA), are negative regulators of brassinosteroid response and brassinosteroid signal transduction pathway, respectively. Such observations suggest that this steroidal hormone does not actively participate in cowpea’s response to root dehydration stress. However, further investigations to confirm this observation are necessary.

Our results provide a scientific foundation for the comprehensive understanding of VuPK omics aspects. In this work, we connect the VuPKs with the state-of-the-art knowledge offering a general picture of this genomic presence, evolutionary aspects, and its participation in the cowpea response to biotic and abiotic stresses. Extensive works of functional characterization (transgenic or gene silencing approaches) will be the next steps to be performed to determine the specific roles (activation of signaling process or metabolic pathways, etc.) of VuPKs in unfavorable conditions. Comprehensive knowledge of these actors could aid the current knowledge about the cowpea molecular physiology. Additionally, these proteins could become a useful resource in the quest to generate stress-tolerant/resistant, high-yielding cowpea varieties.

## Data Availability Statement

The datasets generated for this study can be found in online repositories. The names of the repository/repositories and accession number(s) can be found below: https://www.ncbi.nlm.nih.gov/, Root dehydration | BioProject ID: PRJNA605156; Mechanical injury + CABMV | BioProject ID: PRJNA655993; Mechanical injury + MV | BioProject ID: PRJNA656211.

## Author Contributions

AB-I conceived and designed the experiments. JF-N and AB performed the experiments. JF-N, MS, EK, and AB performed the laboratory experiments and analyzed the data. DM, GB, and JB-N provided support for the RNA-Seq assembly and quality analyses. AB-I obtained the financial resources. AB-I and JF-N wrote the manuscript. All authors read and approved the manuscript.

## Conflict of Interest

The authors declare that the research was conducted in the absence of any commercial or financial relationships that could be construed as a potential conflict of interest.

## Footnotes

1 https://gist.github.com/darencard/fcb32168c243b92734e85c5f8b59a1c3

2 https://phytozome.jgi.doe.gov

3 http://meme-suite.org/tools/meme

4 http://meme-suite.org/tools/tomtom

5 https://www.uniprot.org/

6 https://github.com/TransDecoder/TransDecoder/wiki

## Supplementary Material

Supplementary Figure 1 Implemented procedures and experimental design for root dehydration assay. **(A)** Growth conditions and stress application in tanks with hydroponic solution, in a controlled environment, prior to the application of root dehydration treatment (left) by withdrawing the nutrient solution from the roots of the treated plants (right). **(B)** Experimental design with three replicates “A, B, C.” Treatment time: 25 and 150 min after removal of hydroponic solution (RD25’ and RD150’, respectively) and their respective non-stressed controls (Cont.25’ and Cont.150’, respectively). RD, root dehydration; Cont., control;’, minutes.

Supplementary Figure 2 Implemented procedures and experimental design for the ‘mechanical injury + CABMV inoculation’ and ‘mechanical injury + CPSMV inoculation’ assays. **(A)** Growth conditions and stress application in a controlled environment (right) and the procedures for stress application (left). **(B)** Experimental design with three replicates “A, B, C.” Treatment time: 60 min and 16 h after stress application (‘MI + Vir60’ and ‘MI + Vir16h,’ respectively) and their respective non-stressed controls (Cont.60’ and Cont.16h, respectively). MI, mechanical injury; Vir, virus (CABMV or CPSMV); Cont., control;’, minutes; h, hours.

Supplementary Figure 3 Neighbor-joining distance tree illustrating VuPK phenetic relationships. Values at nodes correspond to the bootstrap values. UNK (unknown) refers to VuPKs whose phenetic grouping did not occur with their peers (based on iTAK classification).

Supplementary Figure 4 qPCR results for a sample of up-regulated VuPKs obtained from the RNA-Seq assays. **(A)** Mechanical injury (MI) + CABMV (cowpea aphid-borne mosaic virus) inoculation, **(B)** MI + CPSMV (cowpea severe mosaic virus) inoculation, and **(C)** root dehydration (RD). The asterisk indicates being differentially expressed at *p* < 0.05. Black circles: 1—up-regulated crosstalk response among MI + CABMV, MI + CPSMV, and RD assays; 2—up-regulated crosstalk response between MI + CABMV and MI + CPSMV assays; and 3—up-regulated crosstalk response between MI + CPSMV and RD assays.

Supplementary Table 1 Reference genes and target VuPKs used in the relative expression analysis, presenting the primer pair sequences, melting temperature of the amplicon, and primer pair efficiency values.

Supplementary Table 2 iTAK software output, indicating the VuPKs searched in the cowpea genome as well as the types of domain and their quantity per analyzed sequence, in addition to alignment values for the implemented Hidden Markov Model.

Supplementary Table 3 *Bona fide* VuPKs with their respective kinase families as well as indication of the unknown kinases, whose identity was not corroborated by the two adopted categorization strategies.

Supplementary Table 4 Characterization of the 118 kinase families of the cowpea genome, indicating the number of coding loci and 12 parameters that define the average gene structure of their *bona fide* VuPKs constituent.

Supplementary Table 5 Individual characterization of the 1,293 *bona fide* VuPKs searched in the cowpea genome, indicating their respective coding loci as well as 12 parameters that define their gene structure.

Supplementary Table 6 Subcellular location and protein properties [isoelectric point (pI) and molecular weight (MW)] of the *bona fide* VuPKs searched in the cowpea genome (#N/D, not computed value.

Supplementary Table 7 Mechanisms of origin of 1,293 loci encoding *bona fide* VuPKs in the cowpea genome. The asterisk indicates duplication mechanism.

Supplementary Table 8 *K*_*a*_, *K*_*s*_, and *K*_*a*_/*K*_*s*_ ratio values and number of introns for the 190 gene pairs of *bona fide* VuPKs originated *via* tandem duplication.

Supplementary Table 9 Species with genome deposited in the Phytozome database, presenting their respective plant family and the number of orthologous loci to the *bona fide* VuPKs searched in the cowpea genome.

Supplementary Table 10 *Bona fide cis*-regulatory candidate elements anchored in the promoter regions of genes encoding VuPKs from different families in the RLK-Pelle group, with information on their consensus motif and associated transcription factors (Matrix JASPAR ID). CCREs, candidate *cis*-regulatory elements.

Supplementary Table 11 Transcriptional regulation of the 1,178 isoforms of *bona fide* VuPKs differentially expressed in at least one of the treatments performed in the present work. NEC, not expressed in this contrast; const., constitutive expression; UR, up-regulated expression; DR, down-regulated expression; log2FC, log of the expression ratio obtained from respective treatment vs. control contrast.

Supplementary Table 12 Complete NeVOmics tabular output for biological process enrichment analysis of T25 *bona fide* VuPKs up-regulated.

Supplementary Table 13 Complete NeVOmics tabular output for biological process enrichment analysis of T150 *bona fide* VuPKs up-regulated.

Supplementary Appendix 1 Melting curves for target transcripts used in the present study.

Supplementary Appendix 2 Metrics for cowpea RNA-Seq libraries and transcriptome assembly.

Supplementary Appendix 3 REST software outputs to target transcripts’ relative expression assay.

## References

[B1] Ahammed G. J. Li X. Liu A. Chen S. (2020). Brassinosteroids in plant tolerance to abiotic stress. *J. Plant Growth Regul.* 39 1451–1464. 10.1007/s00344-020-10098-0

[B2] Amorim L. L. B. Ferreira-Neto J. R. C. Bezerra-Neto J. P. Pandolfi V. de Araujo F. T. da Silva Matos M. K. (2018). Cowpea and abiotic stresses: identification of reference genes for transcriptional profiling by qPCR. *Plant Methods* 14:88. 10.1186/s13007-018-0354-z 30337949 PMC6182843

[B3] Bailey T. L. Elkan C. (1994). Fitting a mixture model by expectation maximization to discover motifs in biopolymers. *Proc. Int. Conf. Intell. Syst. Mol. Biol.* 2 28–36. 7584402

[B4] Barna B. Király L. (2004). Host–pathogen relations: diseases caused by viruses, subviral organisms, and phytoplasmas. *Plant Toxicol.* 1, 533–568. 10.1201/9780203023884-13

[B5] Bastos E. A. Nascimento S. P. D. Silva E. M. D. Freire Filho F. R. Gomide R. L. (2011). Identification of cowpea genotypes for drought tolerance. *Rev. Ciênc. Agronôm.* 42 100–107. 10.1590/S1806-66902011000100013

[B6] Boller T. (2012). “Experimental evidence of a role for RLKS in innate immunity,” in *Receptor-like Kinases in Plants: From Development to Defense Signaling and Communication in Plants*, eds Tax F. Kemmerling B. (Berlin: Springer), 67–77. 10.1007/978-3-642-23044-8_4

[B7] Boukar O. Belko N. Chamarthi S. Togola A. Batieno J. Owusu E. (2016). Cowpea (*Vigna unguiculata*): genetics, genomics and breeding. *Plant Breed.* 138 415–424. 10.1111/pbr.12589

[B8] Bourgey M. Dali R. Eveleigh R. Chen K. C. Letourneau L. Fillon J. (2019). GenPipes: an open-source framework for distributed and scalable genomic analyses. *Gigascience* 8:giz037. 10.1093/gigascience/giz037 31185495 PMC6559338

[B9] Bustin S. A. Benes V. Garson J. A. Hellemans J. Huggett J. Kubista M. (2009). The MIQE guidelines: minimum information for publication of quantitative real-time PCR experiments. *Clin. Chem.* 55 611–622. 10.1373/clinchem.2008.112797 19246619

[B10] Cardoso M. J. Freire Filho F. R. Athayde-Sobrinho C. (1990). *BR 14-mulato: Nova Cultivar de Feijão Macassar Para o Estado do Piauí.* Teresina: Embrapa-UEPAE de Teresina, 4. (Comunicado Técnico, 48).

[B11] Cerbin S. Jiang N. (2018). Duplication of host genes by transposable elements. *Curr. Opin. Genet. Dev.* 49 63–69. 10.1016/j.gde.2018.03.005 29571044

[B12] Chinnusamy V. Zhu J. Zhu J.-K. (2007). Cold stress regulation of gene expression in plants. *Trends Plant Sci.* 12 444–451. 10.1016/j.tplants.2007.07.002 17855156

[B13] Ding X. Richter T. Chen M. Fujii H. Seo Y. S. Xie M. (2009). A rice kinase-protein interaction map. *Plant Physiol.* 149 1478–1492. 10.1104/pp.108.128298 19109415 PMC2649385

[B14] Fernando V. C. D. Schroeder D. F. (2016). “Role of ABA in *Arabidopsis* salt, drought, and desiccation tolerance,” in *Abiotic and Biotic Stress in Plants – Recent Advances and Future Perspectives*, eds Shanker A. K. Shanker C. (London: Intech), 10.5772/61957

[B15] Fujita Y. Nakashima K. Yoshida T. Katagiri T. Kidokoro S. Kanamori N. (2009). Three SnRK2 protein kinases are the main positive regulators of abscisic acid signaling in response to water stress in *Arabidopsis*. *Plant Cell Physiol.* 50 2123–2132. 10.1093/pcp/pcp147 19880399

[B16] Ganko E. W. Meyers B. C. Vision T. J. (2007). Divergence in expression between duplicated genes in *Arabidopsis*. *Mol. Biol. Evol.* 24 2298–2309. 10.1093/molbev/msm158 17670808

[B17] Gill R. A. Ali B. Yang S. Tong C. Islam F. Gill M. B. (2017). Reduced glutathione mediates pheno-ultrastructure, kinome and transportome in chromium-induced *Brassica napus* L. *Front. Plant Sci.* 8:2037. 10.3389/fpls.2017.02037 29312362 PMC5732361

[B18] Gupta S. Stamatoyannopoulos J. A. Bailey T. L. Noble W. S. (2007). Quantifying similarity between motifs. *Genome Biol.* 8:R24. 10.1186/gb-2007-8-2-r24 17324271 PMC1852410

[B19] Hedges S. B. (2002). The origin and evolution of model organisms. *Nat. Rev. Genet.* 3 838–849. 10.1038/nrg929 12415314

[B20] Hiller K. Grote A. Maneck M. Münch R. Jahn D. (2006). JVirGel 2.0: computational prediction of proteomes separated via two-dimensional gel electrophoresis under consideration of membrane and secreted proteins. *Bioinformatics* 22 2441–2443. 10.1093/bioinformatics/btl409 16870933

[B21] Hoagland D. R. Arnon D. I. (1950). *The Water-Culture Method for Growing Plants Without soil. Circular.* California Agricultural Experiment Station 347. Available online at: https://www.cabdirect.org/cabdirect/abstract/19500302257 (accessed April 24, 2020).

[B22] Jaggi M. (2018). “Recent advancement on map kinase cascade in biotic stress,” in *Molecular Aspects of Plant-Pathogen Interaction*, eds Singh A. Singh I. K. (Singapore: Springer), 139–158. 10.1007/978-981-10-7371-7_6

[B23] Kaló P. Seres A. Taylor S. A. Jakab J. Kevei Z. Kereszt A. (2004). Comparative mapping between *Medicago sativa* and *Pisum sativum*. *Mol. Genet. Genomics* 272 235–246. 10.1007/s00438-004-1055-z 15340836

[B24] Kang W.-H. Kim S. Lee H.-A. Choi D. Yeom S.-I. (2016). Genome-wide analysis of Dof transcription factors reveals functional characteristics during development and response to biotic stresses in pepper. *Sci. Rep.* 6 1–12. 10.1038/srep33332 27653666 PMC5032028

[B25] Kido E. A. Barbosa P. K. de A. Neto J. R. C. F. Pandolfi V. Houllou-Kido L. M. (2011). Identification of plant protein kinases in response to abiotic and biotic stresses using SuperSAGE. *Curr. Protein Pept. Sci.* 12 643–656. 10.2174/1389203711109070643 21827428

[B26] Kim D. Ntui V. O. Xiong L. (2016). Arabidopsis YAK1 regulates abscisic acid response and drought resistance. *FEBS Lett.* 590 2201–2209. 10.1002/1873-3468.12234 27264339

[B27] Korver R. A. Koevoets I. T. Testerink C. (2018). Out of shape during stress: a key role for auxin. *Trends Plant Sci* 23 783–793. 10.1016/j.tplants.2018.05.011 29914722 PMC6121082

[B28] Kumar S. Stecher G. Tamura K. (2016). MEGA7: molecular evolutionary genetics analysis version 7.0 for bigger datasets. *Mol. Biol. Evol.* 33 1870–1874. 10.1093/molbev/msw054 27004904 PMC8210823

[B29] Larkin M. A. Blackshields G. Brown N. P. Chenna R. McGettigan P. A. McWilliam H. (2007). Clustal W and Clustal X version 2.0. *Bioinformatics* 23 2947–2948. 10.1093/bioinformatics/btm404 17846036

[B30] Lehti-Shiu M. D. Shiu S.-H. (2012). Diversity, classification and function of the plant protein kinase superfamily. *Philos. Trans. R. Soc. B* 367 2619–2639. 10.1098/rstb.2012.0003 22889912 PMC3415837

[B31] Lehti-Shiu M. D. Zou C. Shiu S.-H. (2011). “Origin, diversity, expansion history, and functional evolution of the plant receptor-like kinase/pelle family,” in *Receptor-like Kinases in Plants: From Development to Defense Signaling and Communication in Plants*, eds Tax F. Kemmerling B. (Berlin: Springer), 1–22. 10.1007/978-3-642-23044-8_1

[B32] Lehti-Shiu M. D. Zou C. Hanada K. Shiu S.-H. (2009). Evolutionary history and stress regulation of plant receptor-like kinase/pelle genes. *Plant Physiol.* 150 12–26. 10.1104/pp.108.134353 19321712 PMC2675737

[B33] Lei W. Ni D. Wang Y. Shao J. Wang X. Yang D. (2016). Intraspecific and heteroplasmic variations, gene losses and inversions in the chloroplast genome of *Astragalus membranaceus*. *Sci. Rep.* 6:21669. 10.1038/srep21669 26899134 PMC4761949

[B34] Liu H. Qu W. Zhu K. Cheng Z.-M. (2020). The wild strawberry kinome: identification, classification and transcript profiling of protein kinases during development and in response to gray mold infection. *BMC Genomics* 21:635. 10.1186/s12864-020-07053-4 32928117 PMC7490889

[B35] Liu J. Chen N. Grant J. N. Cheng Z.-M. M. Stewart C. N. Hewezi T. (2015). Soybean kinome: functional classification and gene expression patterns. *J. Exp. Bot.* 66 1919–1934. 10.1093/jxb/eru537 25614662 PMC4378628

[B36] Lonardi S. Muñoz-Amatriaín M. Liang Q. Shu S. Wanamaker S. I Lo S. (2019). The genome of cowpea (*Vigna unguiculata* [L.] Walp.). *Plant J.* 98 767–782. 10.1111/tpj.14349 31017340 PMC6852540

[B37] McCready K. Spencer V. Kim M. (2020). The importance of TOR kinase in plant development. *Front. Plant Sci.* 11:16. 10.3389/fpls.2020.00016 32117365 PMC7012898

[B38] Minkoff B. B. Makino S. Haruta M. Beebe E. T. Wrobel R. L. Fox B. G. (2017). A cell-free method for expressing and reconstituting membrane proteins enables functional characterization of the plant receptor-like protein kinase FERONIA. *J. Biol. Chem.* 292 5932–5942. 10.1074/jbc.M116.761981 28235802 PMC5392584

[B39] Oliveira C. R. R. de Freire Filho F. R. Nogueira M. do S. da R. Barros G. B. (2012). *Reação de Genótipos de Feijão-caupi Revela Resistência às Coinfecções pelo Cucumber Mosaic virus, Cowpea aphid-borne Mosaic Virus e Cowpea Severe Mosaic Virus.* Available online at: http://www.alice.cnptia.embrapa.br/handle/doc/940785 (accessed October 12, 2020).

[B40] Osakabe Y. Yamaguchi-Shinozaki K. Shinozaki K. Tran L.-S. P. (2013). Sensing the environment: key roles of membrane-localized kinases in plant perception and response to abiotic stress. *J. Exp. Bot.* 64 445–458. 10.1093/jxb/ers354 23307915

[B41] Ostlund G. Schmitt T. Forslund K. Köstler T. Messina D. N. Roopra S. (2010). InParanoid 7: new algorithms and tools for eukaryotic orthology analysis. *Nucleic Acids Res.* 38 D196–D203. 10.1093/nar/gkp931 19892828 PMC2808972

[B42] Pfaffl M. W. Horgan G. W. Dempfle L. (2002). Relative expression software tool (REST) for group-wise comparison and statistical analysis of relative expression results in real-time PCR. *Nucleic Acids Res.* 30:e36. 10.1093/nar/30.9.e36 11972351 PMC113859

[B43] Prasch C. M. Sonnewald U. (2013). Simultaneous application of heat, drought, and virus to *Arabidopsis* plants reveals significant shifts in signaling networks. *Plant Physiol.* 162 1849–1866. 10.1104/pp.113.221044 23753177 PMC3729766

[B44] Rauch J. Volinsky N. Romano D. Kolch W. (2011). The secret life of kinases: functions beyond catalysis. *Cell Commun. Signal.* 9:23. 10.1186/1478-811X-9-23 22035226 PMC3215182

[B45] Ritsema T. Joore J. van Workum W. Pieterse C. M. (2007). Kinome profiling of *Arabidopsis* using arrays of kinase consensus substrates. *Plant Methods* 3:3. 10.1186/1746-4811-3-3 17295910 PMC1803769

[B46] Robinson M. D. McCarthy D. J. Smyth G. K. (2010). edgeR: a bioconductor package for differential expression analysis of digital gene expression data. *Bioinformatics* 26 139–140. 10.1093/bioinformatics/btp616 19910308 PMC2796818

[B47] Rocha M. M. Lima J. A. A. Freire Filho F. R. R. Lima V. C. V. (1996). *Resistencia de Caupi de Tegumento Branco a Algumas Estirpes de Comovirus, Potyvirus e Cucumovirus.* Available online at: http://www.alice.cnptia.embrapa.br/handle/doc/53796 (accessed October 12, 2020).

[B48] Rodrigues E. V. Damasceno-Silva K. J. Rocha M. de M. Bastos E. A. Teodoro P. E. (2017). Selection of cowpea populations tolerant to water deficit by selection index. *Rev. Ciênc. Agron.* 48 889–896. 10.5935/1806-6690.20170105

[B49] Rodrigues F. A. Marcolino-Gomes J. de Fátima Corrêa Carvalho J. do Nascimento L. C. Neumaier N. Farias J. R. B. (2012). Subtractive libraries for prospecting differentially expressed genes in the soybean under water deficit. *Genet. Mol. Biol.* 35 304–314. 10.1590/S1415-47572012000200011 22802715 PMC3392882

[B50] Saitou N. Nei M. (1987). The neighbor-joining method: a new method for reconstructing phylogenetic trees. *Mol. Biol. Evol.* 4 406–425. 10.1093/oxfordjournals.molbev.a040454 3447015

[B51] Shiu S.-H. Bleecker A. B. (2001). Receptor-like kinases from *Arabidopsis* form a monophyletic gene family related to animal receptor kinases. *Proc. Natl. Acad. Sci. U.S.A.* 98 10763–10768. 10.1073/pnas.181141598 11526204 PMC58549

[B52] Singh D. K. Calviño M. Brauer E. K. Fernandez-Pozo N. Strickler S. Yalamanchili R. (2013). The tomato kinome and the tomato kinase library ORFeome: novel resources for the study of kinases and signal transduction in tomato and *Solanaceae* species. *MPMI* 27 7–17. 10.1094/MPMI-08-13-0218-TA 24047240

[B53] Sinha R. Irulappan V. Mohan-Raju B. Suganthi A. Senthil-Kumar M. (2019). Impact of drought stress on simultaneously occurring pathogen infection in field-grown chickpea. *Sci. Rep.* 9:5577. 10.1038/s41598-019-41463-z 30944350 PMC6447570

[B54] Sonnhammer E. L. L. Östlund G. (2015). InParanoid 8: orthology analysis between 273 proteomes, mostly eukaryotic. *Nucleic Acids Res.* 43 D234–D239. 10.1093/nar/gku1203 25429972 PMC4383983

[B55] Stahl Y. Simon R. (2012). “Receptor kinases in plant meristem development,” in *Receptor-like Kinases in Plants: From Development to Defense. Signaling and Communication in Plants*, eds Tax F. Kemmerling B. (Berlin: Springer), 23–39. 10.1007/978-3-642-23044-8_2

[B56] Torres G. A. M. Gimenes M. A. de Rosa V. E. Jr. Quecini V. (2007). Identifying water stress-response mechanisms in citrus by in silico transcriptome analysis. *Genet. Mol. Biol.* 30 888–905. 10.1590/S1415-47572007000500018

[B57] Umezawa T. Yoshida R. Maruyama K. Yamaguchi-Shinozaki K. Shinozaki K. (2004). SRK2C, a SNF1-related protein kinase 2, improves drought tolerance by controlling stress-responsive gene expression in *Arabidopsis thaliana*. *Proc. Natl. Acad. Sci. U.S.A.* 101 17306–17311. 10.1073/pnas.0407758101 15561775 PMC535404

[B58] Wang K. Ding Y. Cai C. Chen Z. Zhu C. (2019). The role of C2H2 zinc finger proteins in plant responses to abiotic stresses. *Physiol. Plant* 165 690–700. 10.1111/ppl.12728 29572849

[B59] Wang M. Zheng Q. Shen Q. Guo S. (2013). The critical role of potassium in plant stress response. *Int. J. Mol. Sci.* 14 7370–7390. 10.3390/ijms14047370 23549270 PMC3645691

[B60] Wang Y. Ficklin S. P. Wang X. Feltus F. A. Paterson A. H. (2016). Large-scale gene relocations following an ancient genome triplication associated with the diversification of core eudicots. *PLoS One* 11:e0155637. 10.1371/journal.pone.0155637 27195960 PMC4873151

[B61] Wang Y. Tang H. DeBarry J. D. Tan X. Li J. Wang X. (2012). MCScanX: a toolkit for detection and evolutionary analysis of gene synteny and collinearity. *Nucleic Acids Res.* 40:e49. 10.1093/nar/gkr1293 22217600 PMC3326336

[B62] Wei K. Wang Y. Xie D. (2014). Identification and expression profile analysis of the protein kinase gene superfamily in maize development. *Mol. Breed.* 33 155–172. 10.1007/s11032-013-9941-x

[B63] Yan J. Li G. Guo X. Li Y. Cao X. (2018). Genome-wide classification, evolutionary analysis and gene expression patterns of the kinome in *Gossypium*. *PLoS One* 13:e0197392. 10.1371/journal.pone.0197392 29768506 PMC5955557

[B64] Ye Y. Ding Y. Jiang Q. Wang F. Sun J. Zhu C. (2017). The role of receptor-like protein kinases (RLKs) in abiotic stress response in plants. *Plant Cell Rep.* 36 235–242. 10.1007/s00299-016-2084-x 27933379

[B65] Yu C.-S. Chen Y.-C. Lu C.-H. Hwang J.-K. (2006). Prediction of protein subcellular localization. *Proteins* 64 643–651. 10.1002/prot.21018 16752418

[B66] Zheng Y. Jiao C. Sun H. Rosli H. G. Pombo M. A. Zhang P. (2016). iTAK: a program for genome-wide prediction and classification of plant transcription factors, transcriptional regulators, and protein kinases. *Mol. Plant* 9 1667–1670. 10.1016/j.molp.2016.09.014 27717919

[B67] Zhu J.-K. (2016). Abiotic stress signaling and responses in plants. *Cell* 167 313–324. 10.1016/j.cell.2016.08.029 27716505 PMC5104190

[B68] Zhu K. Liu H. Chen X. Cheng Q. Cheng Z.-M. (2018a). The kinome of pineapple: catalog and insights into functions in crassulacean acid metabolism plants. *BMC Plant Biol.* 18:199. 10.1186/s12870-018-1389-z 30227850 PMC6145126

[B69] Zhu K. Wang X. Liu J. Tang J. Cheng Q. Chen J.-G. (2018b). The grapevine kinome: annotation, classification and expression patterns in developmental processes and stress responses. *Hortic. Res.* 5 1–16. 10.1038/s41438-018-0027-0 29619230 PMC5878832

[B70] Zulawski M. Schulze G. Braginets R. Hartmann S. Schulze W. X. (2014). The *Arabidopsis kinome*: phylogeny and evolutionary insights into functional diversification. *BMC Genomics* 15:548. 10.1186/1471-2164-15-548 24984858 PMC4112214

[B71] Zuniga-Leon E. Carrasco-Navarro U. Fierro F. (2018). NeVOmics: an enrichment tool for gene ontology and functional network analysis and visualization of data from OMICs technologies. *Genes (Basel)* 9:569. 10.3390/genes9120569 30477135 PMC6316660

