# Supplementary figures and images for: The Cowpea Kinome: Genomic and Transcriptomic Analysis Under Biotic and Abiotic Stresses
Source: Front Plant Sci. 2021 Jun 14;12:667013. doi: 10.3389/fpls.2021.667013 (PMC8238008; doi:10.3389/fpls.2021.667013)

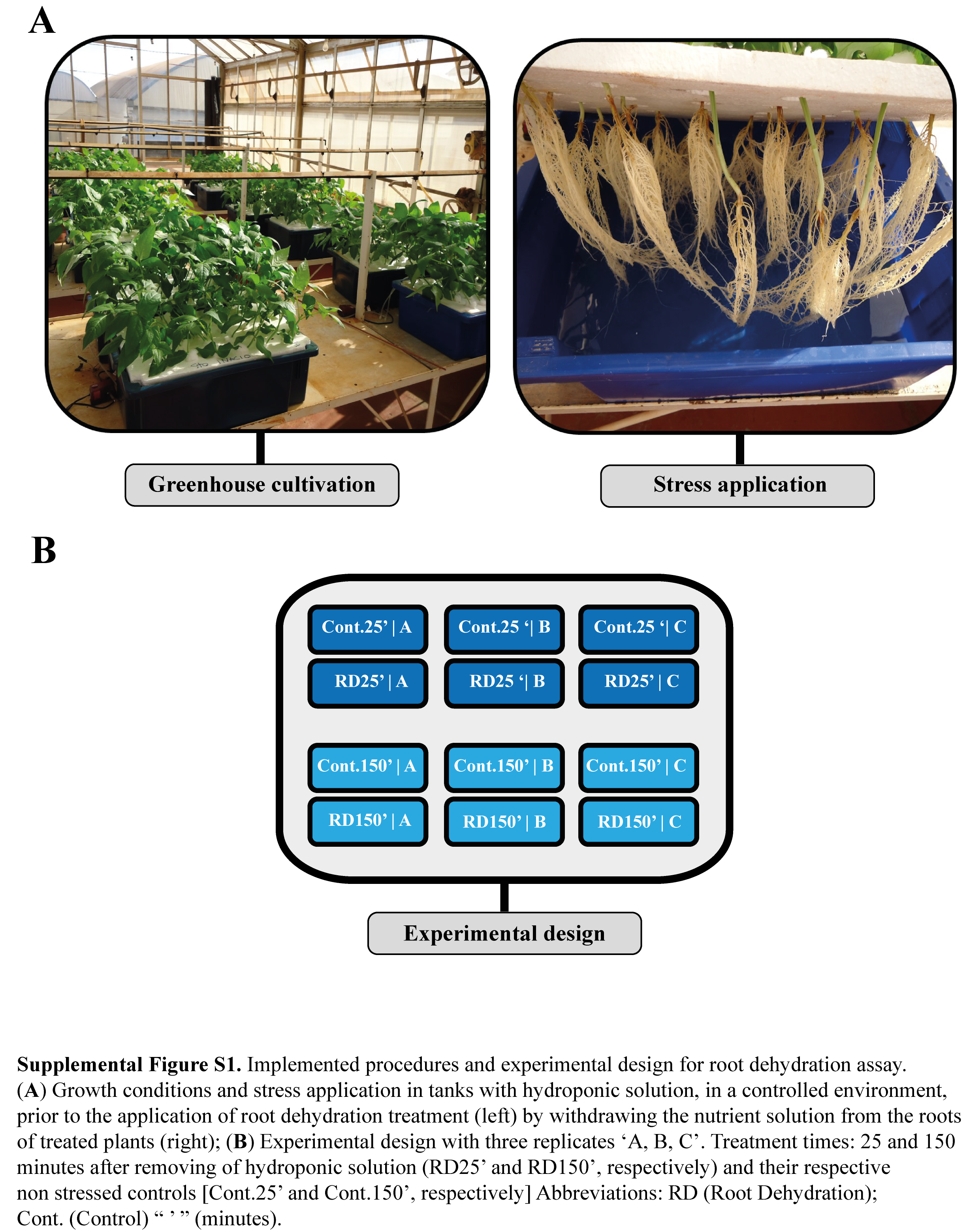

Supplement: Supplementary Figure 1 — Implemented procedures and experimental design for root dehydration assay. (A) Growth conditions and stress application in tanks with hydroponic solution, in a controlled environment, prior to the application of root dehydration treatment (left) by withdrawing the nutrient solution from the roots of the treated plants (right). (B) Experimental design with three replicates “A, B, C.” Treatment time: 25 and 150 min after removal of hydroponic solution (RD25’ and RD150’, respectively) and their respective non-stressed controls (Cont.25’ and Cont.150’, respectively). RD, root dehydration; Cont., control;’, minutes. [file Image_1.TIF]

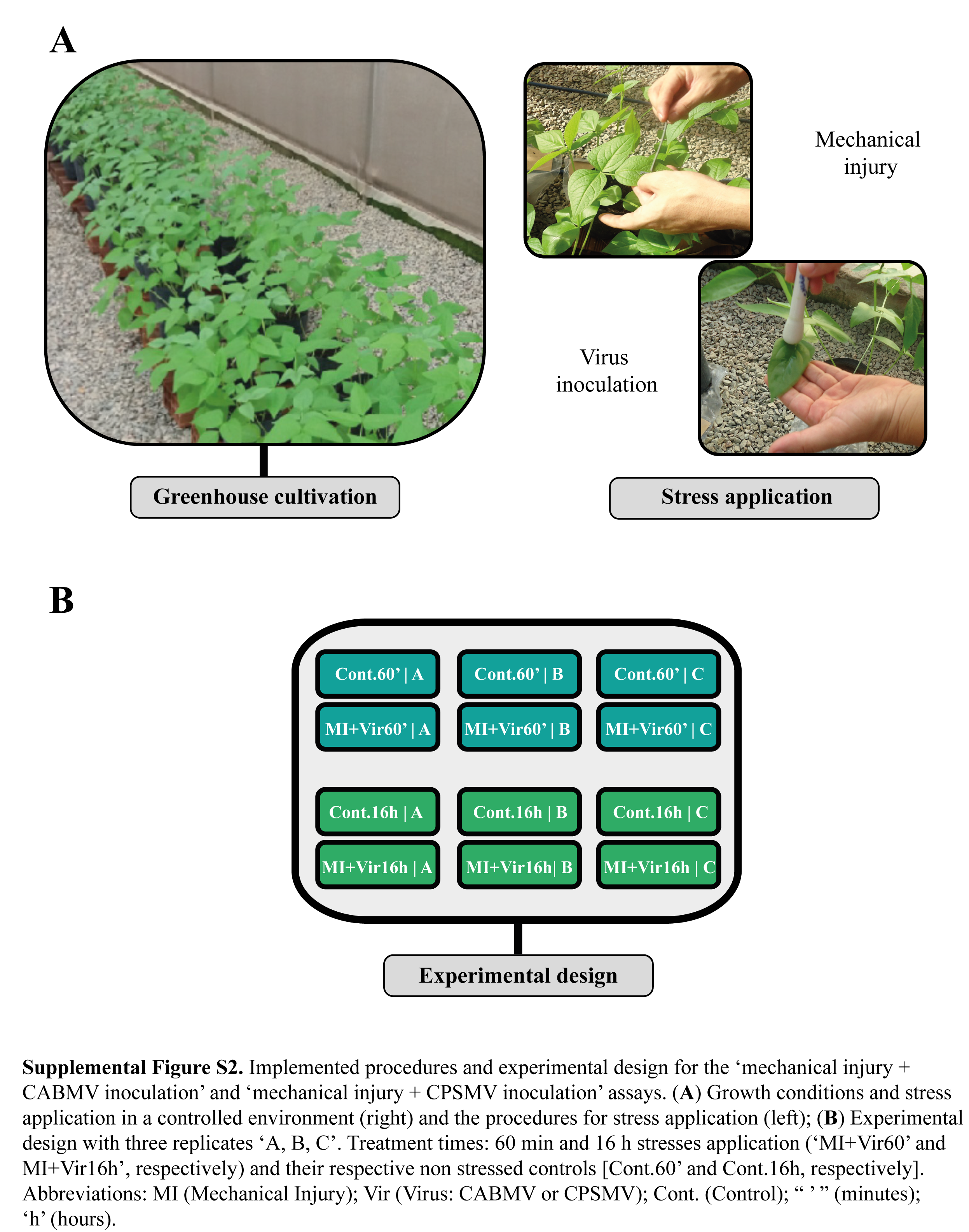

Supplement: Supplementary Figure 2 — Implemented procedures and experimental design for the ‘mechanical injury + CABMV inoculation’ and ‘mechanical injury + CPSMV inoculation’ assays. (A) Growth conditions and stress application in a controlled environment (right) and the procedures for stress application (left). (B) Experimental design with three replicates “A, B, C.” Treatment time: 60 min and 16 h after stress application (‘MI + Vir60’ and ‘MI + Vir16h,’ respectively) and their respective non-stressed controls (Cont.60’ and Cont.16h, respectively). MI, mechanical injury; Vir, virus (CABMV or CPSMV); Cont., control;’, minutes; h, hours. [file Image_2.TIF]

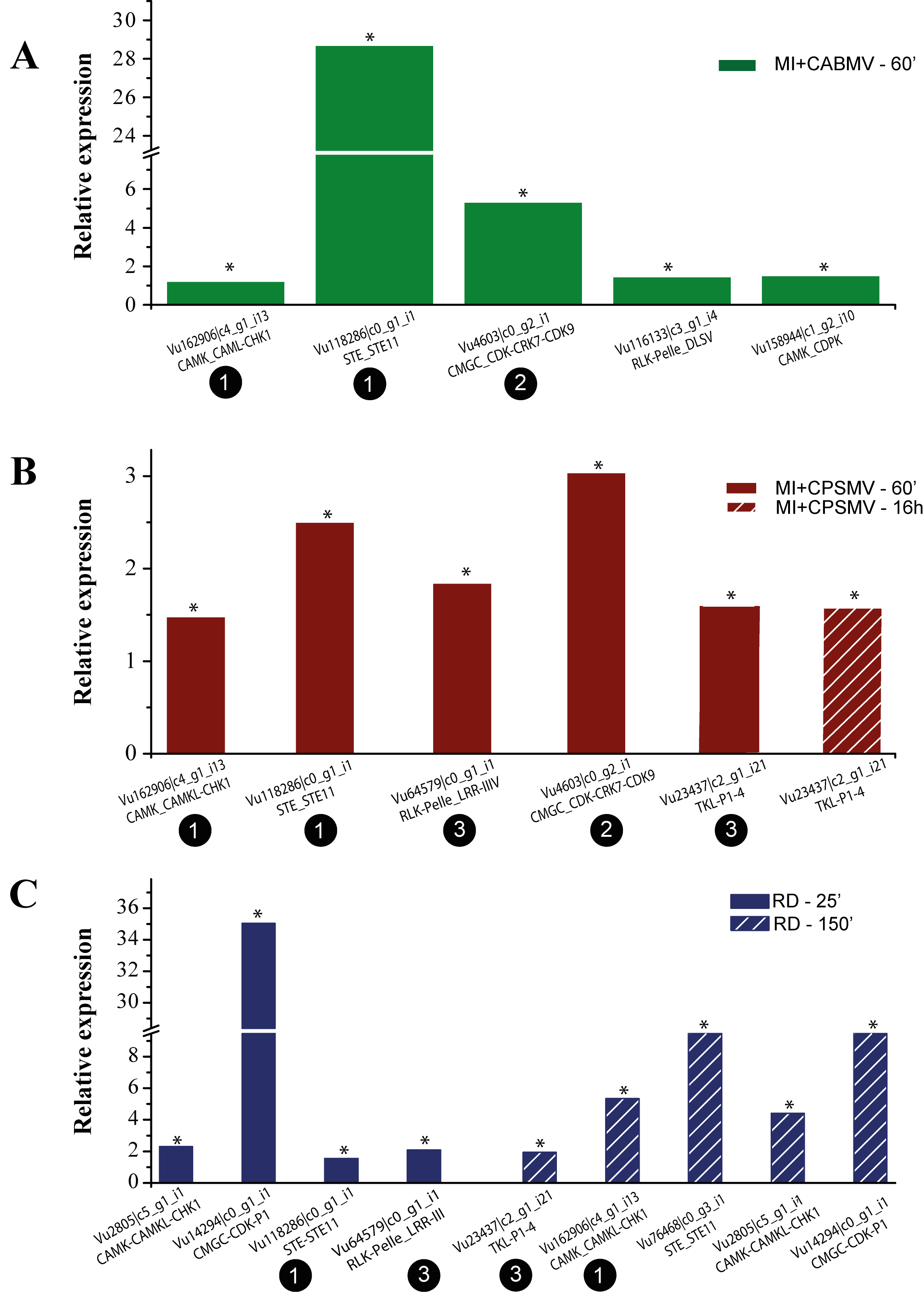

Supplement: Supplementary Figure 3 — Neighbor-joining distance tree illustrating VuPK phenetic relationships. Values at nodes correspond to the bootstrap values. UNK (unknown) refers to VuPKs whose phenetic grouping did not occur with their peers (based on iTAK classification). [file Image_3.TIF]
