# Supplementary material for: The Cowpea Kinome: Genomic and Transcriptomic Analysis Under Biotic and Abiotic Stresses
Source: Front Plant Sci. 2021 Jun 14;12:667013. doi: 10.3389/fpls.2021.667013 (PMC8238008; doi:10.3389/fpls.2021.667013)

Supplemental Appendix S1. Melting curves for target transcripts used in the present study.

Transcript CpGC ID: Vu2805c5_g1_i1; **Treatment Time:** 25'; **Stress:** Root dehydration; **Kinase family:** CAMK_CAMKL-CHK1

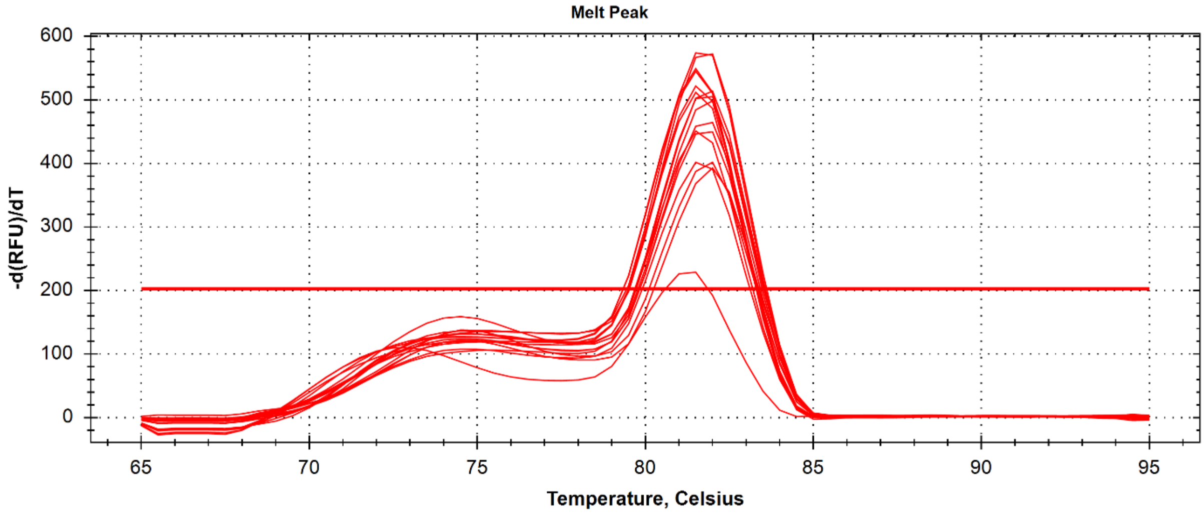

Transcript CpGC ID: Vu2805c5_g1_i1; **Treatment Time:** 150'; **Stress:** Root dehydration; **Kinase family:** CAMK_CAMKL-CHK1

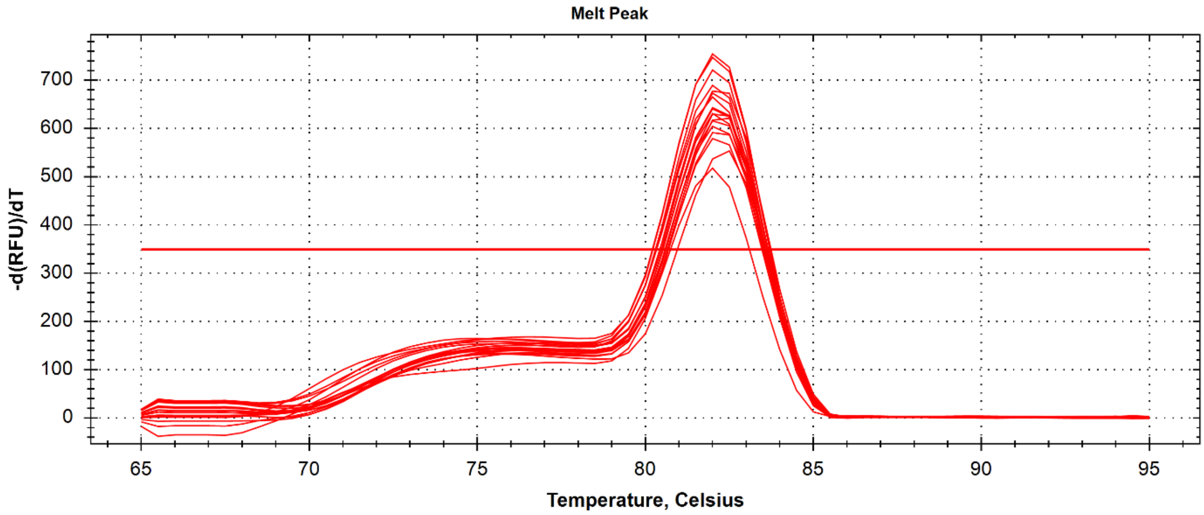

Transcript CpGC ID: Vu4603c0_g2_i1; **Treatment Time:** 60’; **Stress:** CABMV+MI; **Kinase family:** CMGC_CDK-CRK7-CDK9

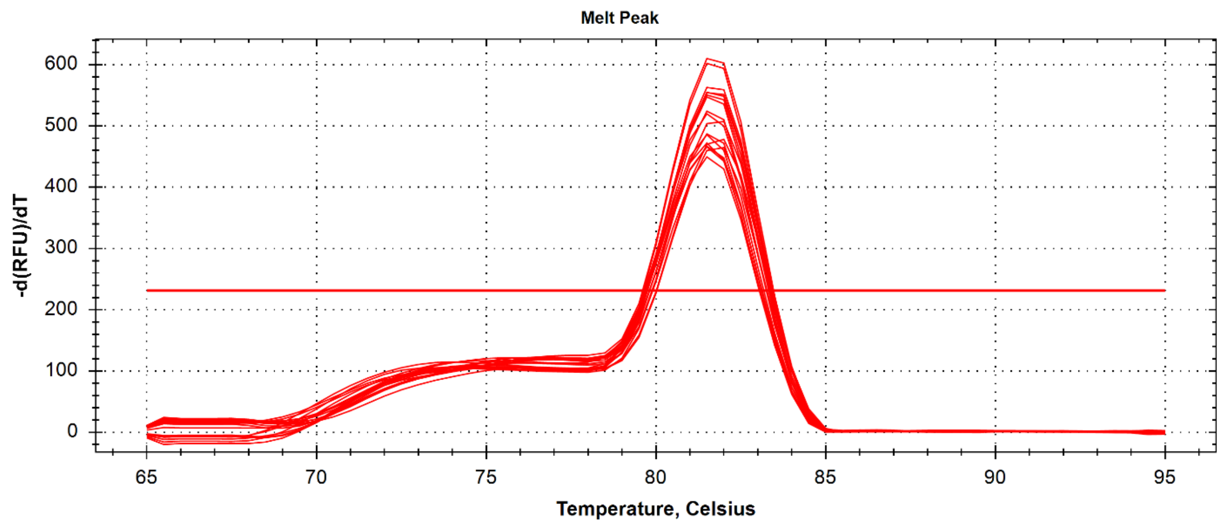

Transcript CpGC ID: Vu4603c0_g2_i1; **Treatment Time:** 60’; **Stress:** CPSMV+MI; **Kinase family:** CMGC_CDK-CRK7-CDK9

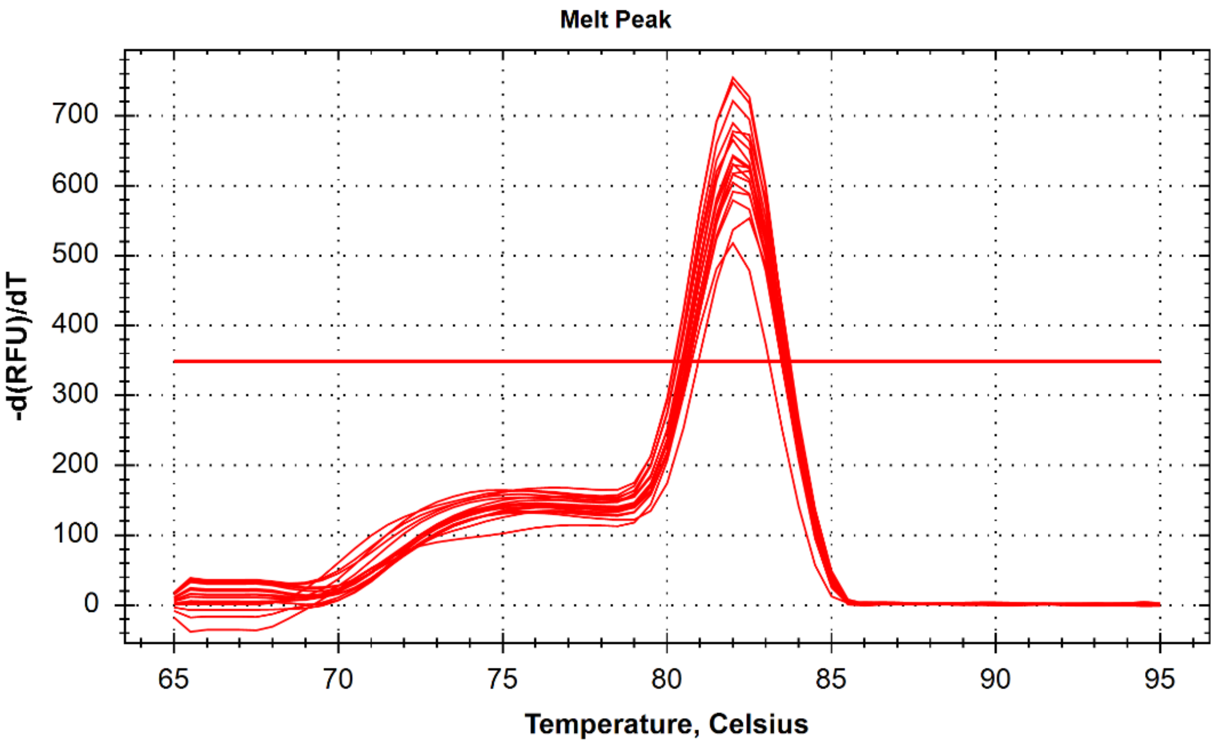

Transcript CpGC ID: Vu14294c0_g1_i1; **Treatment Time:** 25'; **Stress:** Root dehydration; **Kinase family:** CMGC_CDK-Pl

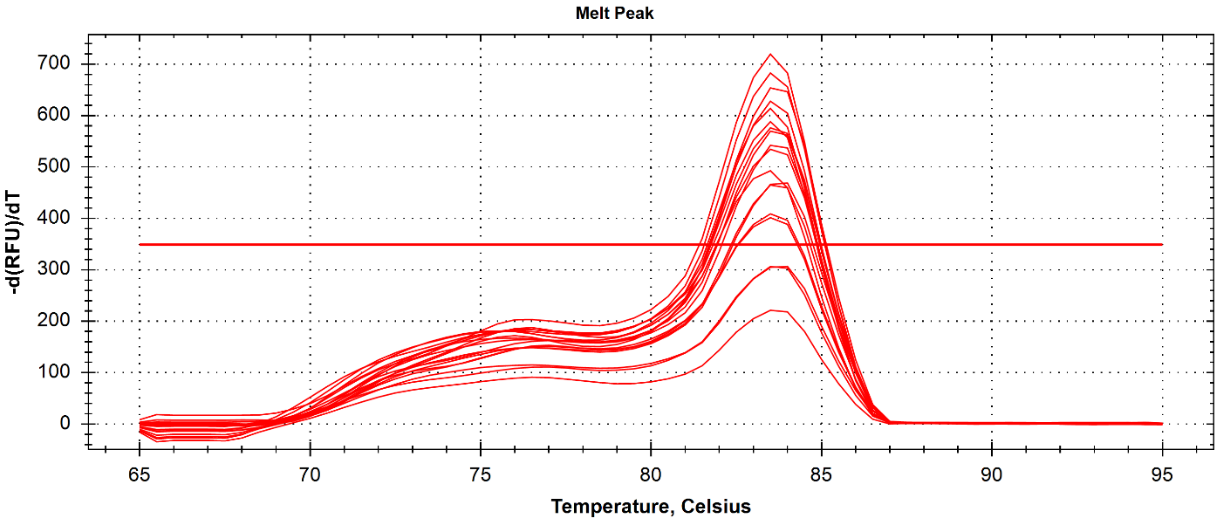

Transcript CpGC ID: Vu14294c0_g1_i1; **Treatment Time:** 150'; **Stress:** Root dehydration; **Kinase family:** CMGC_CDK-Pl

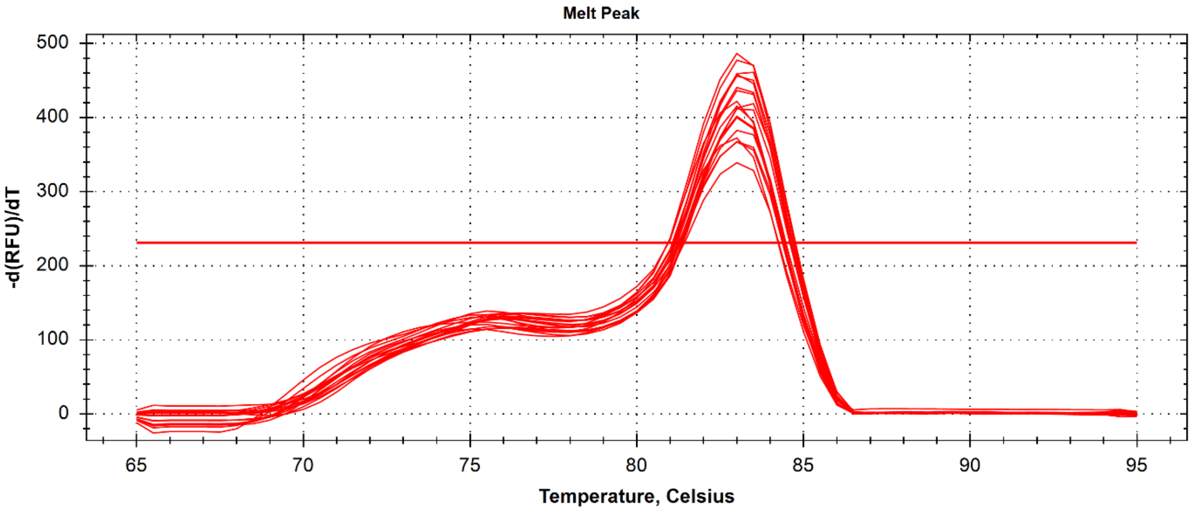

Transcript CpGC ID: Vu23437c2_g1_i21; **Treatment Time:** 16h; **Stress:** CPSMV+MI; **Kinase family:** TKL-Pl-4

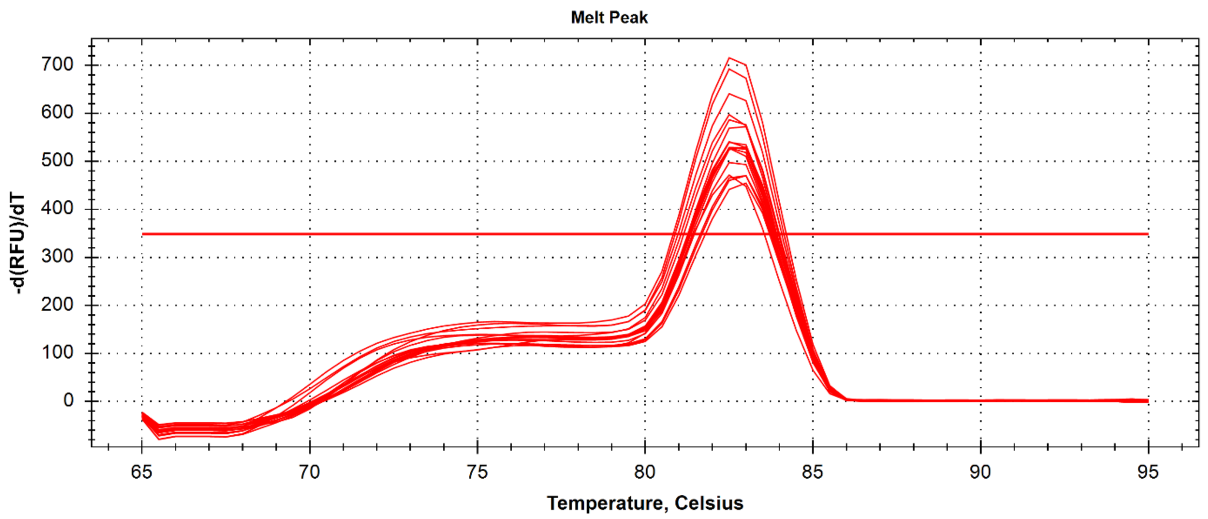

Transcript CpGC ID: Vu23437c2_g1_i21; **Treatment Time:** 60’; **Stress:** CPSMV+MI; **Kinase family:** TKL-Pl-4

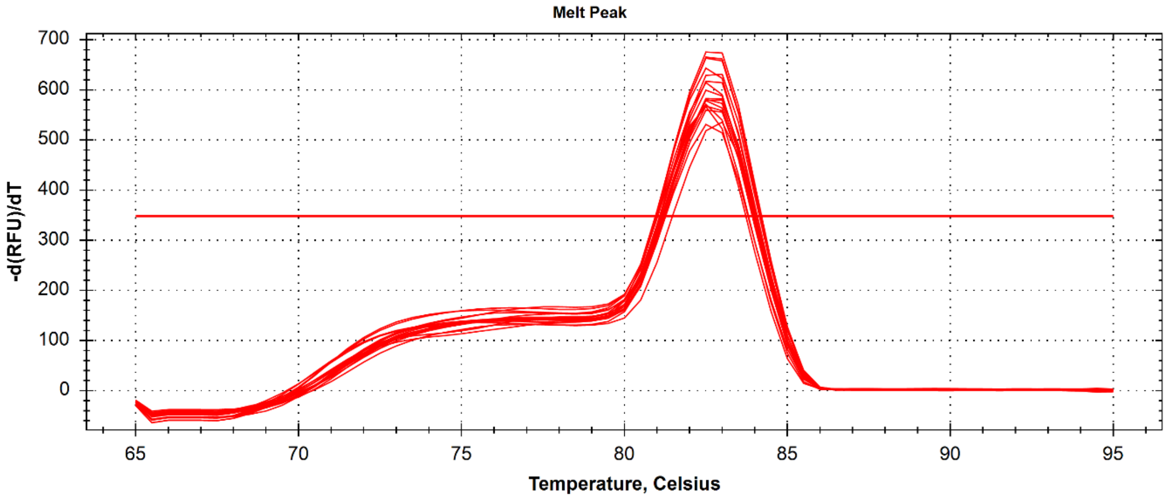

Transcript CpGC ID: Vu23437c2_g1_i21; **Treatment Time:** 150'; **Stress:** Root dehydration; **Kinase family:** TKL-Pl-4

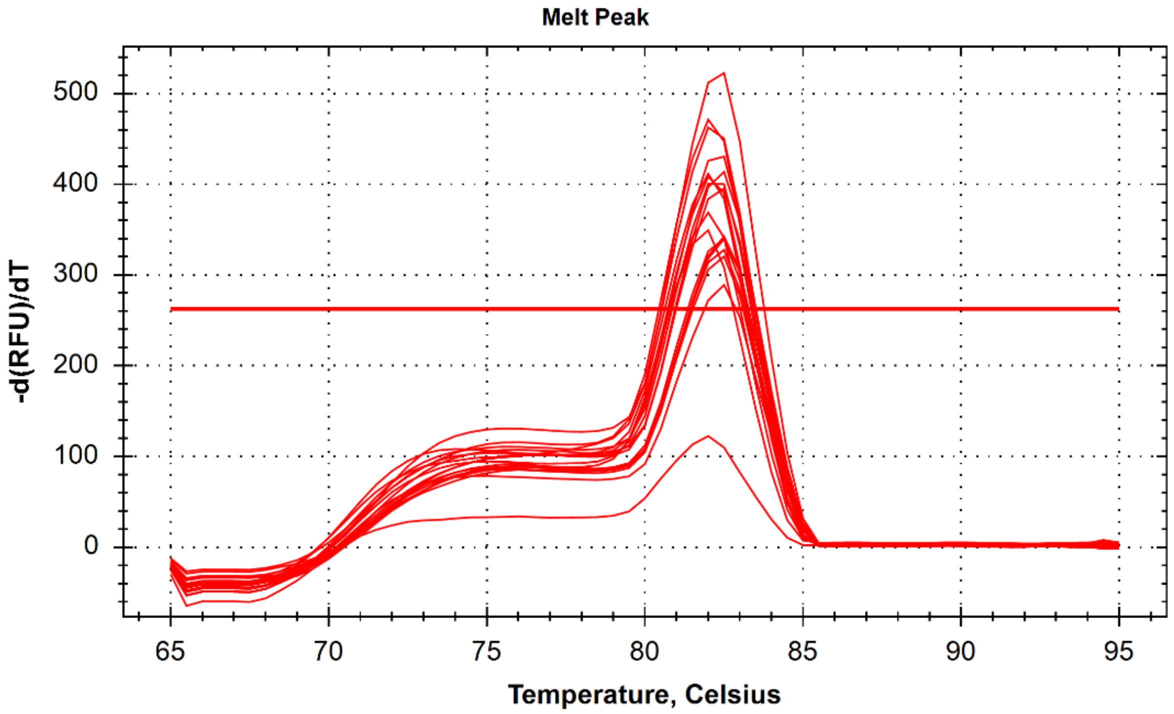

Transcript CpGC ID: Vu64579c0_g1_i1; **Treatment Time:** 60'; **Stress:** CPSMV+MI; **Kinase family:** RLK-Pelle_LRR-III

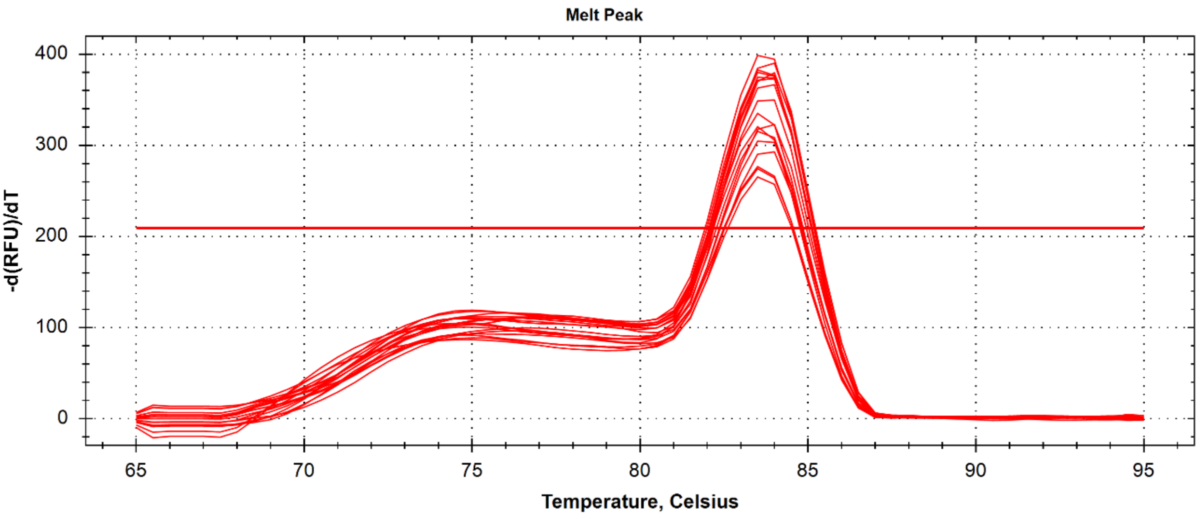

Transcript CpGC ID: Vu64579c0_g1_i1; **Treatment Time:** RD25'; **Stress:** Root dehydration; **Kinase family:** RLK-Pelle_LRR-III

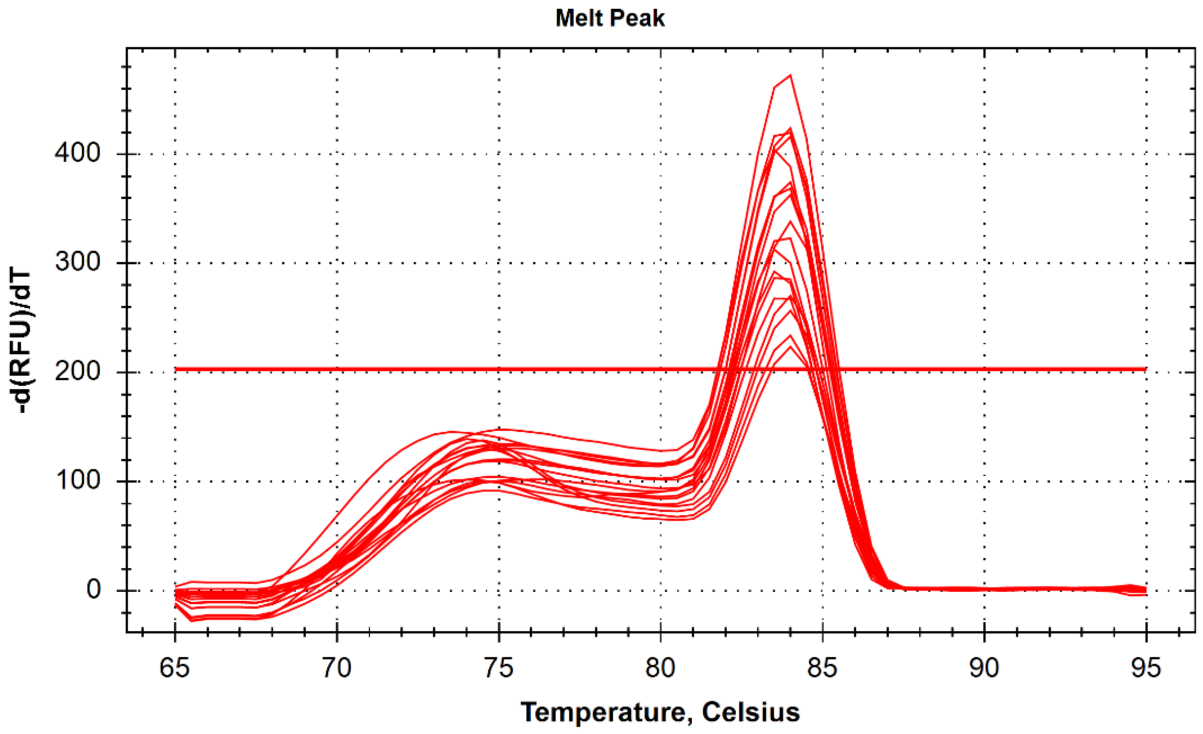

Transcript CpGC ID: Vu76468c0_g3_i1; **Treatment Time:** 150'; **Stress:** Root dehydration; **Kinase family:** STE_STE11

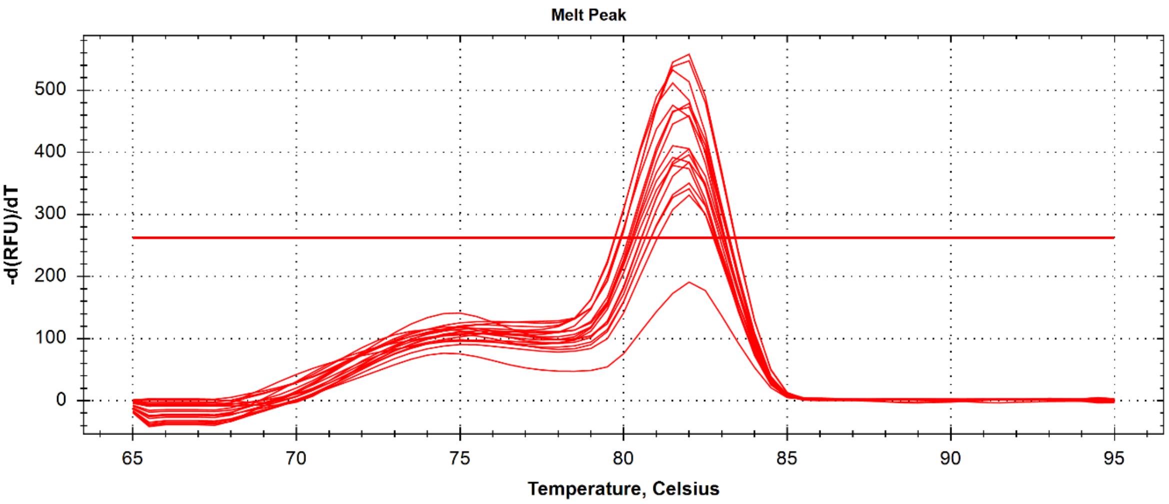

Transcript CpGC ID: Vu116133c3_g1_i4; **Treatment Time:** 60'; **Stress:** CABMV+MI; **Kinase family:** RLK-Pelle_DLSV

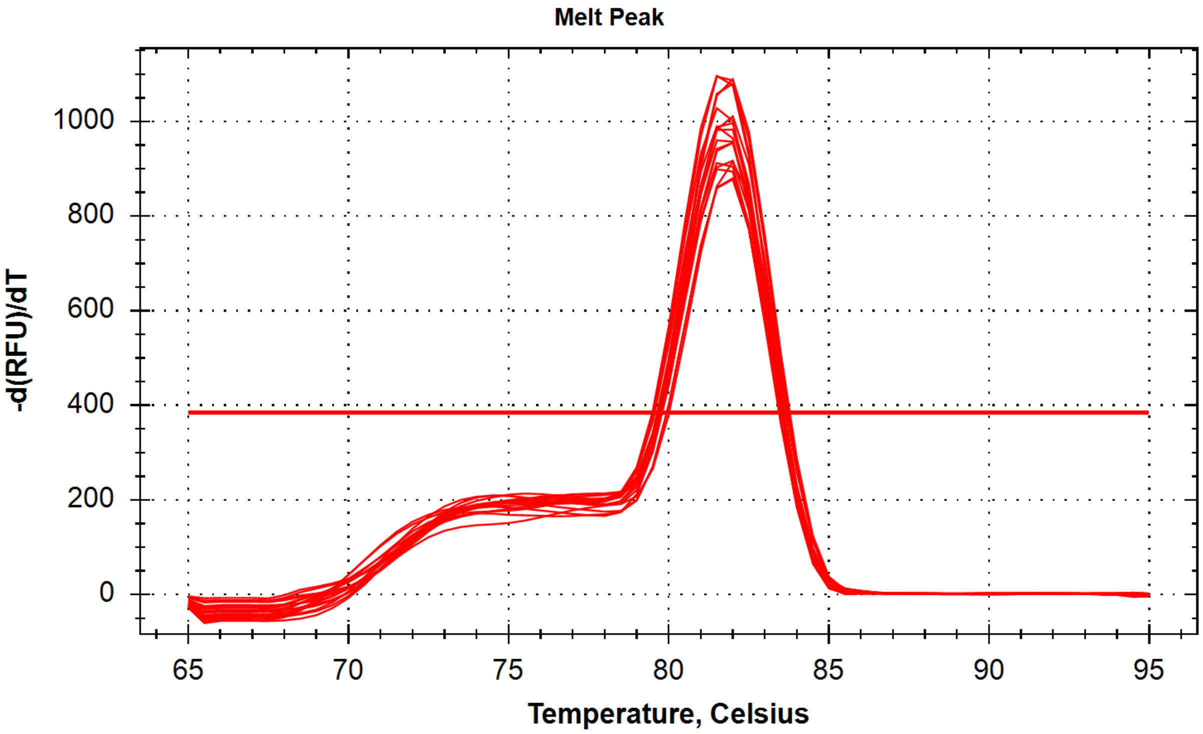

Transcript CpGC ID: Vu118286c0_g1_i1; **Treatment Time:** 60'; **Stress:** CABMV+MI; **Kinase family:** STE_STE11

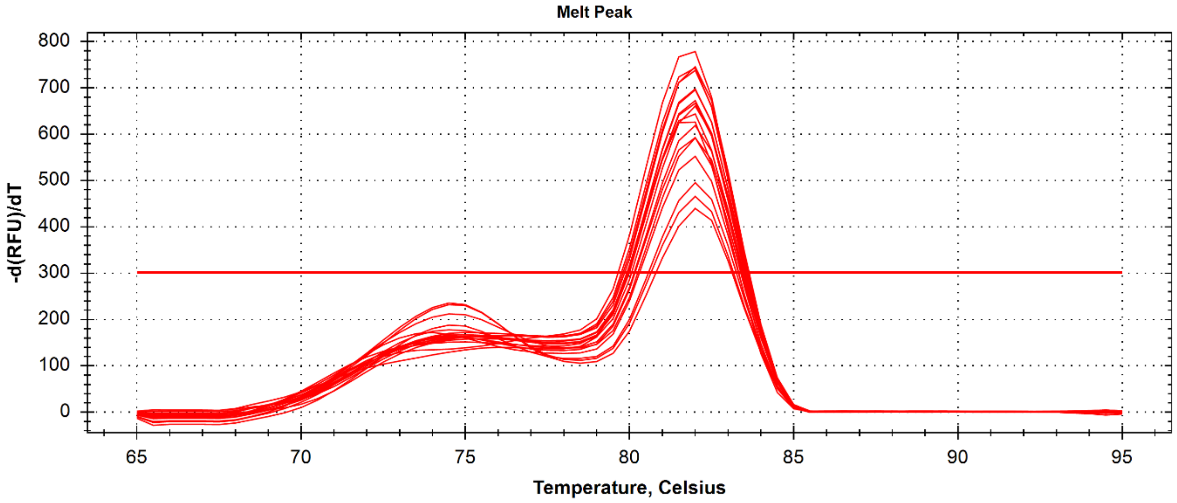

Transcript CpGC ID: Vu118286c0_g1_i1; **Treatment Time:** 60'; **Stress:** CPSMV+MI; **Kinase family:** STE_STE11

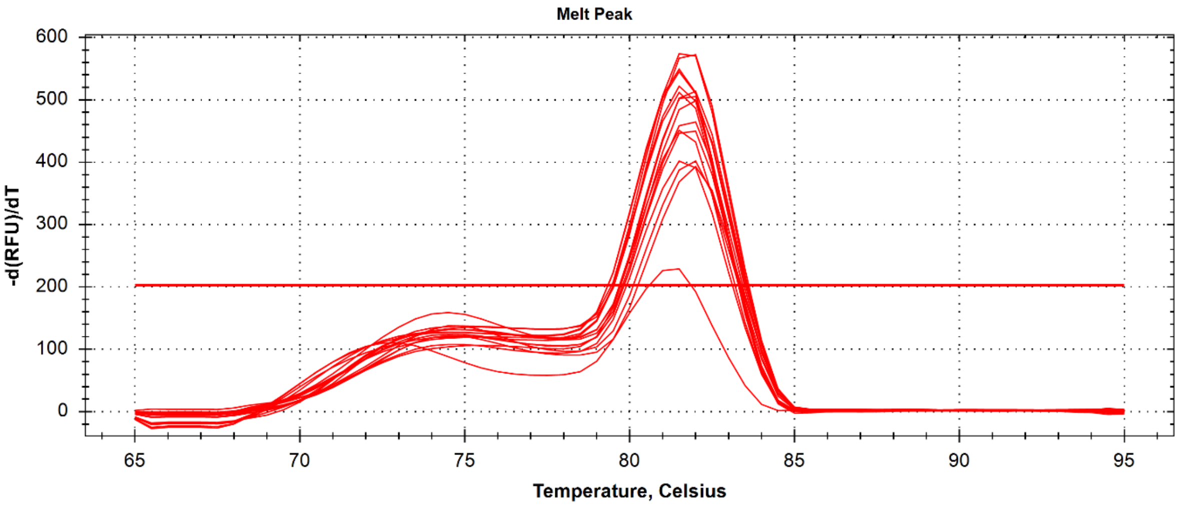

Transcript CpGC ID: Vu118286c0_g1_i1; **Treatment Time:** 25'; **Stress:** Root dehydration; **Kinase family:** STE_STE11

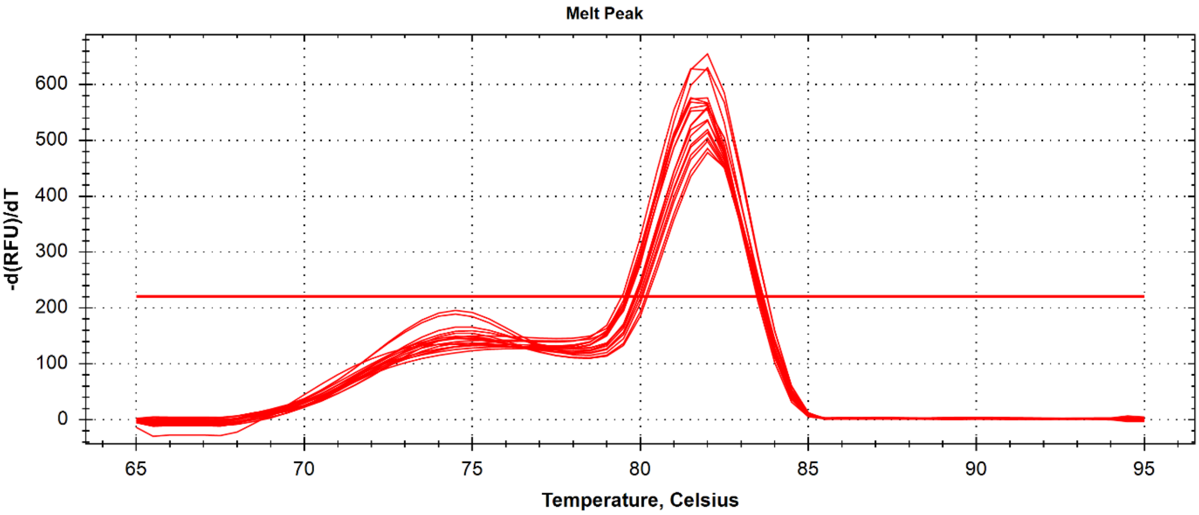

Transcript CpGC ID: Vu158944c1_g2_i10; **Treatment Time:** 60'; **Stress:** CABMV+MI; **Kinase family:** CAMK_CDPK

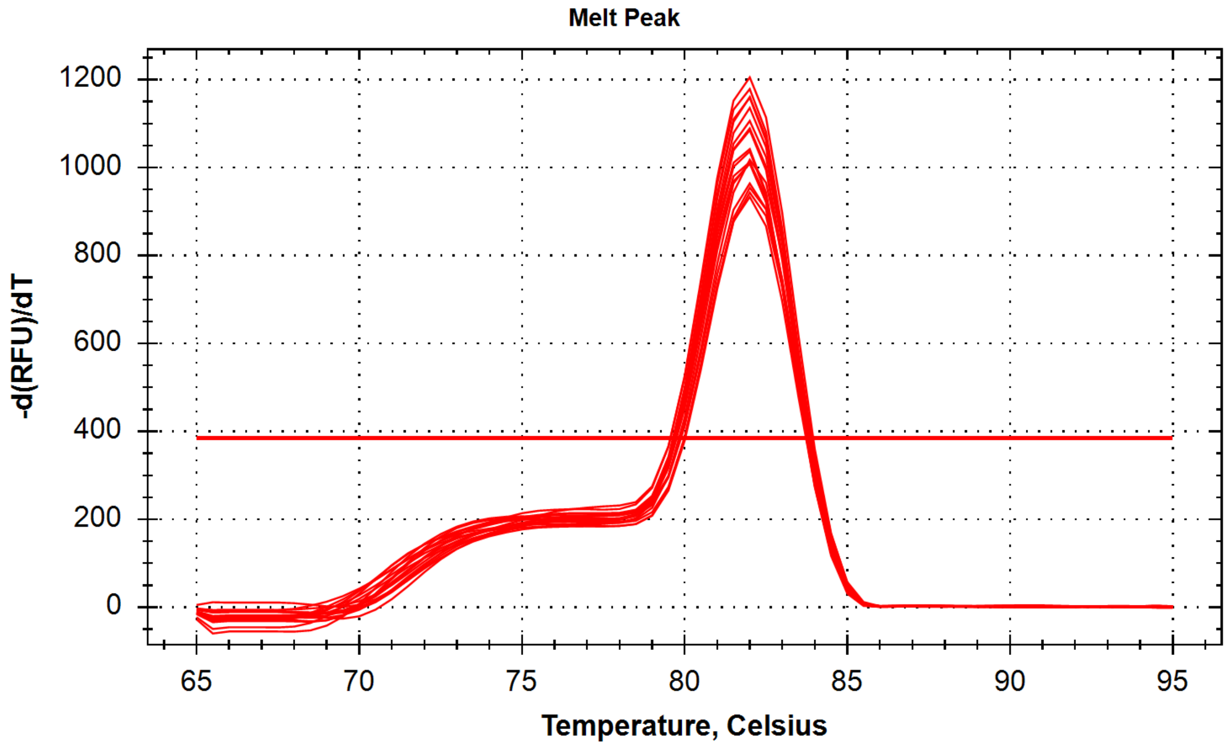

Transcript CpGC ID: Vu162906c4_g1_i13; **Treatment Time:** 60'; **Stress:** CABMV+MI; **Kinase family:** CAMK_CAMKL-CHK1

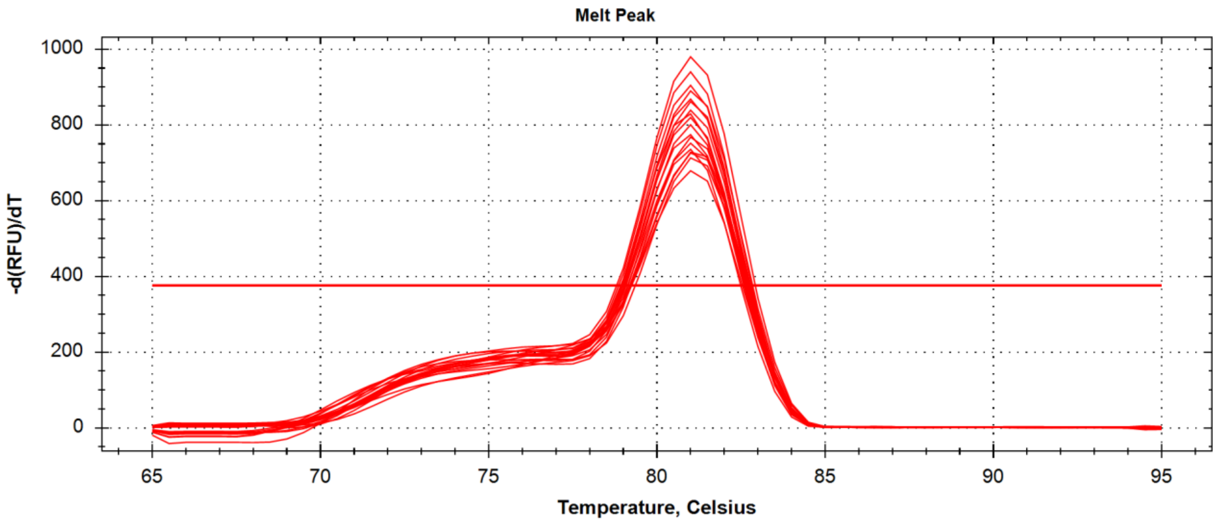

Transcript CpGC ID: Vu162906c4_g1_i13; **Treatment Time:** 60'; **Stress:** CPSMV+MI; **Kinase family:** CAMK_CAMKL-CHK1

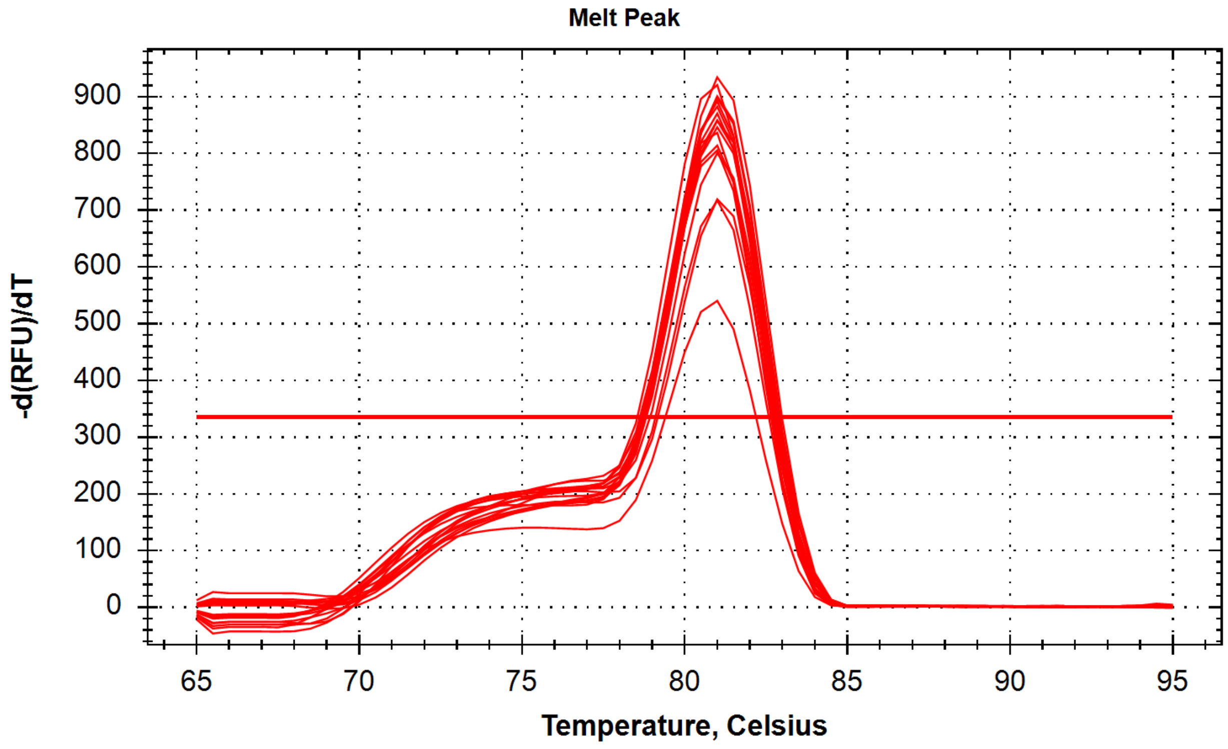

Transcript CpGC ID: Vu162906c4_g1_i13; **Treatment Time:** 150'; **Stress:** Root dehydration; **Kinase family:** CAMK_CAMKL-CHK1

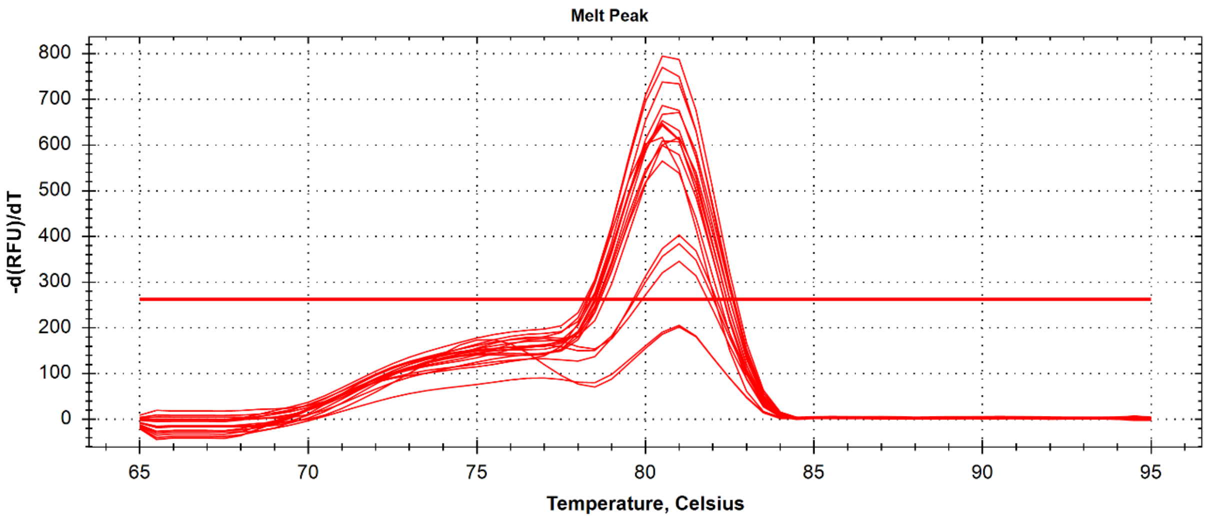

Supplement: Supplementary Appendix 1 — Melting curves for target transcripts used in the present study. [file Data_Sheet_1.PDF]
