# Supplementary material for: The Cowpea Kinome: Genomic and Transcriptomic Analysis Under Biotic and Abiotic Stresses
Source: Front Plant Sci. 2021 Jun 14;12:667013. doi: 10.3389/fpls.2021.667013 (PMC8238008; doi:10.3389/fpls.2021.667013)
Supplement: Supplementary Appendix 2 — Metrics for cowpea RNA-Seq libraries and transcriptome assembly. [file Data_Sheet_2.PDF]

Metrics for cowpea RNA-Seq libraries and transcriptome assembly

1. General aspects

Before transcriptome assembly, raw reads were trimmed from the 3' end to have a phred score of at least 30. Illumina sequencing adapters were removed from the reads, and all reads are required to have a length of at least 32 bp. After filtering, 602,713,788 (**Table 1**) paired-end high quality were obtained.

Table 1. Trimming statistics per sample.

Sample	Raw Paired Reads #	Surviving Paired Reads #	Surviving Paired Reads %
25aIT_ct60m	4,678,038	4,673,701	99.9
25bIT_ct60m	9,139,112	9,136,166	100.0
26aIT_ct60m	4,119,792	4,115,999	99.9
26bIT_ct60m	8,109,280	8,107,422	100.0
27aIT_ct60m	4,286,947	4,283,146	99.9
27bIT_ct60m	8,315,598	8,313,666	100.0
28aIT_vi60m	4,281,658	4,277,759	99.9
28bIT_vi60m	11,523,697	11,520,289	100.0
29aIT_vi60m	4,256,689	4,252,609	99.9
29bIT_vi60m	11,799,904	11,796,581	100.0
30aIT_vi60m	4,843,995	4,839,489	99.9
30bIT_vi60m	14,699,622	14,694,605	100.0
31aIT_ct6h	4,112,830	4,109,103	99.9
31bIT_ct6h	8,106,103	8,103,781	100.0
32aIT_ct6h	5,127,664	5,122,888	99.9
32bIT_ct6h	9,980,650	9,976,121	100.0
33aIT_ct6h	4,399,097	4,395,111	99.9
33bIT_ct6h	8,522,294	8,519,768	100.0
34aIT_vi16h	4,451,327	4,447,341	99.9
34bIT_vi16h	12,267,562	12,263,940	100.0
35aIT_vi16h	4,434,472	4,430,609	99.9
35bIT_vi16h	8,616,423	8,614,150	100.0
36aIT_vi16h	4,828,491	4,823,974	99.9
36bIT_vi16h	9,418,691	9,415,887	100.0
37aBR_ct60m	3,863,995	3,860,783	99.9
37bBR_ct60m	2,262,657	2,261,925	100.0
38aBR_ct60m	4,996,571	4,992,141	99.9
38bBR_ct60m	8,897,484	8,894,710	100.0
39aBR_ct60m	4,429,196	4,425,190	99.9

Sample	Raw Paired Reads #	Surviving Paired Reads #	Surviving Paired Reads %
39bBR_ct60m	13,767,519	13,762,839	100.0
40aBR_vi60m	5,245,543	5,240,931	99.9
40bBR_vi60m	9,324,340	9,322,518	100.0
41aBR_vi60m	5,039,749	5,035,222	99.9
41bBR_vi60m	8,973,401	8,971,036	100.0
42aBR_vi60m	5,476,870	5,471,883	99.9
42bBR_vi60m	16,010,419	16,004,114	100.0
43aBR_ct16h	5,105,699	5,101,223	99.9
43bBR_ct16h	9,211,938	9,209,571	100.0
44aBR_ct16h	4,619,304	4,615,312	99.9
44bBR_ct16h	8,350,834	8,348,891	100.0
45aBR_ct16h	4,707,586	4,703,405	99.9
45bBR_ct16h	8,341,597	8,339,555	100.0
46aBR_vi6h	6,312,916	6,307,222	99.9
46bBR_vi6h	11,272,191	11,269,344	100.0
47aBR_vi6h	5,236,226	5,231,413	99.9
47bBR_vi6h	9,316,597	9,313,739	100.0
48aBR_vi6h	5,693,257	5,688,168	99.9
48bBR_vi6h	10,030,120	10,026,092	100.0
A_2aPO_ct025m	11,429,847	11,418,092	99.9
B_2bPO_ct025m	11,020,746	11,011,524	99.9
C_1aPO_ct025m	12,717,867	12,705,286	99.9
D_1bPO_ct025m	12,279,457	12,269,445	99.9
E_11aPO_ct025m	8,609,876	8,601,190	99.9
F_11bPO_ct025m	8,336,138	8,329,349	99.9
G_8aPO_hy025m	10,258,592	10,248,653	99.9
H_8bPO_hy025m	9,926,617	9,918,886	99.9
I_12aPO_hy025m	9,235,256	9,226,235	99.9
J_12bPO_hy025m	8,933,797	8,926,624	99.9
K_9aPO_hy025m	10,843,132	10,830,913	99.9
L_9bPO_hy025m	10,438,903	10,428,877	99.9
M_5aPO_ct150m	11,791,559	11,778,898	99.9
N_5bPO_ct150m	11,375,961	11,365,872	99.9
O_3aPO_ct150m	10,644,357	10,633,829	99.9
P_3bPO_ct150m	10,281,161	10,272,892	99.9
Q_4aPO_ct150m	11,843,836	11,828,339	99.9
R_4bPO_ct150m	11,418,241	11,405,687	99.9
S_10aPO_hy150m	9,933,270	9,921,200	99.9
T_10bPO_hy150m	9,601,013	9,591,263	99.9
U_7aPO_hy150m	10,229,455	10,215,083	99.9
V_7bPO_hy150m	9,883,710	9,871,765	99.9
X_6aPO_hy150m	10,838,559	10,827,295	99.9

Sample	Raw Paired Reads #	Surviving Paired Reads #	Surviving Paired Reads %
Z_6bPO_hy150m	10,464,323	10,455,259	99.9

Legend: PO (Pingo de Ouro); IT (IT85F-2687); BR (BR14 mulato).

2. RNA-Seq assembly metrics

The 72 cowpea RNA-Seq libraries resulted in more than 602 million of paired reads, exhibiting a quality score of Q30 (100% accuracy). The contigs were assembled into 367,391 transcripts with an average length of 966.55 bp and an N50 length of 1721 (Table 1). All transcripts were longer than 223 bp and 30,19 % (110,921) of them were longer than 1,000 bp (Table 1).

Table 1. Assembly statistics of cowpea RNA-Seq libraries used in the present work.

Item	Value
Total number of paired reads	345,434,873
Total number of assembled transcripts	367,391
Assembled transcripts size in Mbp	355.10
Mean transcript length (nt)	966.55
Median contig length (nt)	385
N50 value of transcripts (nt)	1,721
Longest transcript length (nt)	16,271
Smallest transcript length (nt)	224
GC content (%)	39.65
Number of bases N	0
Number of sequences longer than 1k	110,921
Number of sequences longer than 10k	43
Total number of genes	243,130
