# Supplementary material for: The Cowpea Kinome: Genomic and Transcriptomic Analysis Under Biotic and Abiotic Stresses
Source: Front Plant Sci. 2021 Jun 14;12:667013. doi: 10.3389/fpls.2021.667013 (PMC8238008; doi:10.3389/fpls.2021.667013)
Supplement: Supplementary Appendix 3 — REST software outputs to target transcripts’ relative expression assay. [file Data_Sheet_3.PDF]

Supplemental Appendix S3. REST software outputs to target transcripts relative expression assay.

Relative Expression Report – REST Software

RD25' (Root Dehydration 25 min)

Parameter	Value
Iterations	10000

RESULTS

	Gene	Type	Reaction Efficiency	Expression	Std. Error	95% C.I.	P(H1)	Result
1	Vu118286|c0_g1_i1	TRG	1,0	1,561	1,240 - 2,030	0,969 - 2,576	0,001	UR
2	Vu64579|c0_g1_i1	TRG	1,0	2,078	1,629 - 2,726	1,283 - 3,434	0,000	UR
3	Vu14294|c0_g1_i1	TRG	1,0	35,044	17,953 - 72,648	11,959 - 128,890	0,000	UR
4	Vu2805|c5_g1_i1	TRG	1,0	2,304	1,155 - 4,673	0,727 - 6,916	0,002	UR
	UE21D	REF	1,0	1,121				
	ACT	REF	1,0	0,892				

Legend: P(H1) - Probability of alternate hypothesis that difference between sample and control groups is due only to chance; TRG – Target gene; REF – Reference gene; UR – up-regulated.

Interpretation

1. **Vu118286|c0_g1_i1** is UP-regulated in sample group (in comparison to control group) by a mean factor of 1,561 (S.E. range is 1,240 - 2,030).

Vu118286|c0_g1_i1 sample group is different to control group. P(H1)=0,001

2. **Vu64579|c0_g1_i1** is UP-regulated in sample group (in comparison to control group) by a mean factor of 2,078 (S.E. range is 1,629 - 2,726).

Vu64579|c0_g1_i1 sample group is different to control group. P(H1)=0,000

3. **Vu14294|c0_g1_i1** is UP-regulated in sample group (in comparison to control group) by a mean factor of 35,044 (S.E. range is 17,953 - 72,648).

Vu14294|c0_g1_i1 sample group is different to control group. P(H1)=0,000

4. **Vu2805|c5_g1_i1** is UP-regulated in sample group (in comparison to control group) by a mean factor of 2,304 (S.E. range is 1,155 - 4,673).

Vu2805|c5_g1_i1 sample group is different to control group. P(H1)=0,002

RD150' (Root Dehydration 150 min)

Parameter	Value
Iterations	10000

RESULTS

	Gene	Type	Reaction Efficiency	Expression	Std. Error	95% C.I.	P(H1)	Result
1	Vu23437|c2_g1_i21	TRG	0,942	1,94	1,422 - 2,629	0,930 - 3,934	0,000	UR
2	Vu162906|c4_g1_i13	TRG	1,0	5,337	2,793 - 10,666	1,500 - 16,167	0,000	UR
3	Vu76468|c0_g3_i1	TRG	0,904	17,841	9,079 - 38,377	4,762 - 72,386	0,000	UR
4	Vu2805|c5_g1_i1	TRG	1,0	4,413	2,494 - 8,204	1,292 - 11,119	0,000	UR
5	Vu14294|c0_g1_i1	TRG	1,0	16,211	4,517 - 62,321	1,613 - 126,238	0,000	UR
	UE21D	REF	1,0	0,973				
	ACT	REF	1,0	1,028				

Legend: P(H1) - Probability of alternate hypothesis that difference between sample and control groups is due only to chance; TRG – Target gene; REF – Reference gene; UR – up-regulated.

Interpretation

1. **Vu23437|c2_g1_i21** is UP-regulated in sample group (in comparison to control group) by a mean factor of 1,940 (S.E. range is 1,422 - 2,629).

Vu23437|c2_g1_i21 sample group is different to control group. P(H1)=0,000

2. **Vu162906|c4_g1_i13** is UP-regulated in sample group (in comparison to control group) by a mean factor of 5,337 (S.E. range is 2,793 - 10,666).

Vu162906|c4_g1_i13 sample group is different to control group. P(H1)=0,000

3. **Vu76468|c0_g3_i1** is UP-regulated in sample group (in comparison to control group) by a mean factor of 17,841 (S.E. range is 9,079 - 38,377).

Vu76468|c0_g3_i1 sample group is different to control group. P(H1)=0,000

4. **Vu2805|c5_g1_i1** is UP-regulated in sample group (in comparison to control group) by a mean factor of 4,413 (S.E. range is 2,494 - 8,204).

Vu2805|c5_g1_i1 sample group is different to control group. P(H1)=0,000

5. **Vu14294|c0_g1_i1** is UP-regulated in sample group (in comparison to control group) by a mean factor of 16,211 (S.E. range is 4,517 - 62,321).

Vu14294|c0_g1_i1 sample group is different to control group. P(H1)=0,000

CPSMV (*Cowpea severe mosaic virus*)

Parameter	Value
Iterations	10000

RESULTS

	Gene	Type	Reaction Efficiency	Expression	Std. Error	95% C.I.	P(H1)	Result
1	Vu4603|c0_g2_i1	TRG	1,0	3,039	1,743 - 6,166	1,132 - 8,173	0,000	UR
2	Vu64579|c0_g1_i1	TRG	1,0	1,845	0,874 - 4,036	0,491 - 8,475	0,036	UR
3	Vu162906|c4_g1_i13	TRG	1,0	1,481	0,966 - 2,156	0,716 - 4,169	0,021	UR
4	Vu118286|c0_g1_i1	TRG	1,0	2,504	1,420 - 4,738	0,870 - 8,391	0,000	UR
5 - 60min	Vu23437|c2_g1_i21	TRG	0,942	1,582	0,951 - 2,516	0,801 - 4,639	0,018	UR
6 - 16h	Vu23437|c2_g1_i21	TRG	0,942	1,575	0,900 - 2,783	0,705 - 6,010	0,049	UR
	FBOX	REF	1,0	0,947				
	UBQ10	REF	0,97	1,056				

Legend: P(H1) - Probability of alternate hypothesis that difference between sample and control groups is due only to chance; TRG – Target gene; REF – Reference gene; UR – up-regulated.

Interpretation

1. **Vu4603|c0_g2_i1** is UP-regulated in sample group (in comparison to control group) by a mean factor of 3,039 (S.E. range is 1,743 - 6,166).

Vu4603|c0_g2_i1 sample group is different to control group. P(H1)=0,000

2. **Vu64579|c0_g1_i1** is UP-regulated in sample group (in comparison to control group) by a mean factor of 1,845 (S.E. range is 0,874 - 4,036).

Vu64579|c0_g1_i1 sample group is different to control group. P(H1)=0,036

3. **Vu162906|c4_g1_i13** is UP-regulated in sample group (in comparison to control group) by a mean factor of 1,481 (S.E. range is 0,966 - 2,156).

Vu162906|c4_g1_i13 sample group is different to control group. P(H1)=0,021

4. **Vu118286|c0_g1_i1** is UP-regulated in sample group (in comparison to control group) by a mean factor of 2,504 (S.E. range is 1,420 - 4,738).

Vu118286|c0_g1_i1 sample group is different to control group. P(H1)=0,000

5. **Vu23437|c2_g1_i21** is UP-regulated in sample group (in comparison to control group) by a mean factor of 1,582 (S.E. range is 0,951 - 2,516).

Vu23437|c2_g1_i21 sample group is different to control group. P(H1)=0,018

6. **Vu23437|c2_g1_i21** is UP-regulated in sample group (in comparison to control group) by a mean factor of 1,575 (S.E. range is 0,900 - 2,783).

Vu23437|c2_g1_i21 sample group is different to control group. P(H1)=0,049

CABMV (*Cowpea aphid-born mosaic virus*)

	Gene	Type	Reaction Efficiency	Expression	Std. Error	95% C.I.	P(H1)	Result
1	Vu4603|c0_g2_i1	TRG	1,0	5,343	2,005 - 11,319	1,175 - 32,834	0,000	UR
2	Vu118286|c0_g1_i1	TRG	1,0	28,71	19,755 - 40,699	15,096 - 59,734	0,000	UR
3	Vu162906|c4_g1_i13	TRG	1,0	1,236	0,864 - 1,613	0,766 - 2,106	0,049	UR
4	Vu116133|c3_g1_i4	TRG	1,0	1,465	1,109 - 1,948	0,649 - 2,820	0,008	UR
5	Vu158944|c1_g2_i10	TRG	1,0	1,52	1,262 - 1,756	1,158 - 2,152	0,000	UR
	FBOX	REF	1,0	1,068				
	UBQ10	REF	0,97	0,937				

Legend: P(H1) - Probability of alternate hypothesis that difference between sample and control groups is due only to chance; TRG – Target gene; REF – Reference gene; UR – up-regulated.

Interpretation

1. **Vu4603|c0_g2_i1** is UP-regulated in sample group (in comparison to control group) by a mean factor of 5,343 (S.E. range is 2,005 - 11,319).

Vu4603|c0_g2_i1 sample group is different to control group. P(H1)=0,000

2. **Vu118286|c0_g1_i1** is UP-regulated in sample group (in comparison to control group) by a mean factor of 28,710 (S.E. range is 19,755 - 40,699).

Vu118286|c0_g1_i1 sample group is different to control group. P(H1)=0,000

3. **Vu162906|c4_g1_i13** is UP-regulated in sample group (in comparison to control group) by a mean factor of 1,236 (S.E. range is 0,864 - 1,613).

Vu162906|c4_g1_i13 sample group is different to control group. P(H1)=0,049

4. **Vu116133|c3_g1_i4** is UP-regulated in sample group (in comparison to control group) by a mean factor of 1,465 (S.E. range is 1,109 - 1,948).

Vu116133|c3_g1_i4 sample group is different to control group. P(H1)=0,008

5. **Vu158944|c1_g2_i10** is UP-regulated in sample group (in comparison to control group) by a mean factor of 1,465 (S.E. range is 1,109 - 1,948).

Vu158944|c1_g2_i10 sample group is different to control group. P(H1)=0,012
