# Supplementary material for: The Cowpea Kinome: Genomic and Transcriptomic Analysis Under Biotic and Abiotic Stresses
Source: Front Plant Sci. 2021 Jun 14;12:667013. doi: 10.3389/fpls.2021.667013 (PMC8238008; doi:10.3389/fpls.2021.667013)
Supplement: Supplementary Figure 4 — qPCR results for a sample of up-regulated VuPKs obtained from the RNA-Seq assays. (A) Mechanical injury (MI) + CABMV (cowpea aphid-borne mosaic virus) inoculation, (B) MI + CPSMV (cowpea severe mosaic virus) inoculation, and (C) root dehydration (RD). The asterisk indicates being differentially expressed at p < 0.05. Black circles: 1—up-regulated crosstalk response among MI + CABMV, MI + CPSMV, and RD assays; 2—up-regulated crosstalk response between MI + CABMV and MI + CPSMV assays; and 3—up-regulated crosstalk response between MI + CPSMV and RD assays. [file Data_Sheet_4.PDF]

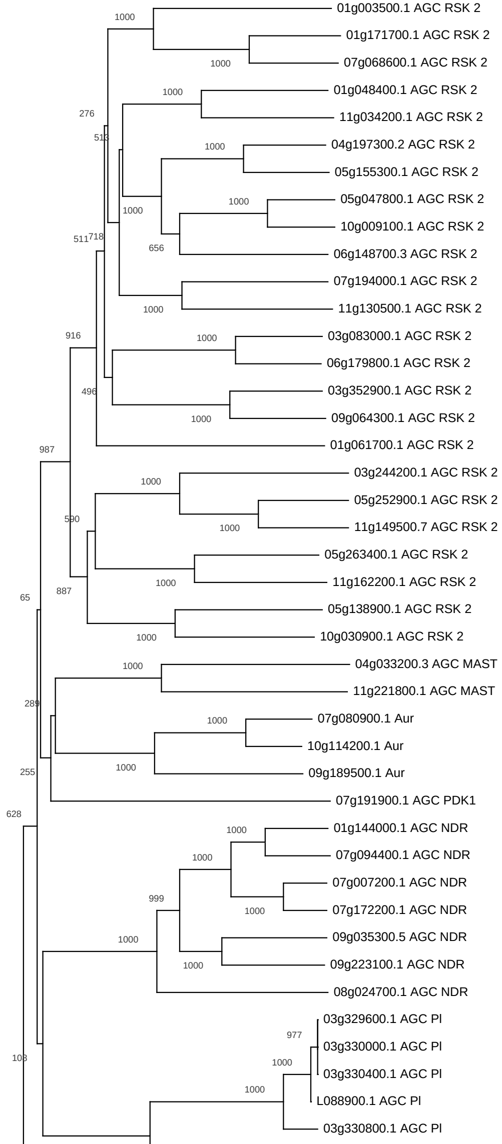

Supplemental Figure S3. Neighbor Joining distance tree illustrating VuPKs phenetic relationships. Values at nodes correspond to the bootstrap values. Legend: UNK (unknown) refers to VuPKs whose phenetic grouping did not occur with their peers (based on iTAK classification).

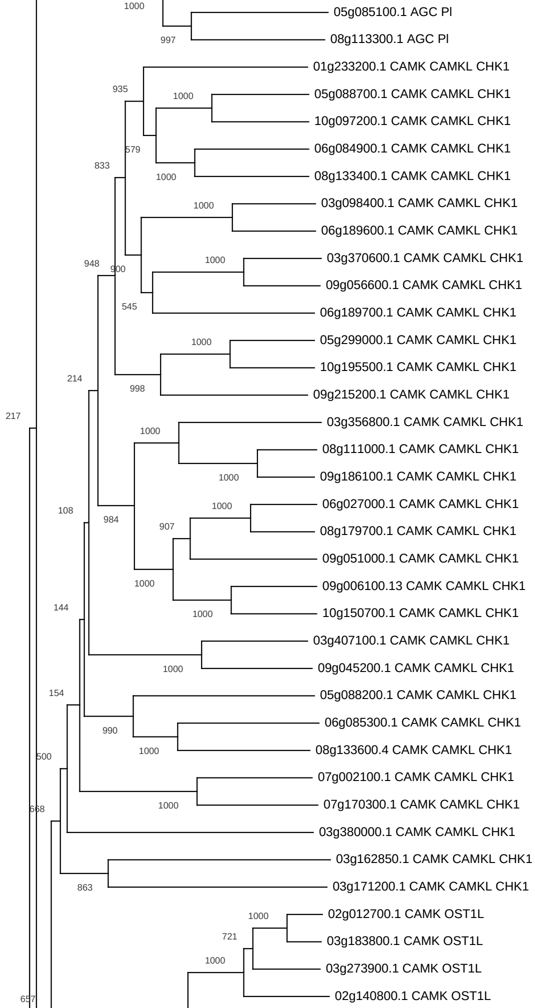
05g085100.1 AGC PI
08g113300.1 AGC PI
01g233200.1 CAMK CAMKL CHK1
05g088700.1 CAMK CAMKL CHK1
10g097200.1 CAMK CAMKL CHK1
06g084900.1 CAMK CAMKL CHK1
08g133400.1 CAMK CAMKL CHK1
03g098400.1 CAMK CAMKL CHK1
06g189600.1 CAMK CAMKL CHK1
03g370600.1 CAMK CAMKL CHK1
09g056600.1 CAMK CAMKL CHK1
06g189700.1 CAMK CAMKL CHK1
05g299000.1 CAMK CAMKL CHK1
10g195500.1 CAMK CAMKL CHK1
09g215200.1 CAMK CAMKL CHK1
03g356800.1 CAMK CAMKL CHK1
08g111000.1 CAMK CAMKL CHK1
09g186100.1 CAMK CAMKL CHK1
06g027000.1 CAMK CAMKL CHK1
08g179700.1 CAMK CAMKL CHK1
09g051000.1 CAMK CAMKL CHK1
09g006100.13 CAMK CAMKL CHK1
10g150700.1 CAMK CAMKL CHK1
03g407100.1 CAMK CAMKL CHK1
09g045200.1 CAMK CAMKL CHK1
05g088200.1 CAMK CAMKL CHK1
06g085300.1 CAMK CAMKL CHK1
08g133600.4 CAMK CAMKL CHK1
07g002100.1 CAMK CAMKL CHK1
07g170300.1 CAMK CAMKL CHK1
03g380000.1 CAMK CAMKL CHK1
03g162850.1 CAMK CAMKL CHK1
03g171200.1 CAMK CAMKL CHK1
02g012700.1 CAMK OST1L
03g183800.1 CAMK OST1L
03g273900.1 CAMK OST1L
02g140800.1 CAMK OST1L

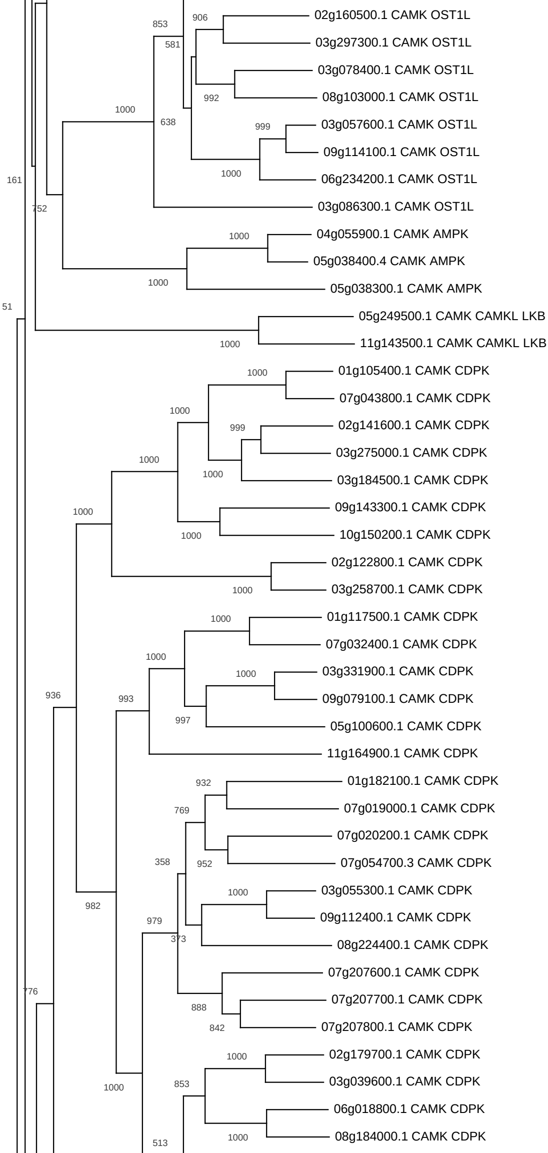
02g160500.1 CAMK OST1L
03g297300.1 CAMK OST1L
03g078400.1 CAMK OST1L
08g103000.1 CAMK OST1L
03g057600.1 CAMK OST1L
09g114100.1 CAMK OST1L
06g234200.1 CAMK OST1L
03g086300.1 CAMK OST1L
04g055900.1 CAMK AMPK
05g038400.4 CAMK AMPK
05g038300.1 CAMK AMPK
05g249500.1 CAMK CAMKL LKB
11g143500.1 CAMK CAMKL LKB
01g105400.1 CAMK CDPK
07g043800.1 CAMK CDPK
02g141600.1 CAMK CDPK
03g275000.1 CAMK CDPK
03g184500.1 CAMK CDPK
09g143300.1 CAMK CDPK
10g150200.1 CAMK CDPK
02g122800.1 CAMK CDPK
03g258700.1 CAMK CDPK
01g117500.1 CAMK CDPK
07g032400.1 CAMK CDPK
03g331900.1 CAMK CDPK
09g079100.1 CAMK CDPK
05g100600.1 CAMK CDPK
11g164900.1 CAMK CDPK
01g182100.1 CAMK CDPK
07g019000.1 CAMK CDPK
07g020200.1 CAMK CDPK
07g054700.3 CAMK CDPK
03g055300.1 CAMK CDPK
09g112400.1 CAMK CDPK
08g224400.1 CAMK CDPK
07g207600.1 CAMK CDPK
07g207700.1 CAMK CDPK
07g207800.1 CAMK CDPK
02g179700.1 CAMK CDPK
03g039600.1 CAMK CDPK
06g018800.1 CAMK CDPK
08g184000.1 CAMK CDPK

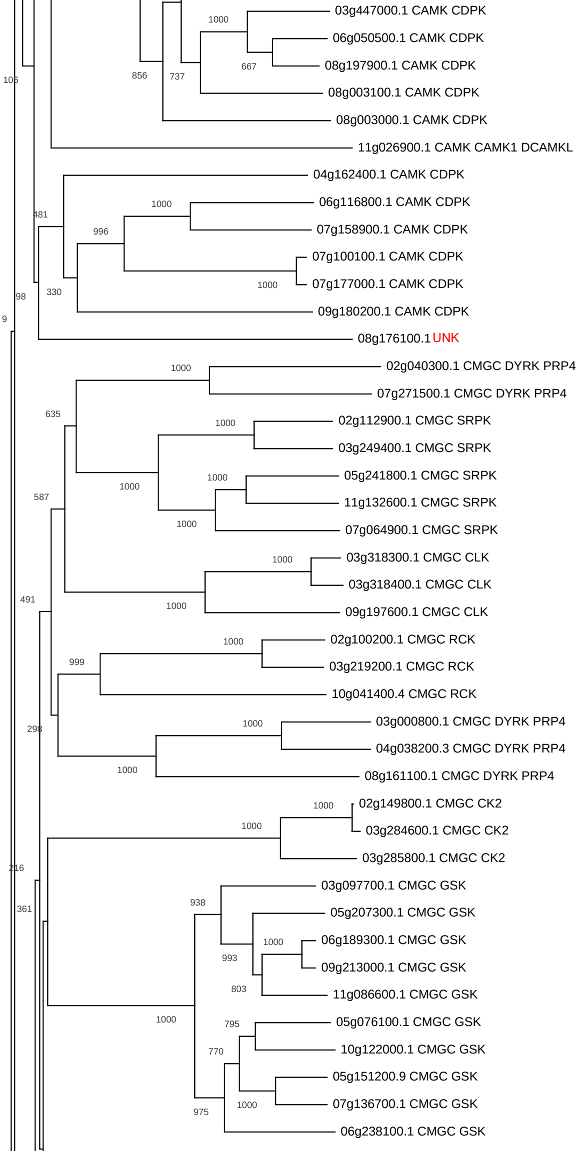

03g447000.1 CAMK CDPK
1000
06g050500.1 CAMK CDPK
667
08g197900.1 CAMK CDPK
106
856
737
08g003100.1 CAMK CDPK
08g003000.1 CAMK CDPK
11g026900.1 CAMK CAMK1 DCAMKL
04g162400.1 CAMK CDPK
1000
06g116800.1 CAMK CDPK
481
996
07g158900.1 CAMK CDPK
07g100100.1 CAMK CDPK
1000
07g177000.1 CAMK CDPK
98
330
09g180200.1 CAMK CDPK
9
08g176100.1 UNK
1000
02g040300.1 CMGC DYRK PRP4
07g271500.1 CMGC DYRK PRP4
635
1000
02g112900.1 CMGC SRPK
03g249400.1 CMGC SRPK
1000
05g241800.1 CMGC SRPK
1000
587
11g132600.1 CMGC SRPK
1000
07g064900.1 CMGC SRPK
1000
03g318300.1 CMGC CLK
03g318400.1 CMGC CLK
491
1000
09g197600.1 CMGC CLK
1000
02g100200.1 CMGC RCK
999
03g219200.1 CMGC RCK
10g041400.4 CMGC RCK
1000
03g000800.1 CMGC DYRK PRP4
298
04g038200.3 CMGC DYRK PRP4
1000
08g161100.1 CMGC DYRK PRP4
1000
02g149800.1 CMGC CK2
1000
03g284600.1 CMGC CK2
03g285800.1 CMGC CK2
216
03g097700.1 CMGC GSK
938
361
05g207300.1 CMGC GSK
1000
06g189300.1 CMGC GSK
993
09g213000.1 CMGC GSK
803
11g086600.1 CMGC GSK
1000
05g076100.1 CMGC GSK
795
770
10g122000.1 CMGC GSK
05g151200.9 CMGC GSK
1000
07g136700.1 CMGC GSK
975
06g238100.1 CMGC GSK

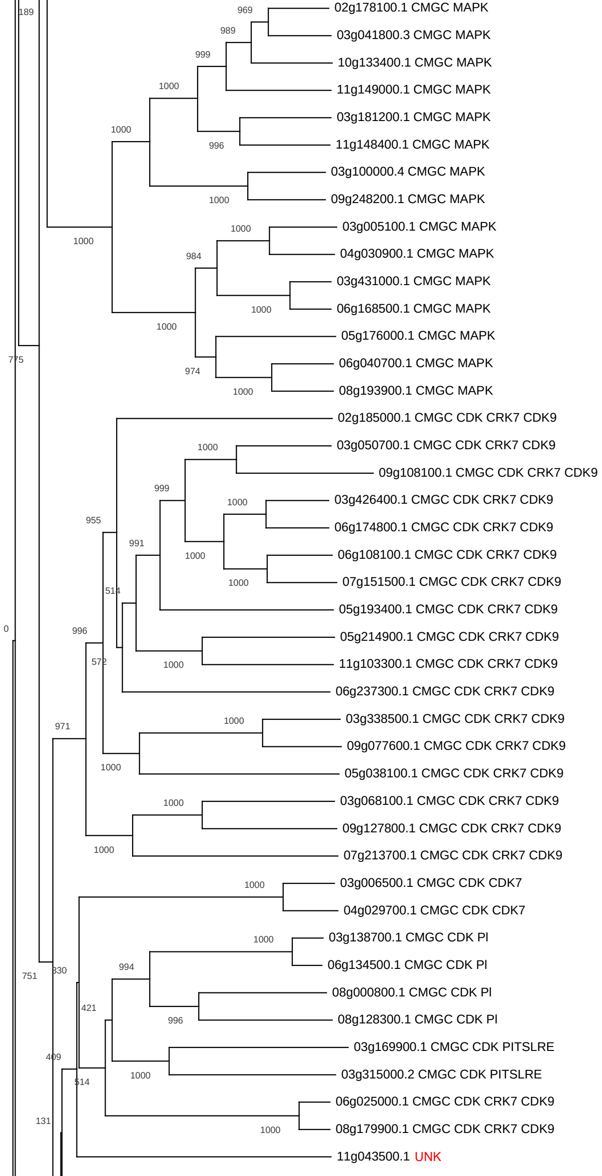

02g178100.1 CMGC MAPK
03g041800.3 CMGC MAPK
10g133400.1 CMGC MAPK
11g149000.1 CMGC MAPK
03g181200.1 CMGC MAPK
11g148400.1 CMGC MAPK
03g100000.4 CMGC MAPK
09g248200.1 CMGC MAPK
03g005100.1 CMGC MAPK
04g030900.1 CMGC MAPK
03g431000.1 CMGC MAPK
06g168500.1 CMGC MAPK
05g176000.1 CMGC MAPK
06g040700.1 CMGC MAPK
08g193900.1 CMGC MAPK
02g185000.1 CMGC CDK CRK7 CDK9
03g050700.1 CMGC CDK CRK7 CDK9
09g108100.1 CMGC CDK CRK7 CDK9
03g426400.1 CMGC CDK CRK7 CDK9
06g174800.1 CMGC CDK CRK7 CDK9
06g108100.1 CMGC CDK CRK7 CDK9
07g151500.1 CMGC CDK CRK7 CDK9
05g193400.1 CMGC CDK CRK7 CDK9
05g214900.1 CMGC CDK CRK7 CDK9
11g103300.1 CMGC CDK CRK7 CDK9
06g237300.1 CMGC CDK CRK7 CDK9
03g338500.1 CMGC CDK CRK7 CDK9
09g077600.1 CMGC CDK CRK7 CDK9
05g038100.1 CMGC CDK CRK7 CDK9
03g068100.1 CMGC CDK CRK7 CDK9
09g127800.1 CMGC CDK CRK7 CDK9
07g213700.1 CMGC CDK CRK7 CDK9
03g006500.1 CMGC CDK CDK7
04g029700.1 CMGC CDK CDK7
03g138700.1 CMGC CDK PI
06g134500.1 CMGC CDK PI
08g000800.1 CMGC CDK PI
08g128300.1 CMGC CDK PI
03g169900.1 CMGC CDK PITSLRE
03g315000.2 CMGC CDK PITSLRE
06g025000.1 CMGC CDK CRK7 CDK9
08g179900.1 CMGC CDK CRK7 CDK9
11g043500.1 UNK

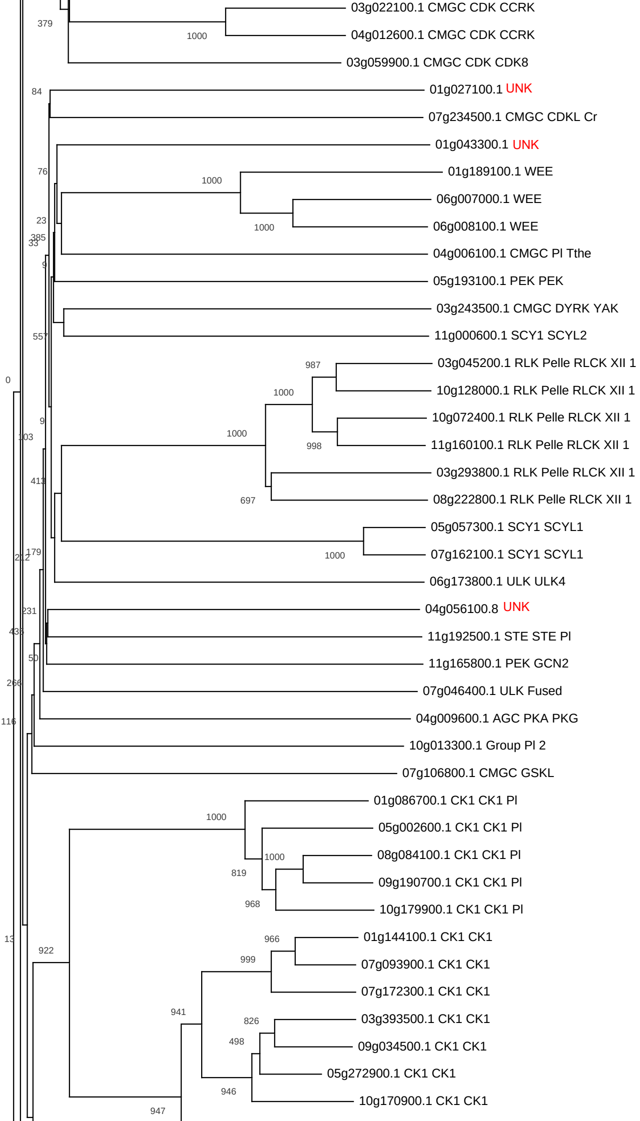
03g022100.1 CMGC CDK CCRK
04g012600.1 CMGC CDK CCRK
03g059900.1 CMGC CDK CDK8
01g027100.1 UNK
07g234500.1 CMGC CDKL Cr
01g043300.1 UNK
01g189100.1 WEE
06g007000.1 WEE
06g008100.1 WEE
04g006100.1 CMGC Pl Tthe
05g193100.1 PEK PEK
03g243500.1 CMGC DYRK YAK
11g000600.1 SCY1 SCYL2
03g045200.1 RLK Pelle RLCK XII 1
10g128000.1 RLK Pelle RLCK XII 1
10g072400.1 RLK Pelle RLCK XII 1
11g160100.1 RLK Pelle RLCK XII 1
03g293800.1 RLK Pelle RLCK XII 1
08g222800.1 RLK Pelle RLCK XII 1
05g057300.1 SCY1 SCYL1
07g162100.1 SCY1 SCYL1
06g173800.1 ULK ULK4
04g056100.8 UNK
11g192500.1 STE STE Pl
11g165800.1 PEK GCN2
07g046400.1 ULK Fused
04g009600.1 AGC PKA PKG
10g013300.1 Group Pl 2
07g106800.1 CMGC GSKL
01g086700.1 CK1 CK1 Pl
05g002600.1 CK1 CK1 Pl
08g084100.1 CK1 CK1 Pl
09g190700.1 CK1 CK1 Pl
10g179900.1 CK1 CK1 Pl
01g144100.1 CK1 CK1
07g093900.1 CK1 CK1
07g172300.1 CK1 CK1
03g393500.1 CK1 CK1
09g034500.1 CK1 CK1
05g272900.1 CK1 CK1
10g170900.1 CK1 CK1
379
1000
84
76
1000
1000
23
385
33
9
557
0
987
1000
9
103
1000
998
413
697
179
212
1000
231
436
50
266
116
1000
1000
819
968
13
966
922
999
941
826
498
946
947

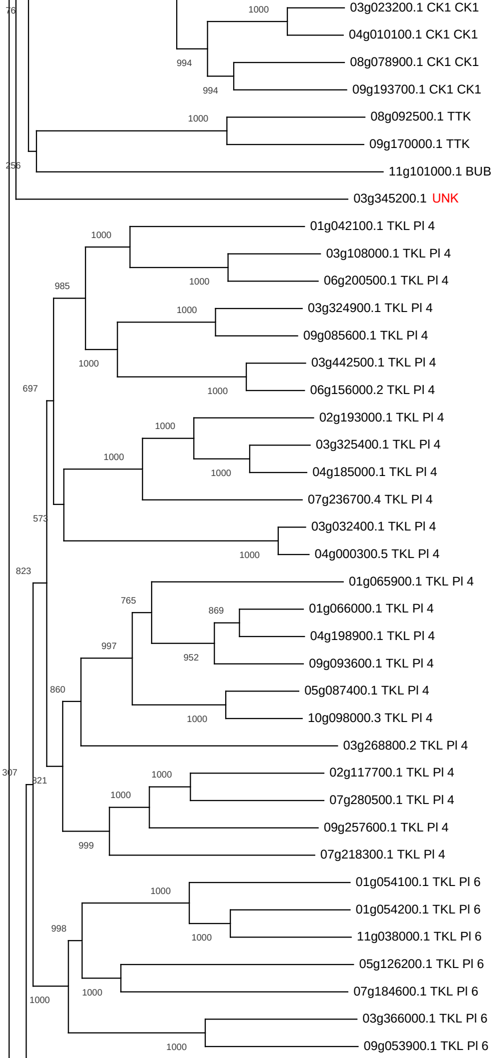
76
1000
03g023200.1 CK1 CK1
04g010100.1 CK1 CK1
994
08g078900.1 CK1 CK1
994
09g193700.1 CK1 CK1
1000
08g092500.1 TTK
09g170000.1 TTK
256
11g101000.1 BUB
03g345200.1 UNK
1000
01g042100.1 TKL Pl 4
03g108000.1 TKL Pl 4
1000
06g200500.1 TKL Pl 4
985
1000
03g324900.1 TKL Pl 4
09g085600.1 TKL Pl 4
1000
03g442500.1 TKL Pl 4
697
1000
06g156000.2 TKL Pl 4
02g193000.1 TKL Pl 4
1000
03g325400.1 TKL Pl 4
1000
1000
04g185000.1 TKL Pl 4
07g236700.4 TKL Pl 4
573
03g032400.1 TKL Pl 4
1000
04g000300.5 TKL Pl 4
823
01g065900.1 TKL Pl 4
765
869
01g066000.1 TKL Pl 4
04g198900.1 TKL Pl 4
997
952
09g093600.1 TKL Pl 4
860
05g087400.1 TKL Pl 4
1000
10g098000.3 TKL Pl 4
03g268800.2 TKL Pl 4
307
821
1000
02g117700.1 TKL Pl 4
1000
07g280500.1 TKL Pl 4
09g257600.1 TKL Pl 4
999
07g218300.1 TKL Pl 4
01g054100.1 TKL Pl 6
1000
01g054200.1 TKL Pl 6
998
1000
11g038000.1 TKL Pl 6
05g126200.1 TKL Pl 6
1000
1000
07g184600.1 TKL Pl 6
03g366000.1 TKL Pl 6
1000
09g053900.1 TKL Pl 6

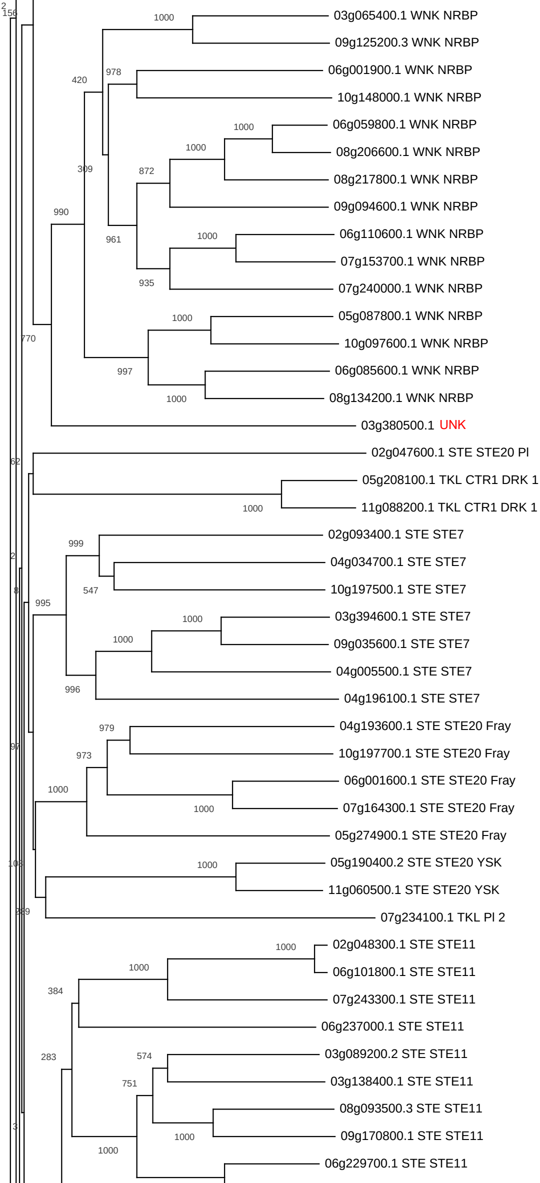

03g065400.1 WNK NRBP
09g125200.3 WNK NRBP
06g001900.1 WNK NRBP
10g148000.1 WNK NRBP
06g059800.1 WNK NRBP
08g206600.1 WNK NRBP
08g217800.1 WNK NRBP
09g094600.1 WNK NRBP
06g110600.1 WNK NRBP
07g153700.1 WNK NRBP
07g240000.1 WNK NRBP
05g087800.1 WNK NRBP
10g097600.1 WNK NRBP
06g085600.1 WNK NRBP
08g134200.1 WNK NRBP
03g380500.1 UNK
02g047600.1 STE STE20 Pl
05g208100.1 TKL CTR1 DRK 1
11g088200.1 TKL CTR1 DRK 1
02g093400.1 STE STE7
04g034700.1 STE STE7
10g197500.1 STE STE7
03g394600.1 STE STE7
09g035600.1 STE STE7
04g005500.1 STE STE7
04g196100.1 STE STE7
04g193600.1 STE STE20 Fray
10g197700.1 STE STE20 Fray
06g001600.1 STE STE20 Fray
07g164300.1 STE STE20 Fray
05g274900.1 STE STE20 Fray
05g190400.2 STE STE20 YSK
11g060500.1 STE STE20 YSK
07g234100.1 TKL Pl 2
02g048300.1 STE STE11
06g101800.1 STE STE11
07g243300.1 STE STE11
06g237000.1 STE STE11
03g089200.2 STE STE11
03g138400.1 STE STE11
08g093500.3 STE STE11
09g170800.1 STE STE11
06g229700.1 STE STE11

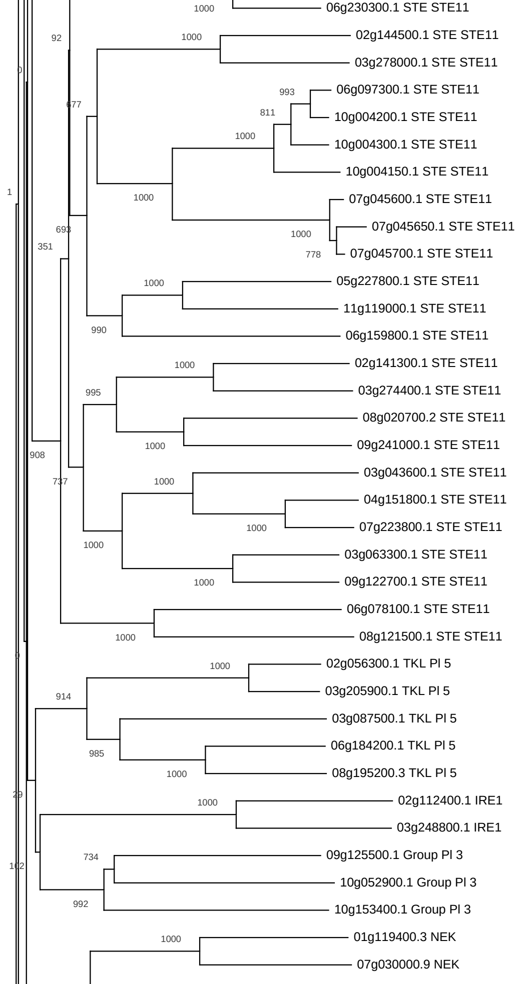

06g230300.1 STE STE11
02g144500.1 STE STE11
03g278000.1 STE STE11
06g097300.1 STE STE11
10g004200.1 STE STE11
10g004300.1 STE STE11
10g004150.1 STE STE11
07g045600.1 STE STE11
07g045650.1 STE STE11
07g045700.1 STE STE11
05g227800.1 STE STE11
11g119000.1 STE STE11
06g159800.1 STE STE11
02g141300.1 STE STE11
03g274400.1 STE STE11
08g020700.2 STE STE11
09g241000.1 STE STE11
03g043600.1 STE STE11
04g151800.1 STE STE11
07g223800.1 STE STE11
03g063300.1 STE STE11
09g122700.1 STE STE11
06g078100.1 STE STE11
08g121500.1 STE STE11
02g056300.1 TKL Pl 5
03g205900.1 TKL Pl 5
03g087500.1 TKL Pl 5
06g184200.1 TKL Pl 5
08g195200.3 TKL Pl 5
02g112400.1 IRE1
03g248800.1 IRE1
09g125500.1 Group Pl 3
10g052900.1 Group Pl 3
10g153400.1 Group Pl 3
01g119400.3 NEK
07g030000.9 NEK

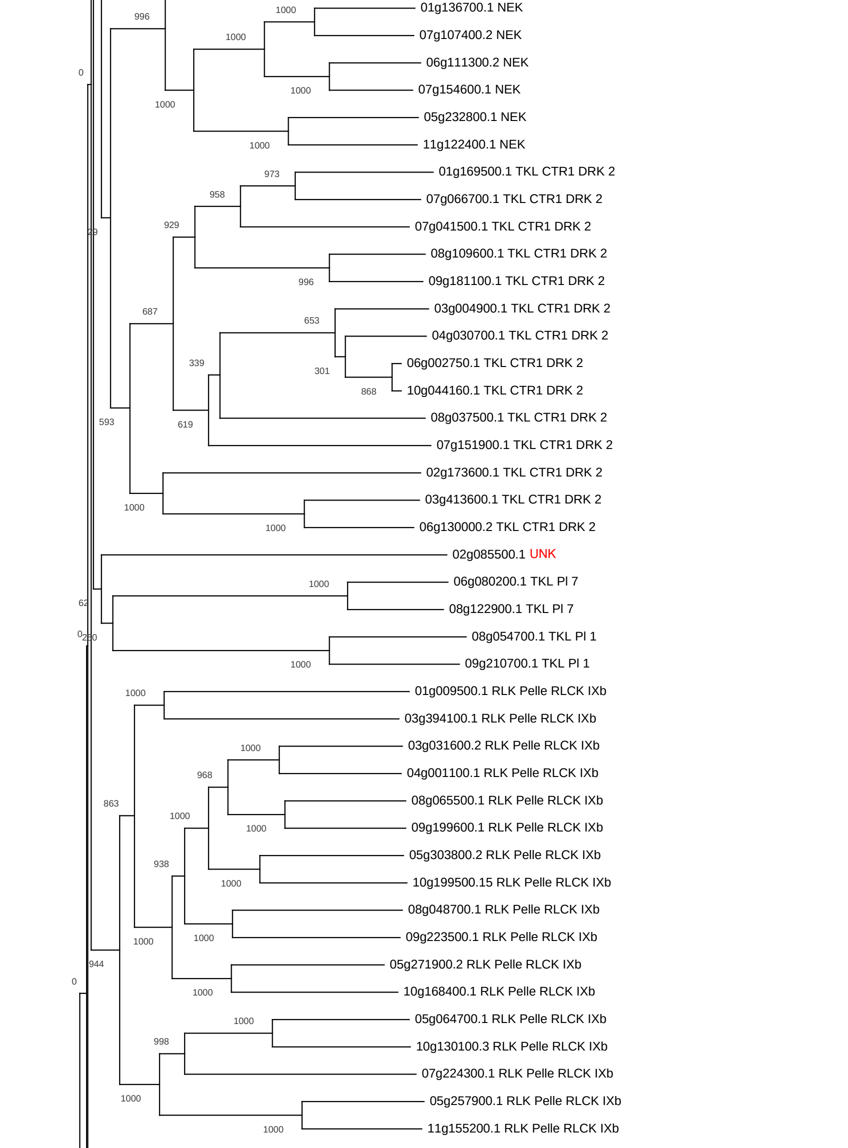
01g136700.1 NEK
07g107400.2 NEK
06g111300.2 NEK
07g154600.1 NEK
05g232800.1 NEK
11g122400.1 NEK
01g169500.1 TKL CTR1 DRK 2
07g066700.1 TKL CTR1 DRK 2
07g041500.1 TKL CTR1 DRK 2
08g109600.1 TKL CTR1 DRK 2
09g181100.1 TKL CTR1 DRK 2
03g004900.1 TKL CTR1 DRK 2
04g030700.1 TKL CTR1 DRK 2
06g002750.1 TKL CTR1 DRK 2
10g044160.1 TKL CTR1 DRK 2
08g037500.1 TKL CTR1 DRK 2
07g151900.1 TKL CTR1 DRK 2
02g173600.1 TKL CTR1 DRK 2
03g413600.1 TKL CTR1 DRK 2
06g130000.2 TKL CTR1 DRK 2
02g085500.1 UNK
06g080200.1 TKL Pl 7
08g122900.1 TKL Pl 7
08g054700.1 TKL Pl 1
09g210700.1 TKL Pl 1
01g009500.1 RLK Pelle RLCK IXb
03g394100.1 RLK Pelle RLCK IXb
03g031600.2 RLK Pelle RLCK IXb
04g001100.1 RLK Pelle RLCK IXb
08g065500.1 RLK Pelle RLCK IXb
09g199600.1 RLK Pelle RLCK IXb
05g303800.2 RLK Pelle RLCK IXb
10g199500.15 RLK Pelle RLCK IXb
08g048700.1 RLK Pelle RLCK IXb
09g223500.1 RLK Pelle RLCK IXb
05g271900.2 RLK Pelle RLCK IXb
10g168400.1 RLK Pelle RLCK IXb
05g064700.1 RLK Pelle RLCK IXb
10g130100.3 RLK Pelle RLCK IXb
07g224300.1 RLK Pelle RLCK IXb
05g257900.1 RLK Pelle RLCK IXb
11g155200.1 RLK Pelle RLCK IXb
996
1000
1000
0
1000
1000
1000
973
958
929
29
996
687
653
339
301
868
593
619
1000
1000
1000
62
0
250
1000
1000
1000
968
863
1000
1000
938
1000
1000
1000
944
0
1000
1000
998
1000
1000

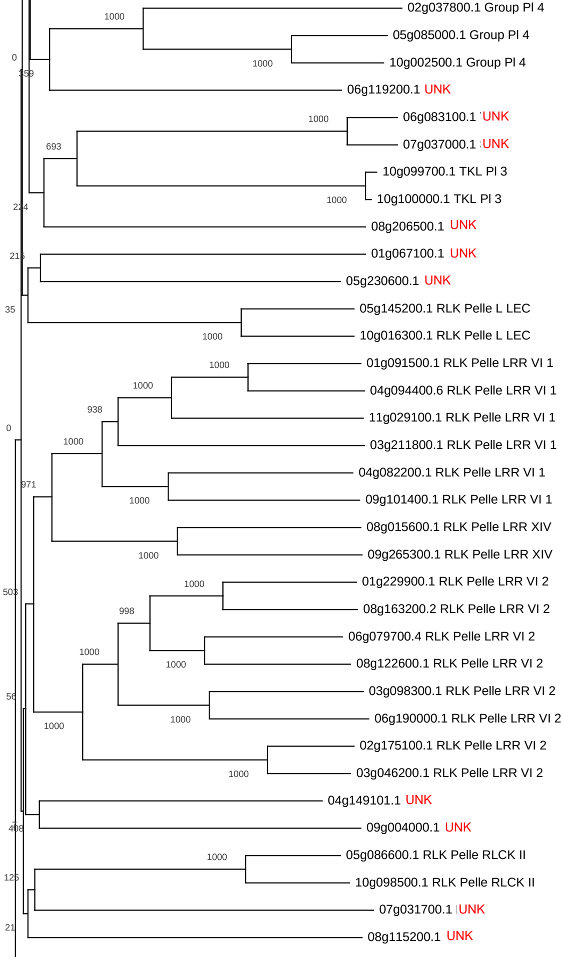

02g037800.1 Group Pl 4
05g085000.1 Group Pl 4
10g002500.1 Group Pl 4
06g119200.1 UNK
06g083100.1 UNK
07g037000.1 UNK
10g099700.1 TKL Pl 3
10g100000.1 TKL Pl 3
08g206500.1 UNK
01g067100.1 UNK
05g230600.1 UNK
05g145200.1 RLK Pelle L LEC
10g016300.1 RLK Pelle L LEC
01g091500.1 RLK Pelle LRR VI 1
04g094400.6 RLK Pelle LRR VI 1
11g029100.1 RLK Pelle LRR VI 1
03g211800.1 RLK Pelle LRR VI 1
04g082200.1 RLK Pelle LRR VI 1
09g101400.1 RLK Pelle LRR VI 1
08g015600.1 RLK Pelle LRR XIV
09g265300.1 RLK Pelle LRR XIV
01g229900.1 RLK Pelle LRR VI 2
08g163200.2 RLK Pelle LRR VI 2
06g079700.4 RLK Pelle LRR VI 2
08g122600.1 RLK Pelle LRR VI 2
03g098300.1 RLK Pelle LRR VI 2
06g190000.1 RLK Pelle LRR VI 2
02g175100.1 RLK Pelle LRR VI 2
03g046200.1 RLK Pelle LRR VI 2
04g149101.1 UNK
09g004000.1 UNK
05g086600.1 RLK Pelle RLCK II
10g098500.1 RLK Pelle RLCK II
07g031700.1 UNK
08g115200.1 UNK
1000
1000
0
359
1000
693
1000
224
215
35
1000
1000
1000
938
0
1000
971
1000
1000
1000
503
998
1000
1000
56
1000
1000
1000
408
1000
125
21

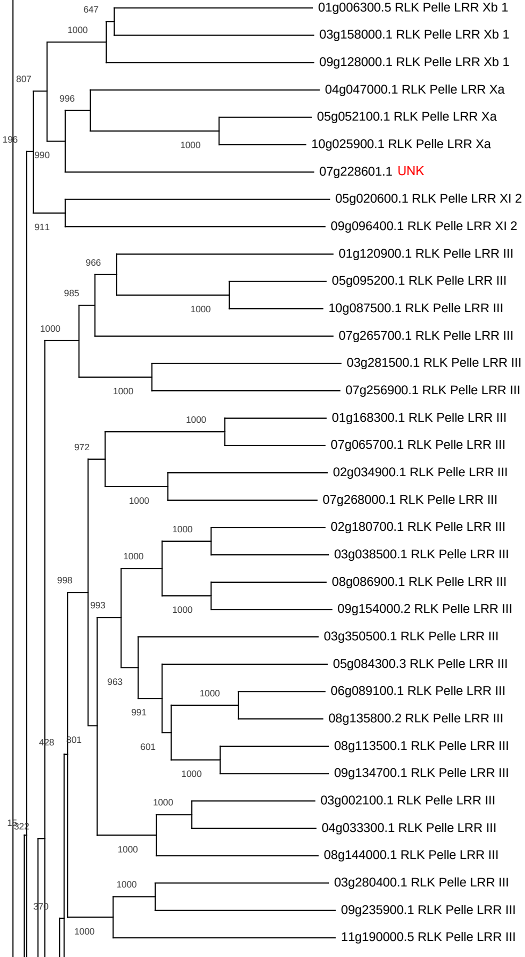
01g006300.5 RLK Pelle LRR Xb 1
03g158000.1 RLK Pelle LRR Xb 1
09g128000.1 RLK Pelle LRR Xb 1
04g047000.1 RLK Pelle LRR Xa
05g052100.1 RLK Pelle LRR Xa
10g025900.1 RLK Pelle LRR Xa
07g228601.1 UNK
05g020600.1 RLK Pelle LRR XI 2
09g096400.1 RLK Pelle LRR XI 2
01g120900.1 RLK Pelle LRR III
05g095200.1 RLK Pelle LRR III
10g087500.1 RLK Pelle LRR III
07g265700.1 RLK Pelle LRR III
03g281500.1 RLK Pelle LRR III
07g256900.1 RLK Pelle LRR III
01g168300.1 RLK Pelle LRR III
07g065700.1 RLK Pelle LRR III
02g034900.1 RLK Pelle LRR III
07g268000.1 RLK Pelle LRR III
02g180700.1 RLK Pelle LRR III
03g038500.1 RLK Pelle LRR III
08g086900.1 RLK Pelle LRR III
09g154000.2 RLK Pelle LRR III
03g350500.1 RLK Pelle LRR III
05g084300.3 RLK Pelle LRR III
06g089100.1 RLK Pelle LRR III
08g135800.2 RLK Pelle LRR III
08g113500.1 RLK Pelle LRR III
09g134700.1 RLK Pelle LRR III
03g002100.1 RLK Pelle LRR III
04g033300.1 RLK Pelle LRR III
08g144000.1 RLK Pelle LRR III
03g280400.1 RLK Pelle LRR III
09g235900.1 RLK Pelle LRR III
11g190000.5 RLK Pelle LRR III
647
1000
807
996
196
990
1000
911
966
985
1000
1000
1000
1000
972
1000
1000
1000
998
993
1000
963
1000
991
428
801
601
1000
1000
15
322
1000
1000
370
1000

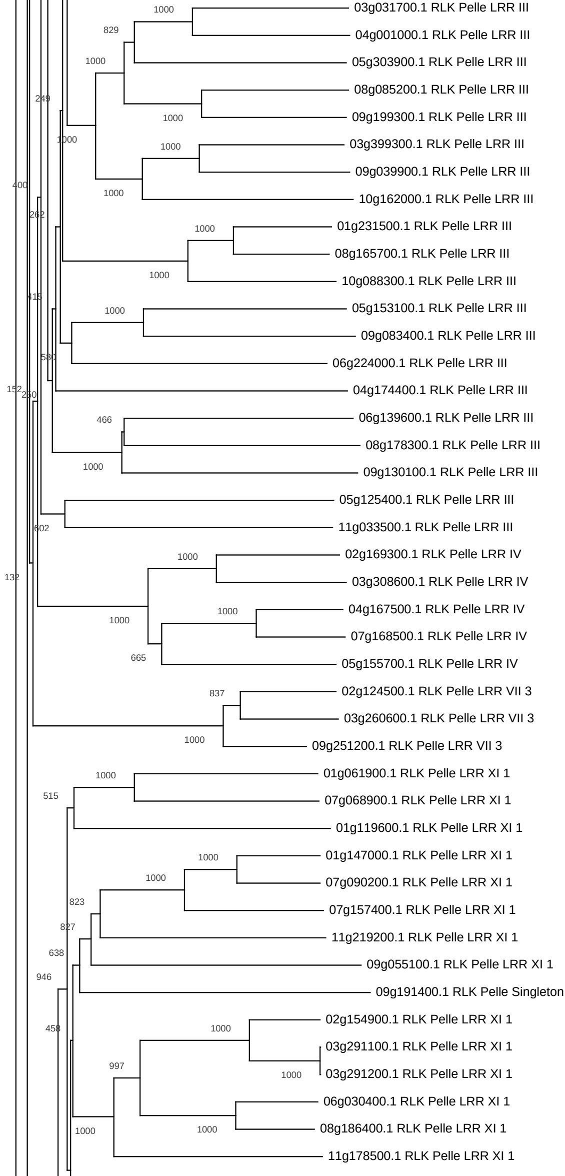
03g031700.1 RLK Pelle LRR III
04g001000.1 RLK Pelle LRR III
05g303900.1 RLK Pelle LRR III
08g085200.1 RLK Pelle LRR III
09g199300.1 RLK Pelle LRR III
03g399300.1 RLK Pelle LRR III
09g039900.1 RLK Pelle LRR III
10g162000.1 RLK Pelle LRR III
01g231500.1 RLK Pelle LRR III
08g165700.1 RLK Pelle LRR III
10g088300.1 RLK Pelle LRR III
05g153100.1 RLK Pelle LRR III
09g083400.1 RLK Pelle LRR III
06g224000.1 RLK Pelle LRR III
04g174400.1 RLK Pelle LRR III
06g139600.1 RLK Pelle LRR III
08g178300.1 RLK Pelle LRR III
09g130100.1 RLK Pelle LRR III
05g125400.1 RLK Pelle LRR III
11g033500.1 RLK Pelle LRR III
02g169300.1 RLK Pelle LRR IV
03g308600.1 RLK Pelle LRR IV
04g167500.1 RLK Pelle LRR IV
07g168500.1 RLK Pelle LRR IV
05g155700.1 RLK Pelle LRR IV
02g124500.1 RLK Pelle LRR VII 3
03g260600.1 RLK Pelle LRR VII 3
09g251200.1 RLK Pelle LRR VII 3
01g061900.1 RLK Pelle LRR XI 1
07g068900.1 RLK Pelle LRR XI 1
01g119600.1 RLK Pelle LRR XI 1
01g147000.1 RLK Pelle LRR XI 1
07g090200.1 RLK Pelle LRR XI 1
07g157400.1 RLK Pelle LRR XI 1
11g219200.1 RLK Pelle LRR XI 1
09g055100.1 RLK Pelle LRR XI 1
09g191400.1 RLK Pelle Singleton
02g154900.1 RLK Pelle LRR XI 1
03g291100.1 RLK Pelle LRR XI 1
03g291200.1 RLK Pelle LRR XI 1
06g030400.1 RLK Pelle LRR XI 1
08g186400.1 RLK Pelle LRR XI 1
11g178500.1 RLK Pelle LRR XI 1

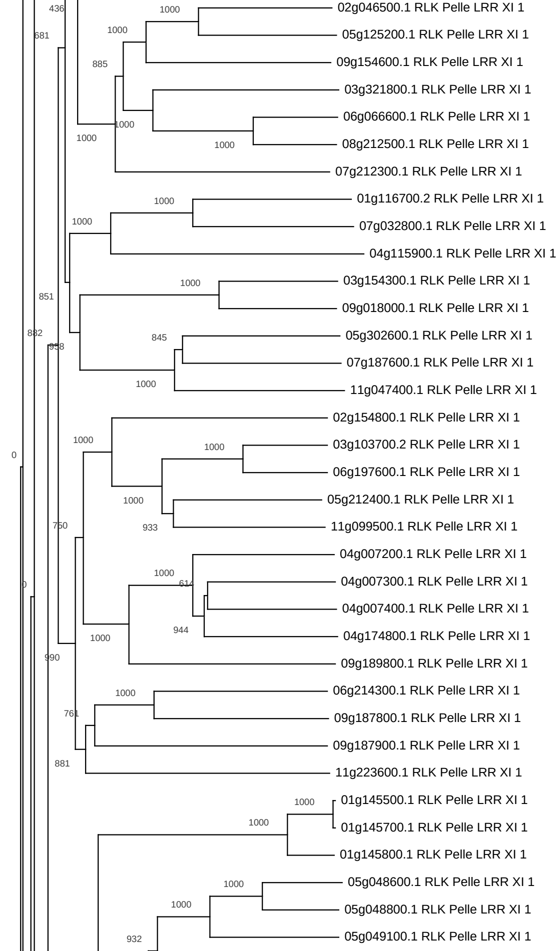
02g046500.1 RLK Pelle LRR XI 1
05g125200.1 RLK Pelle LRR XI 1
09g154600.1 RLK Pelle LRR XI 1
03g321800.1 RLK Pelle LRR XI 1
06g066600.1 RLK Pelle LRR XI 1
08g212500.1 RLK Pelle LRR XI 1
07g212300.1 RLK Pelle LRR XI 1
01g116700.2 RLK Pelle LRR XI 1
07g032800.1 RLK Pelle LRR XI 1
04g115900.1 RLK Pelle LRR XI 1
03g154300.1 RLK Pelle LRR XI 1
09g018000.1 RLK Pelle LRR XI 1
05g302600.1 RLK Pelle LRR XI 1
07g187600.1 RLK Pelle LRR XI 1
11g047400.1 RLK Pelle LRR XI 1
02g154800.1 RLK Pelle LRR XI 1
03g103700.2 RLK Pelle LRR XI 1
06g197600.1 RLK Pelle LRR XI 1
05g212400.1 RLK Pelle LRR XI 1
11g099500.1 RLK Pelle LRR XI 1
04g007200.1 RLK Pelle LRR XI 1
04g007300.1 RLK Pelle LRR XI 1
04g007400.1 RLK Pelle LRR XI 1
04g174800.1 RLK Pelle LRR XI 1
09g189800.1 RLK Pelle LRR XI 1
06g214300.1 RLK Pelle LRR XI 1
09g187800.1 RLK Pelle LRR XI 1
09g187900.1 RLK Pelle LRR XI 1
11g223600.1 RLK Pelle LRR XI 1
01g145500.1 RLK Pelle LRR XI 1
01g145700.1 RLK Pelle LRR XI 1
01g145800.1 RLK Pelle LRR XI 1
05g048600.1 RLK Pelle LRR XI 1
05g048800.1 RLK Pelle LRR XI 1
05g049100.1 RLK Pelle LRR XI 1

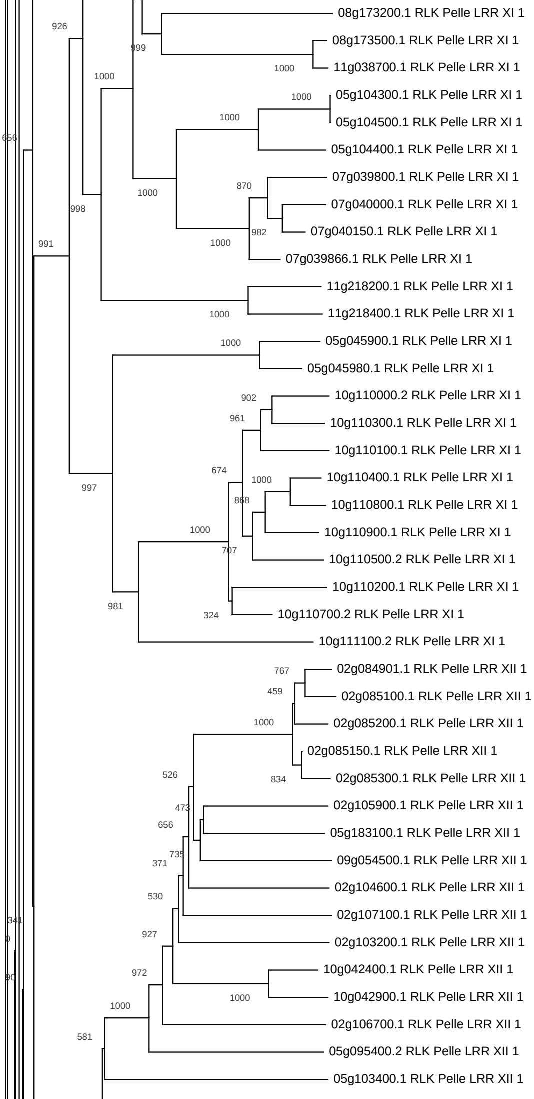

08g173200.1 RLK Pelle LRR XI 1
08g173500.1 RLK Pelle LRR XI 1
11g038700.1 RLK Pelle LRR XI 1
05g104300.1 RLK Pelle LRR XI 1
05g104500.1 RLK Pelle LRR XI 1
05g104400.1 RLK Pelle LRR XI 1
07g039800.1 RLK Pelle LRR XI 1
07g040000.1 RLK Pelle LRR XI 1
07g040150.1 RLK Pelle LRR XI 1
07g039866.1 RLK Pelle LRR XI 1
11g218200.1 RLK Pelle LRR XI 1
11g218400.1 RLK Pelle LRR XI 1
05g045900.1 RLK Pelle LRR XI 1
05g045980.1 RLK Pelle LRR XI 1
10g110000.2 RLK Pelle LRR XI 1
10g110300.1 RLK Pelle LRR XI 1
10g110100.1 RLK Pelle LRR XI 1
10g110400.1 RLK Pelle LRR XI 1
10g110800.1 RLK Pelle LRR XI 1
10g110900.1 RLK Pelle LRR XI 1
10g110500.2 RLK Pelle LRR XI 1
10g110200.1 RLK Pelle LRR XI 1
10g110700.2 RLK Pelle LRR XI 1
10g111100.2 RLK Pelle LRR XI 1
02g084901.1 RLK Pelle LRR XII 1
02g085100.1 RLK Pelle LRR XII 1
02g085200.1 RLK Pelle LRR XII 1
02g085150.1 RLK Pelle LRR XII 1
02g085300.1 RLK Pelle LRR XII 1
02g105900.1 RLK Pelle LRR XII 1
05g183100.1 RLK Pelle LRR XII 1
09g054500.1 RLK Pelle LRR XII 1
02g104600.1 RLK Pelle LRR XII 1
02g107100.1 RLK Pelle LRR XII 1
02g103200.1 RLK Pelle LRR XII 1
10g042400.1 RLK Pelle LRR XII 1
10g042900.1 RLK Pelle LRR XII 1
02g106700.1 RLK Pelle LRR XII 1
05g095400.2 RLK Pelle LRR XII 1
05g103400.1 RLK Pelle LRR XII 1
926
999
1000
1000
1000
1000
656
870
1000
998
982
1000
991
1000
1000
902
961
674
1000
997
868
1000
707
981
324
767
459
1000
526
834
473
656
735
371
530
341
927
972
1000
1000
581

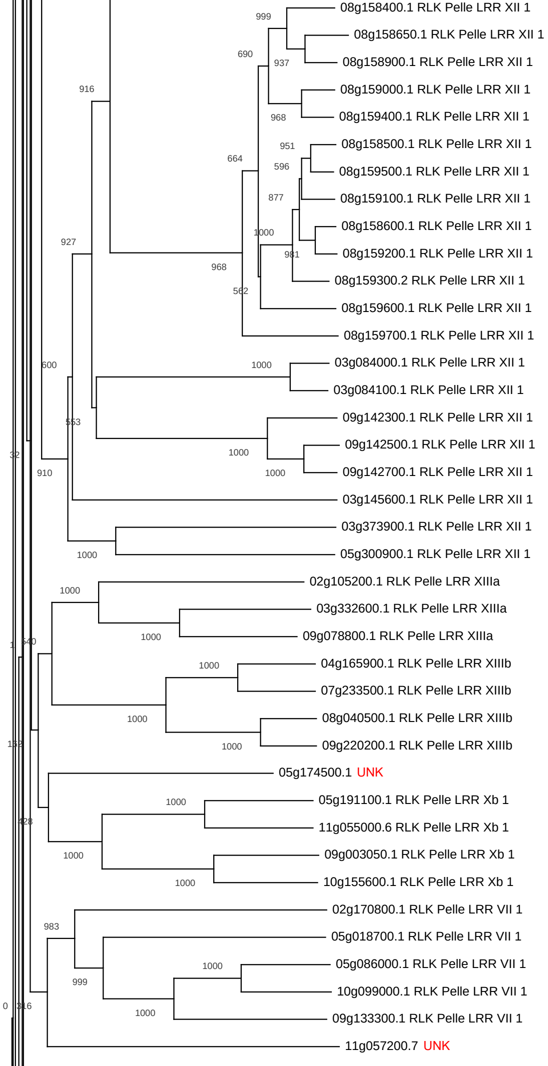
08g158400.1 RLK Pelle LRR XII 1
08g158650.1 RLK Pelle LRR XII 1
08g158900.1 RLK Pelle LRR XII 1
08g159000.1 RLK Pelle LRR XII 1
08g159400.1 RLK Pelle LRR XII 1
08g158500.1 RLK Pelle LRR XII 1
08g159500.1 RLK Pelle LRR XII 1
08g159100.1 RLK Pelle LRR XII 1
08g158600.1 RLK Pelle LRR XII 1
08g159200.1 RLK Pelle LRR XII 1
08g159300.2 RLK Pelle LRR XII 1
08g159600.1 RLK Pelle LRR XII 1
08g159700.1 RLK Pelle LRR XII 1
03g084000.1 RLK Pelle LRR XII 1
03g084100.1 RLK Pelle LRR XII 1
09g142300.1 RLK Pelle LRR XII 1
09g142500.1 RLK Pelle LRR XII 1
09g142700.1 RLK Pelle LRR XII 1
03g145600.1 RLK Pelle LRR XII 1
03g373900.1 RLK Pelle LRR XII 1
05g300900.1 RLK Pelle LRR XII 1
02g105200.1 RLK Pelle LRR XIIIa
03g332600.1 RLK Pelle LRR XIIIa
09g078800.1 RLK Pelle LRR XIIIa
04g165900.1 RLK Pelle LRR XIIIb
07g233500.1 RLK Pelle LRR XIIIb
08g040500.1 RLK Pelle LRR XIIIb
09g220200.1 RLK Pelle LRR XIIIb
05g174500.1 UNK
05g191100.1 RLK Pelle LRR Xb 1
11g055000.6 RLK Pelle LRR Xb 1
09g003050.1 RLK Pelle LRR Xb 1
10g155600.1 RLK Pelle LRR Xb 1
02g170800.1 RLK Pelle LRR VII 1
05g018700.1 RLK Pelle LRR VII 1
05g086000.1 RLK Pelle LRR VII 1
10g099000.1 RLK Pelle LRR VII 1
09g133300.1 RLK Pelle LRR VII 1
11g057200.7 UNK
999
937
690
968
916
951
596
664
877
1000
981
927
968
562
1000
600
553
1000
1000
910
1000
1000
1000
1000
1000
1000
1000
1000
1000
1000
1000
983
999
1000
1000

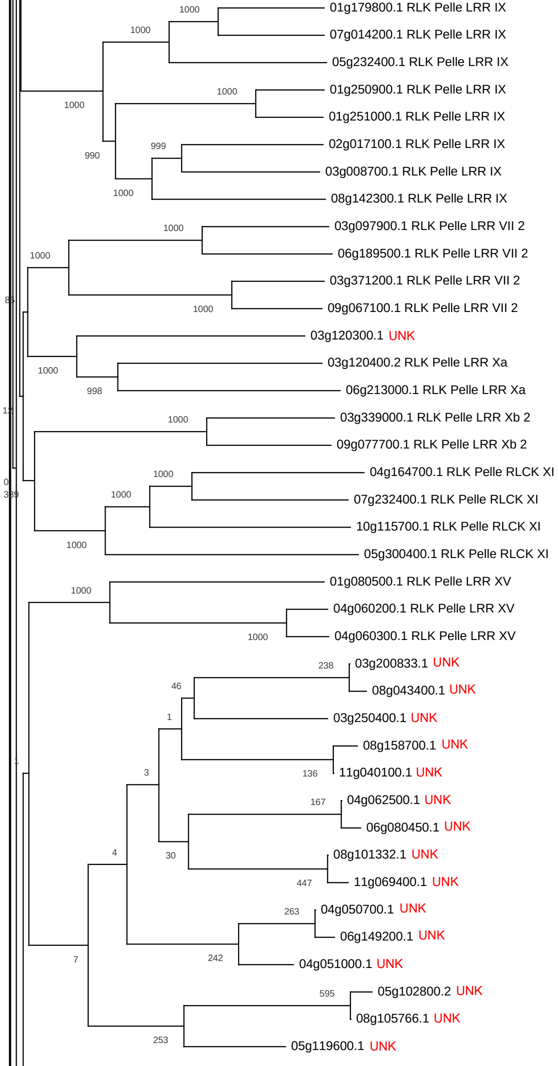

01g179800.1 RLK Pelle LRR IX
07g014200.1 RLK Pelle LRR IX
05g232400.1 RLK Pelle LRR IX
01g250900.1 RLK Pelle LRR IX
01g251000.1 RLK Pelle LRR IX
02g017100.1 RLK Pelle LRR IX
03g008700.1 RLK Pelle LRR IX
08g142300.1 RLK Pelle LRR IX
03g097900.1 RLK Pelle LRR VII 2
06g189500.1 RLK Pelle LRR VII 2
03g371200.1 RLK Pelle LRR VII 2
09g067100.1 RLK Pelle LRR VII 2
03g120300.1 UNK
03g120400.2 RLK Pelle LRR Xa
06g213000.1 RLK Pelle LRR Xa
03g339000.1 RLK Pelle LRR Xb 2
09g077700.1 RLK Pelle LRR Xb 2
04g164700.1 RLK Pelle RLCK XI
07g232400.1 RLK Pelle RLCK XI
10g115700.1 RLK Pelle RLCK XI
05g300400.1 RLK Pelle RLCK XI
01g080500.1 RLK Pelle LRR XV
04g060200.1 RLK Pelle LRR XV
04g060300.1 RLK Pelle LRR XV
03g200833.1 UNK
08g043400.1 UNK
03g250400.1 UNK
08g158700.1 UNK
11g040100.1 UNK
04g062500.1 UNK
06g080450.1 UNK
08g101332.1 UNK
11g069400.1 UNK
04g050700.1 UNK
06g149200.1 UNK
04g051000.1 UNK
05g102800.2 UNK
08g105766.1 UNK
05g119600.1 UNK
1000
1000
1000
1000
990
999
1000
1000
1000
1000
1000
1000
998
1000
1000
1000
1000
1000
1000
1000
238
46
1
136
3
167
30
447
4
263
242
7
595
253

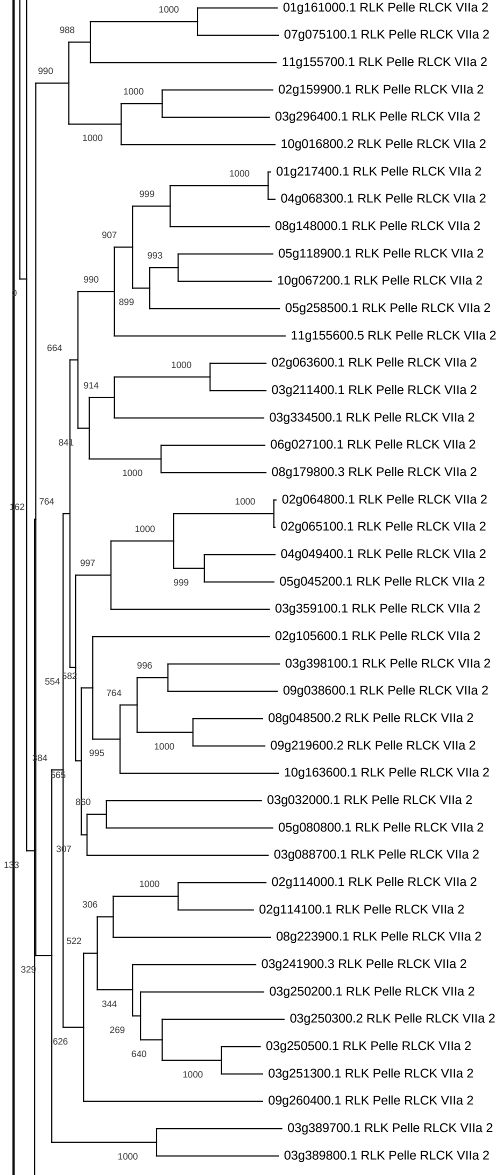
01g161000.1 RLK Pelle RLCK VIIa 2
07g075100.1 RLK Pelle RLCK VIIa 2
11g155700.1 RLK Pelle RLCK VIIa 2
02g159900.1 RLK Pelle RLCK VIIa 2
03g296400.1 RLK Pelle RLCK VIIa 2
10g016800.2 RLK Pelle RLCK VIIa 2
01g217400.1 RLK Pelle RLCK VIIa 2
04g068300.1 RLK Pelle RLCK VIIa 2
08g148000.1 RLK Pelle RLCK VIIa 2
05g118900.1 RLK Pelle RLCK VIIa 2
10g067200.1 RLK Pelle RLCK VIIa 2
05g258500.1 RLK Pelle RLCK VIIa 2
11g155600.5 RLK Pelle RLCK VIIa 2
02g063600.1 RLK Pelle RLCK VIIa 2
03g211400.1 RLK Pelle RLCK VIIa 2
03g334500.1 RLK Pelle RLCK VIIa 2
06g027100.1 RLK Pelle RLCK VIIa 2
08g179800.3 RLK Pelle RLCK VIIa 2
02g064800.1 RLK Pelle RLCK VIIa 2
02g065100.1 RLK Pelle RLCK VIIa 2
04g049400.1 RLK Pelle RLCK VIIa 2
05g045200.1 RLK Pelle RLCK VIIa 2
03g359100.1 RLK Pelle RLCK VIIa 2
02g105600.1 RLK Pelle RLCK VIIa 2
03g398100.1 RLK Pelle RLCK VIIa 2
09g038600.1 RLK Pelle RLCK VIIa 2
08g048500.2 RLK Pelle RLCK VIIa 2
09g219600.2 RLK Pelle RLCK VIIa 2
10g163600.1 RLK Pelle RLCK VIIa 2
03g032000.1 RLK Pelle RLCK VIIa 2
05g080800.1 RLK Pelle RLCK VIIa 2
03g088700.1 RLK Pelle RLCK VIIa 2
02g114000.1 RLK Pelle RLCK VIIa 2
02g114100.1 RLK Pelle RLCK VIIa 2
08g223900.1 RLK Pelle RLCK VIIa 2
03g241900.3 RLK Pelle RLCK VIIa 2
03g250200.1 RLK Pelle RLCK VIIa 2
03g250300.2 RLK Pelle RLCK VIIa 2
03g250500.1 RLK Pelle RLCK VIIa 2
03g251300.1 RLK Pelle RLCK VIIa 2
09g260400.1 RLK Pelle RLCK VIIa 2
03g389700.1 RLK Pelle RLCK VIIa 2
03g389800.1 RLK Pelle RLCK VIIa 2

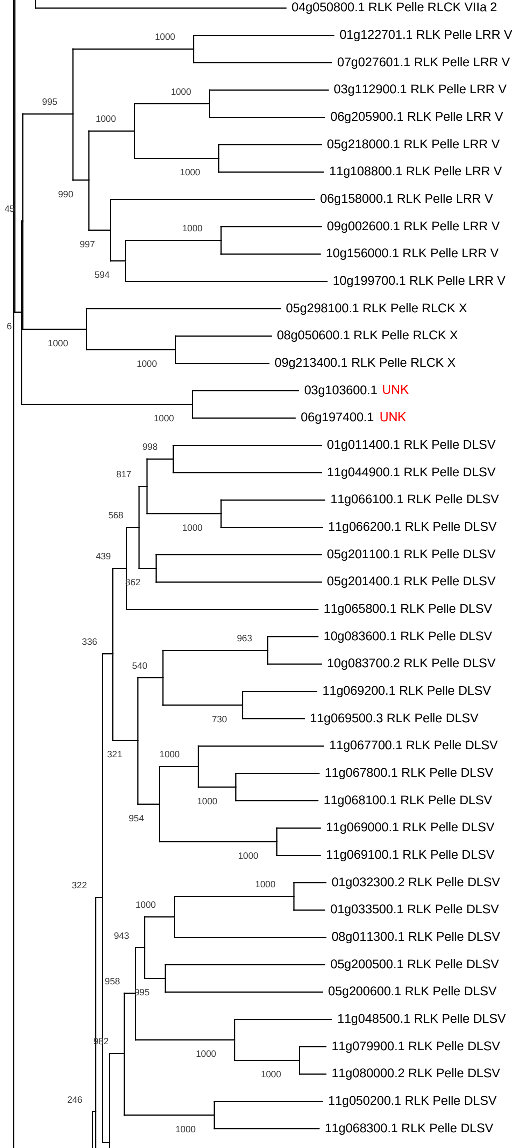
04g050800.1 RLK Pelle RLCK VIIa 2
01g122701.1 RLK Pelle LRR V
07g027601.1 RLK Pelle LRR V
03g112900.1 RLK Pelle LRR V
06g205900.1 RLK Pelle LRR V
05g218000.1 RLK Pelle LRR V
11g108800.1 RLK Pelle LRR V
06g158000.1 RLK Pelle LRR V
09g002600.1 RLK Pelle LRR V
10g156000.1 RLK Pelle LRR V
10g199700.1 RLK Pelle LRR V
05g298100.1 RLK Pelle RLCK X
08g050600.1 RLK Pelle RLCK X
09g213400.1 RLK Pelle RLCK X
03g103600.1 UNK
06g197400.1 UNK
01g011400.1 RLK Pelle DLSV
11g044900.1 RLK Pelle DLSV
11g066100.1 RLK Pelle DLSV
11g066200.1 RLK Pelle DLSV
05g201100.1 RLK Pelle DLSV
05g201400.1 RLK Pelle DLSV
11g065800.1 RLK Pelle DLSV
10g083600.1 RLK Pelle DLSV
10g083700.2 RLK Pelle DLSV
11g069200.1 RLK Pelle DLSV
11g069500.3 RLK Pelle DLSV
11g067700.1 RLK Pelle DLSV
11g067800.1 RLK Pelle DLSV
11g068100.1 RLK Pelle DLSV
11g069000.1 RLK Pelle DLSV
11g069100.1 RLK Pelle DLSV
01g032300.2 RLK Pelle DLSV
01g033500.1 RLK Pelle DLSV
08g011300.1 RLK Pelle DLSV
05g200500.1 RLK Pelle DLSV
05g200600.1 RLK Pelle DLSV
11g048500.1 RLK Pelle DLSV
11g079900.1 RLK Pelle DLSV
11g080000.2 RLK Pelle DLSV
11g050200.1 RLK Pelle DLSV
11g068300.1 RLK Pelle DLSV

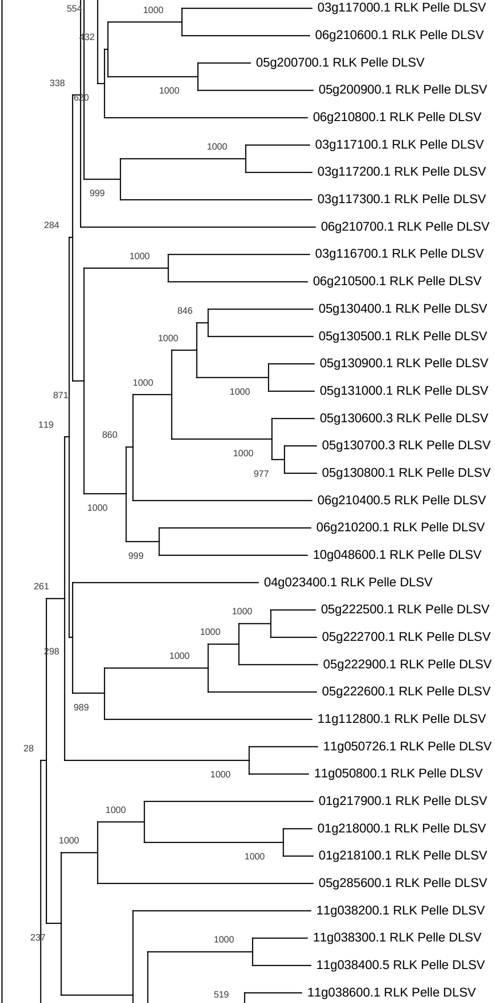
03g117000.1 RLK Pelle DLSV
06g210600.1 RLK Pelle DLSV
05g200700.1 RLK Pelle DLSV
05g200900.1 RLK Pelle DLSV
06g210800.1 RLK Pelle DLSV
03g117100.1 RLK Pelle DLSV
03g117200.1 RLK Pelle DLSV
03g117300.1 RLK Pelle DLSV
06g210700.1 RLK Pelle DLSV
03g116700.1 RLK Pelle DLSV
06g210500.1 RLK Pelle DLSV
05g130400.1 RLK Pelle DLSV
05g130500.1 RLK Pelle DLSV
05g130900.1 RLK Pelle DLSV
05g131000.1 RLK Pelle DLSV
05g130600.3 RLK Pelle DLSV
05g130700.3 RLK Pelle DLSV
05g130800.1 RLK Pelle DLSV
06g210400.5 RLK Pelle DLSV
06g210200.1 RLK Pelle DLSV
10g048600.1 RLK Pelle DLSV
04g023400.1 RLK Pelle DLSV
05g222500.1 RLK Pelle DLSV
05g222700.1 RLK Pelle DLSV
05g222900.1 RLK Pelle DLSV
05g222600.1 RLK Pelle DLSV
11g112800.1 RLK Pelle DLSV
11g050726.1 RLK Pelle DLSV
11g050800.1 RLK Pelle DLSV
01g217900.1 RLK Pelle DLSV
01g218000.1 RLK Pelle DLSV
01g218100.1 RLK Pelle DLSV
05g285600.1 RLK Pelle DLSV
11g038200.1 RLK Pelle DLSV
11g038300.1 RLK Pelle DLSV
11g038400.5 RLK Pelle DLSV
11g038600.1 RLK Pelle DLSV
554
1000
432
338
620
1000
1000
999
284
1000
846
1000
1000
1000
871
119
860
1000
977
1000
999
261
1000
1000
298
1000
989
28
1000
1000
1000
1000
237
1000
519

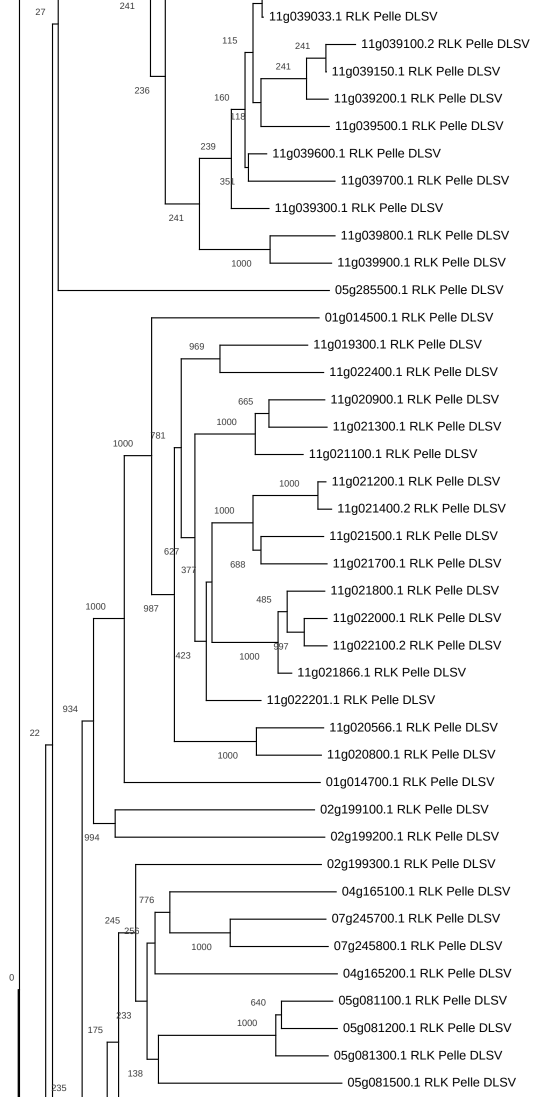
11g039033.1 RLK Pelle DLSV
11g039100.2 RLK Pelle DLSV
11g039150.1 RLK Pelle DLSV
11g039200.1 RLK Pelle DLSV
11g039500.1 RLK Pelle DLSV
11g039600.1 RLK Pelle DLSV
11g039700.1 RLK Pelle DLSV
11g039300.1 RLK Pelle DLSV
11g039800.1 RLK Pelle DLSV
11g039900.1 RLK Pelle DLSV
05g285500.1 RLK Pelle DLSV
01g014500.1 RLK Pelle DLSV
11g019300.1 RLK Pelle DLSV
11g022400.1 RLK Pelle DLSV
11g020900.1 RLK Pelle DLSV
11g021300.1 RLK Pelle DLSV
11g021100.1 RLK Pelle DLSV
11g021200.1 RLK Pelle DLSV
11g021400.2 RLK Pelle DLSV
11g021500.1 RLK Pelle DLSV
11g021700.1 RLK Pelle DLSV
11g021800.1 RLK Pelle DLSV
11g022000.1 RLK Pelle DLSV
11g022100.2 RLK Pelle DLSV
11g021866.1 RLK Pelle DLSV
11g022201.1 RLK Pelle DLSV
11g020566.1 RLK Pelle DLSV
11g020800.1 RLK Pelle DLSV
01g014700.1 RLK Pelle DLSV
02g199100.1 RLK Pelle DLSV
02g199200.1 RLK Pelle DLSV
02g199300.1 RLK Pelle DLSV
04g165100.1 RLK Pelle DLSV
07g245700.1 RLK Pelle DLSV
07g245800.1 RLK Pelle DLSV
04g165200.1 RLK Pelle DLSV
05g081100.1 RLK Pelle DLSV
05g081200.1 RLK Pelle DLSV
05g081300.1 RLK Pelle DLSV
05g081500.1 RLK Pelle DLSV

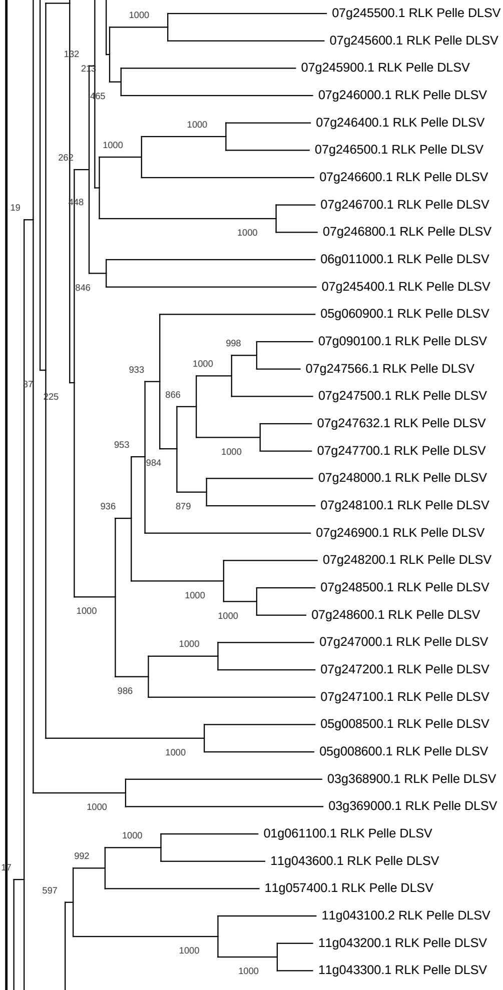
07g245500.1 RLK Pelle DLSV
07g245600.1 RLK Pelle DLSV
07g245900.1 RLK Pelle DLSV
07g246000.1 RLK Pelle DLSV
07g246400.1 RLK Pelle DLSV
07g246500.1 RLK Pelle DLSV
07g246600.1 RLK Pelle DLSV
07g246700.1 RLK Pelle DLSV
07g246800.1 RLK Pelle DLSV
06g011000.1 RLK Pelle DLSV
07g245400.1 RLK Pelle DLSV
05g060900.1 RLK Pelle DLSV
07g090100.1 RLK Pelle DLSV
07g247566.1 RLK Pelle DLSV
07g247500.1 RLK Pelle DLSV
07g247632.1 RLK Pelle DLSV
07g247700.1 RLK Pelle DLSV
07g248000.1 RLK Pelle DLSV
07g248100.1 RLK Pelle DLSV
07g246900.1 RLK Pelle DLSV
07g248200.1 RLK Pelle DLSV
07g248500.1 RLK Pelle DLSV
07g248600.1 RLK Pelle DLSV
07g247000.1 RLK Pelle DLSV
07g247200.1 RLK Pelle DLSV
07g247100.1 RLK Pelle DLSV
05g008500.1 RLK Pelle DLSV
05g008600.1 RLK Pelle DLSV
03g368900.1 RLK Pelle DLSV
03g369000.1 RLK Pelle DLSV
01g061100.1 RLK Pelle DLSV
11g043600.1 RLK Pelle DLSV
11g057400.1 RLK Pelle DLSV
11g043100.2 RLK Pelle DLSV
11g043200.1 RLK Pelle DLSV
11g043300.1 RLK Pelle DLSV

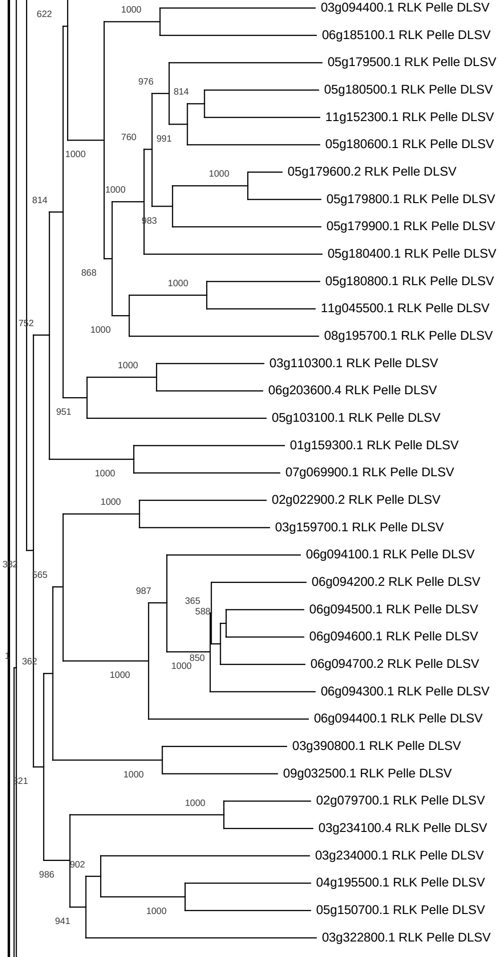
03g094400.1 RLK Pelle DLSV
06g185100.1 RLK Pelle DLSV
05g179500.1 RLK Pelle DLSV
05g180500.1 RLK Pelle DLSV
11g152300.1 RLK Pelle DLSV
05g180600.1 RLK Pelle DLSV
05g179600.2 RLK Pelle DLSV
05g179800.1 RLK Pelle DLSV
05g179900.1 RLK Pelle DLSV
05g180400.1 RLK Pelle DLSV
05g180800.1 RLK Pelle DLSV
11g045500.1 RLK Pelle DLSV
08g195700.1 RLK Pelle DLSV
03g110300.1 RLK Pelle DLSV
06g203600.4 RLK Pelle DLSV
05g103100.1 RLK Pelle DLSV
01g159300.1 RLK Pelle DLSV
07g069900.1 RLK Pelle DLSV
02g022900.2 RLK Pelle DLSV
03g159700.1 RLK Pelle DLSV
06g094100.1 RLK Pelle DLSV
06g094200.2 RLK Pelle DLSV
06g094500.1 RLK Pelle DLSV
06g094600.1 RLK Pelle DLSV
06g094700.2 RLK Pelle DLSV
06g094300.1 RLK Pelle DLSV
06g094400.1 RLK Pelle DLSV
03g390800.1 RLK Pelle DLSV
09g032500.1 RLK Pelle DLSV
02g079700.1 RLK Pelle DLSV
03g234100.4 RLK Pelle DLSV
03g234000.1 RLK Pelle DLSV
04g195500.1 RLK Pelle DLSV
05g150700.1 RLK Pelle DLSV
03g322800.1 RLK Pelle DLSV

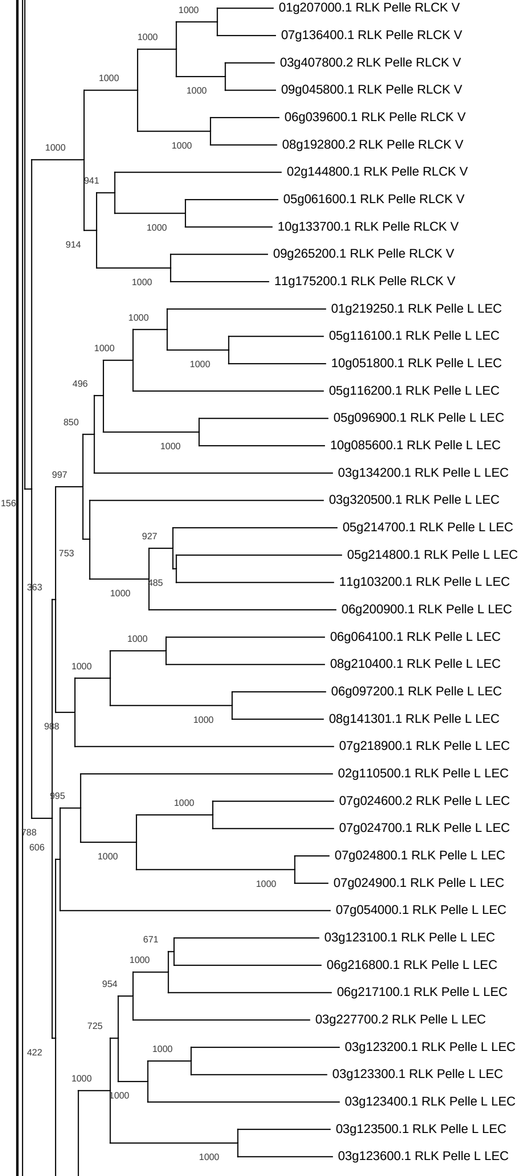
1000
01g207000.1 RLK Pelle RLCK V
1000
07g136400.1 RLK Pelle RLCK V
03g407800.2 RLK Pelle RLCK V
1000
1000
09g045800.1 RLK Pelle RLCK V
06g039600.1 RLK Pelle RLCK V
1000
1000
08g192800.2 RLK Pelle RLCK V
02g144800.1 RLK Pelle RLCK V
941
05g061600.1 RLK Pelle RLCK V
1000
10g133700.1 RLK Pelle RLCK V
914
09g265200.1 RLK Pelle RLCK V
1000
11g175200.1 RLK Pelle RLCK V
01g219250.1 RLK Pelle L LEC
1000
05g116100.1 RLK Pelle L LEC
1000
1000
10g051800.1 RLK Pelle L LEC
496
05g116200.1 RLK Pelle L LEC
850
05g096900.1 RLK Pelle L LEC
1000
10g085600.1 RLK Pelle L LEC
997
03g134200.1 RLK Pelle L LEC
156
03g320500.1 RLK Pelle L LEC
927
05g214700.1 RLK Pelle L LEC
753
05g214800.1 RLK Pelle L LEC
485
11g103200.1 RLK Pelle L LEC
363
1000
06g200900.1 RLK Pelle L LEC
1000
06g064100.1 RLK Pelle L LEC
1000
08g210400.1 RLK Pelle L LEC
06g097200.1 RLK Pelle L LEC
1000
08g141301.1 RLK Pelle L LEC
988
07g218900.1 RLK Pelle L LEC
02g110500.1 RLK Pelle L LEC
995
1000
07g024600.2 RLK Pelle L LEC
07g024700.1 RLK Pelle L LEC
788
606
1000
07g024800.1 RLK Pelle L LEC
1000
07g024900.1 RLK Pelle L LEC
07g054000.1 RLK Pelle L LEC
671
03g123100.1 RLK Pelle L LEC
1000
06g216800.1 RLK Pelle L LEC
954
06g217100.1 RLK Pelle L LEC
725
03g227700.2 RLK Pelle L LEC
1000
03g123200.1 RLK Pelle L LEC
422
03g123300.1 RLK Pelle L LEC
1000
1000
03g123400.1 RLK Pelle L LEC
03g123500.1 RLK Pelle L LEC
1000
03g123600.1 RLK Pelle L LEC

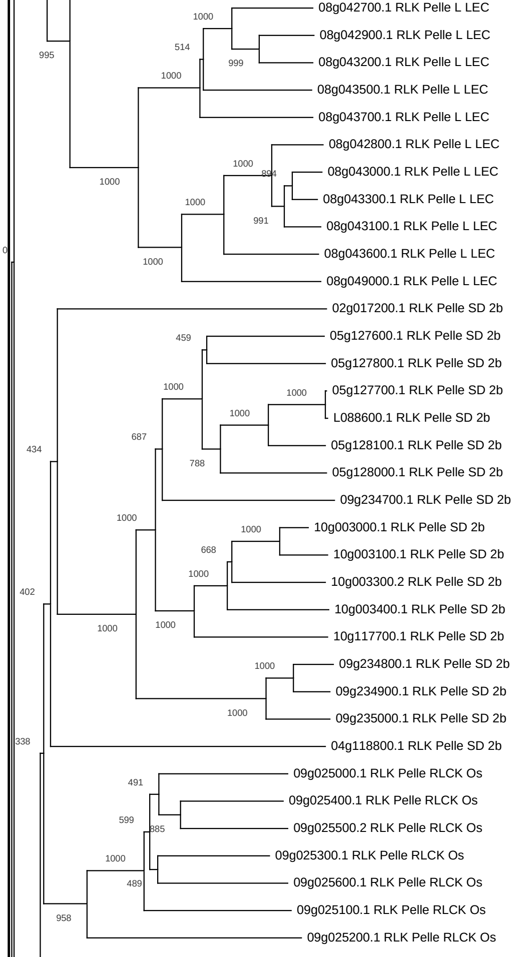
08g042700.1 RLK Pelle L LEC
08g042900.1 RLK Pelle L LEC
08g043200.1 RLK Pelle L LEC
08g043500.1 RLK Pelle L LEC
08g043700.1 RLK Pelle L LEC
08g042800.1 RLK Pelle L LEC
08g043000.1 RLK Pelle L LEC
08g043300.1 RLK Pelle L LEC
08g043100.1 RLK Pelle L LEC
08g043600.1 RLK Pelle L LEC
08g049000.1 RLK Pelle L LEC
02g017200.1 RLK Pelle SD 2b
05g127600.1 RLK Pelle SD 2b
05g127800.1 RLK Pelle SD 2b
05g127700.1 RLK Pelle SD 2b
L088600.1 RLK Pelle SD 2b
05g128100.1 RLK Pelle SD 2b
05g128000.1 RLK Pelle SD 2b
09g234700.1 RLK Pelle SD 2b
10g003000.1 RLK Pelle SD 2b
10g003100.1 RLK Pelle SD 2b
10g003300.2 RLK Pelle SD 2b
10g003400.1 RLK Pelle SD 2b
10g117700.1 RLK Pelle SD 2b
09g234800.1 RLK Pelle SD 2b
09g234900.1 RLK Pelle SD 2b
09g235000.1 RLK Pelle SD 2b
04g118800.1 RLK Pelle SD 2b
09g025000.1 RLK Pelle RLCK Os
09g025400.1 RLK Pelle RLCK Os
09g025500.2 RLK Pelle RLCK Os
09g025300.1 RLK Pelle RLCK Os
09g025600.1 RLK Pelle RLCK Os
09g025100.1 RLK Pelle RLCK Os
09g025200.1 RLK Pelle RLCK Os
1000
514
999
995
1000
1000
1000
894
1000
991
1000
0
459
1000
1000
1000
687
434
788
1000
1000
668
1000
402
1000
1000
1000
1000
338
491
599
885
1000
489
958

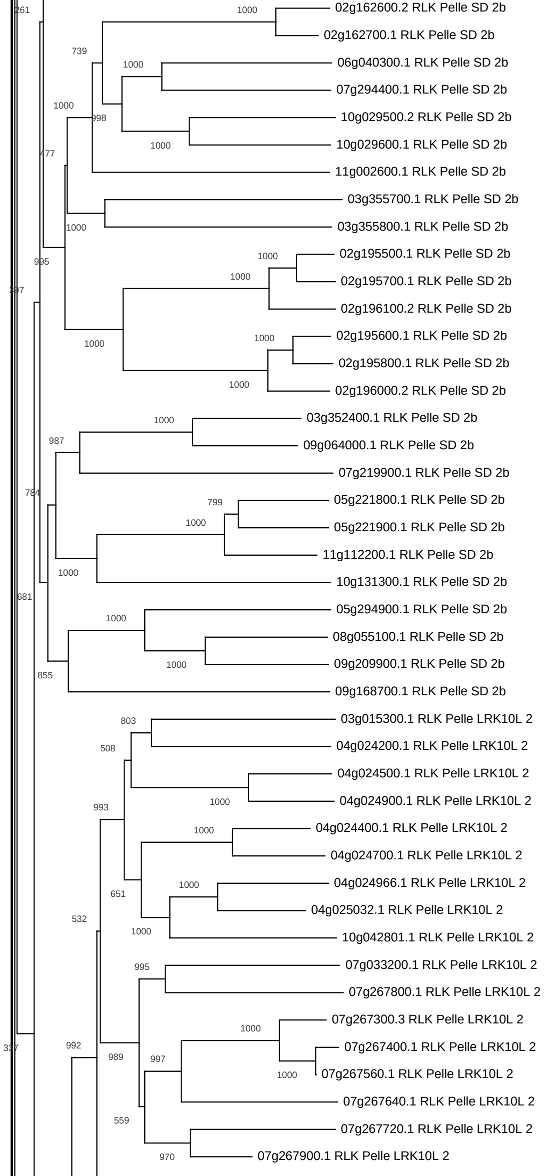
02g162600.2 RLK Pelle SD 2b
02g162700.1 RLK Pelle SD 2b
06g040300.1 RLK Pelle SD 2b
07g294400.1 RLK Pelle SD 2b
10g029500.2 RLK Pelle SD 2b
10g029600.1 RLK Pelle SD 2b
11g002600.1 RLK Pelle SD 2b
03g355700.1 RLK Pelle SD 2b
03g355800.1 RLK Pelle SD 2b
02g195500.1 RLK Pelle SD 2b
02g195700.1 RLK Pelle SD 2b
02g196100.2 RLK Pelle SD 2b
02g195600.1 RLK Pelle SD 2b
02g195800.1 RLK Pelle SD 2b
02g196000.2 RLK Pelle SD 2b
03g352400.1 RLK Pelle SD 2b
09g064000.1 RLK Pelle SD 2b
07g219900.1 RLK Pelle SD 2b
05g221800.1 RLK Pelle SD 2b
05g221900.1 RLK Pelle SD 2b
11g112200.1 RLK Pelle SD 2b
10g131300.1 RLK Pelle SD 2b
05g294900.1 RLK Pelle SD 2b
08g055100.1 RLK Pelle SD 2b
09g209900.1 RLK Pelle SD 2b
09g168700.1 RLK Pelle SD 2b
03g015300.1 RLK Pelle LRK10L 2
04g024200.1 RLK Pelle LRK10L 2
04g024500.1 RLK Pelle LRK10L 2
04g024900.1 RLK Pelle LRK10L 2
04g024400.1 RLK Pelle LRK10L 2
04g024700.1 RLK Pelle LRK10L 2
04g024966.1 RLK Pelle LRK10L 2
04g025032.1 RLK Pelle LRK10L 2
10g042801.1 RLK Pelle LRK10L 2
07g033200.1 RLK Pelle LRK10L 2
07g267800.1 RLK Pelle LRK10L 2
07g267300.3 RLK Pelle LRK10L 2
07g267400.1 RLK Pelle LRK10L 2
07g267560.1 RLK Pelle LRK10L 2
07g267640.1 RLK Pelle LRK10L 2
07g267720.1 RLK Pelle LRK10L 2
07g267900.1 RLK Pelle LRK10L 2

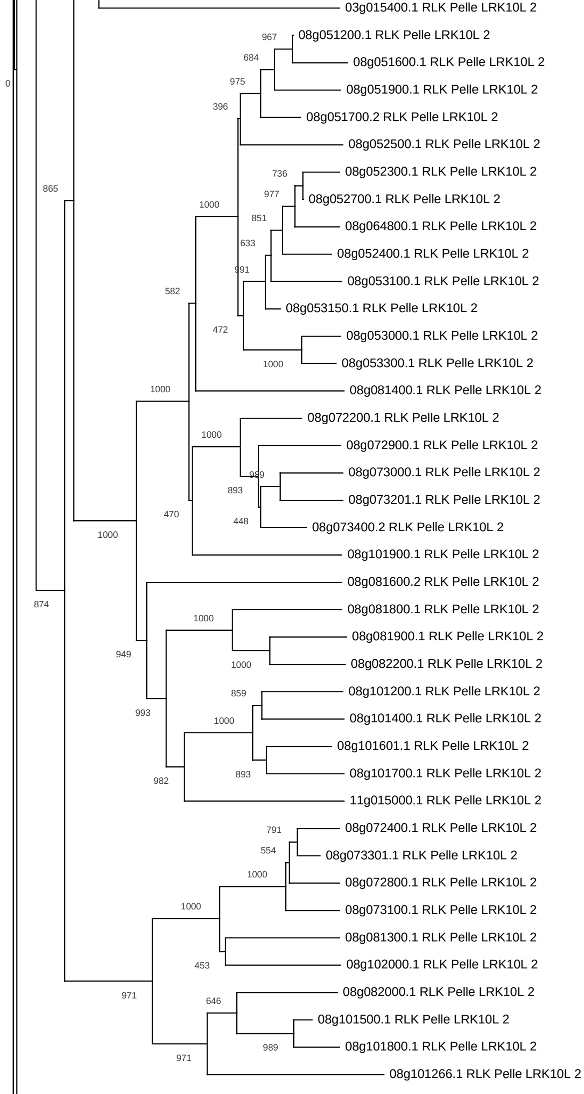

03g015400.1 RLK Pelle LRK10L 2
967
08g051200.1 RLK Pelle LRK10L 2
684
08g051600.1 RLK Pelle LRK10L 2
0
975
08g051900.1 RLK Pelle LRK10L 2
396
08g051700.2 RLK Pelle LRK10L 2
08g052500.1 RLK Pelle LRK10L 2
736
08g052300.1 RLK Pelle LRK10L 2
865
977
08g052700.1 RLK Pelle LRK10L 2
1000
851
08g064800.1 RLK Pelle LRK10L 2
633
08g052400.1 RLK Pelle LRK10L 2
991
08g053100.1 RLK Pelle LRK10L 2
582
08g053150.1 RLK Pelle LRK10L 2
472
08g053000.1 RLK Pelle LRK10L 2
1000
08g053300.1 RLK Pelle LRK10L 2
1000
08g081400.1 RLK Pelle LRK10L 2
08g072200.1 RLK Pelle LRK10L 2
1000
08g072900.1 RLK Pelle LRK10L 2
989
08g073000.1 RLK Pelle LRK10L 2
893
08g073201.1 RLK Pelle LRK10L 2
470
448
08g073400.2 RLK Pelle LRK10L 2
1000
08g101900.1 RLK Pelle LRK10L 2
08g081600.2 RLK Pelle LRK10L 2
874
08g081800.1 RLK Pelle LRK10L 2
1000
08g081900.1 RLK Pelle LRK10L 2
949
1000
08g082200.1 RLK Pelle LRK10L 2
859
08g101200.1 RLK Pelle LRK10L 2
993
08g101400.1 RLK Pelle LRK10L 2
1000
08g101601.1 RLK Pelle LRK10L 2
893
08g101700.1 RLK Pelle LRK10L 2
982
11g015000.1 RLK Pelle LRK10L 2
791
08g072400.1 RLK Pelle LRK10L 2
554
08g073301.1 RLK Pelle LRK10L 2
1000
08g072800.1 RLK Pelle LRK10L 2
1000
08g073100.1 RLK Pelle LRK10L 2
08g081300.1 RLK Pelle LRK10L 2
453
08g102000.1 RLK Pelle LRK10L 2
971
08g082000.1 RLK Pelle LRK10L 2
646
08g101500.1 RLK Pelle LRK10L 2
989
08g101800.1 RLK Pelle LRK10L 2
971
08g101266.1 RLK Pelle LRK10L 2

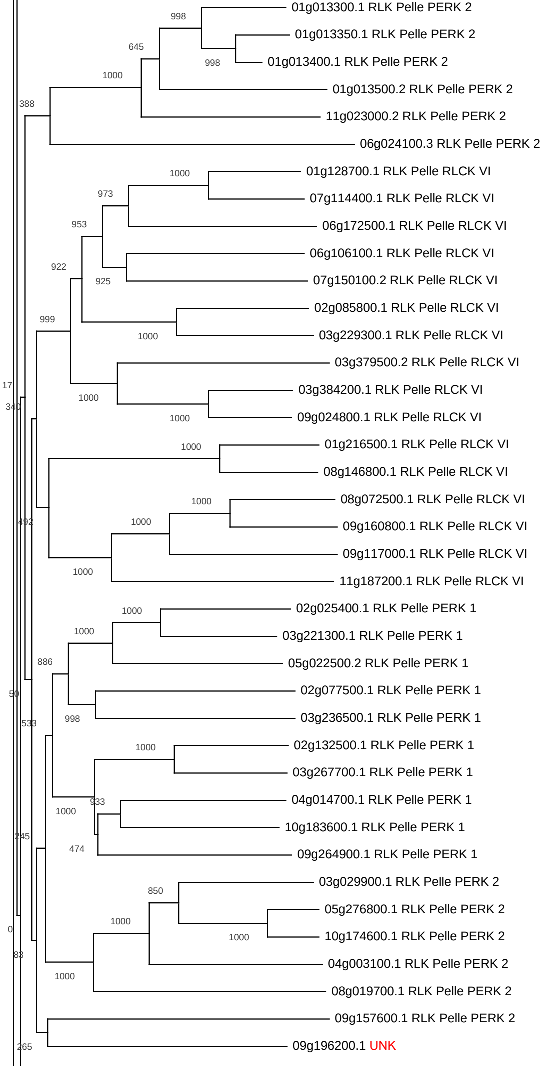
01g013300.1 RLK Pelle PERK 2
01g013350.1 RLK Pelle PERK 2
01g013400.1 RLK Pelle PERK 2
01g013500.2 RLK Pelle PERK 2
11g023000.2 RLK Pelle PERK 2
06g024100.3 RLK Pelle PERK 2
01g128700.1 RLK Pelle RLCK VI
07g114400.1 RLK Pelle RLCK VI
06g172500.1 RLK Pelle RLCK VI
06g106100.1 RLK Pelle RLCK VI
07g150100.2 RLK Pelle RLCK VI
02g085800.1 RLK Pelle RLCK VI
03g229300.1 RLK Pelle RLCK VI
03g379500.2 RLK Pelle RLCK VI
03g384200.1 RLK Pelle RLCK VI
09g024800.1 RLK Pelle RLCK VI
01g216500.1 RLK Pelle RLCK VI
08g146800.1 RLK Pelle RLCK VI
08g072500.1 RLK Pelle RLCK VI
09g160800.1 RLK Pelle RLCK VI
09g117000.1 RLK Pelle RLCK VI
11g187200.1 RLK Pelle RLCK VI
02g025400.1 RLK Pelle PERK 1
03g221300.1 RLK Pelle PERK 1
05g022500.2 RLK Pelle PERK 1
02g077500.1 RLK Pelle PERK 1
03g236500.1 RLK Pelle PERK 1
02g132500.1 RLK Pelle PERK 1
03g267700.1 RLK Pelle PERK 1
04g014700.1 RLK Pelle PERK 1
10g183600.1 RLK Pelle PERK 1
09g264900.1 RLK Pelle PERK 1
03g029900.1 RLK Pelle PERK 2
05g276800.1 RLK Pelle PERK 2
10g174600.1 RLK Pelle PERK 2
04g003100.1 RLK Pelle PERK 2
08g019700.1 RLK Pelle PERK 2
09g157600.1 RLK Pelle PERK 2
09g196200.1 UNK

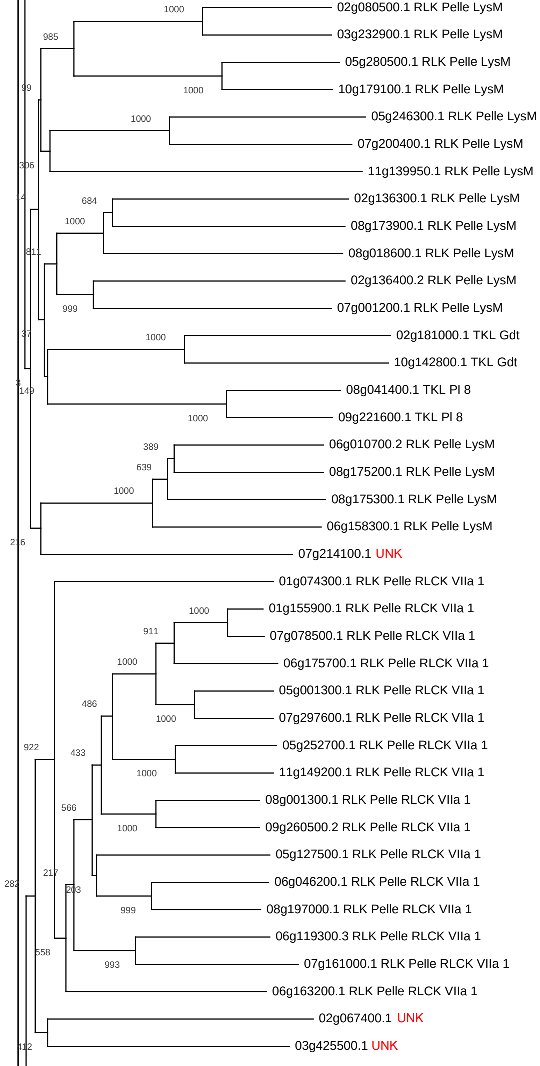

02g080500.1 RLK Pelle LysM
03g232900.1 RLK Pelle LysM
05g280500.1 RLK Pelle LysM
10g179100.1 RLK Pelle LysM
05g246300.1 RLK Pelle LysM
07g200400.1 RLK Pelle LysM
11g139950.1 RLK Pelle LysM
02g136300.1 RLK Pelle LysM
08g173900.1 RLK Pelle LysM
08g018600.1 RLK Pelle LysM
02g136400.2 RLK Pelle LysM
07g001200.1 RLK Pelle LysM
02g181000.1 TKL Gdt
10g142800.1 TKL Gdt
08g041400.1 TKL Pl 8
09g221600.1 TKL Pl 8
06g010700.2 RLK Pelle LysM
08g175200.1 RLK Pelle LysM
08g175300.1 RLK Pelle LysM
06g158300.1 RLK Pelle LysM
07g214100.1 UNK
01g074300.1 RLK Pelle RLCK VIIa 1
01g155900.1 RLK Pelle RLCK VIIa 1
07g078500.1 RLK Pelle RLCK VIIa 1
06g175700.1 RLK Pelle RLCK VIIa 1
05g001300.1 RLK Pelle RLCK VIIa 1
07g297600.1 RLK Pelle RLCK VIIa 1
05g252700.1 RLK Pelle RLCK VIIa 1
11g149200.1 RLK Pelle RLCK VIIa 1
08g001300.1 RLK Pelle RLCK VIIa 1
09g260500.2 RLK Pelle RLCK VIIa 1
05g127500.1 RLK Pelle RLCK VIIa 1
06g046200.1 RLK Pelle RLCK VIIa 1
08g197000.1 RLK Pelle RLCK VIIa 1
06g119300.3 RLK Pelle RLCK VIIa 1
07g161000.1 RLK Pelle RLCK VIIa 1
06g163200.1 RLK Pelle RLCK VIIa 1
02g067400.1 UNK
03g425500.1 UNK

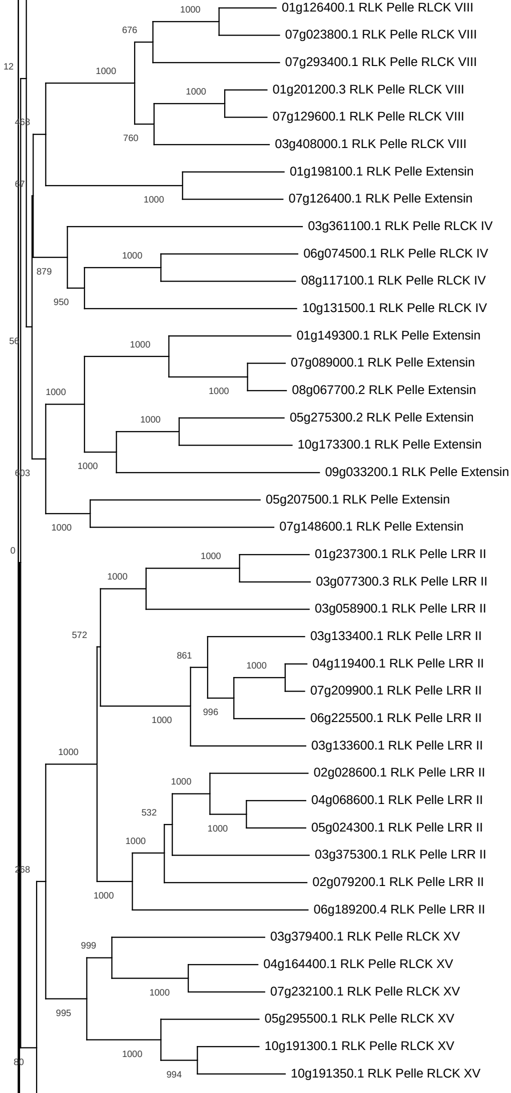

12
1000
01g126400.1 RLK Pelle RLCK VIII
676
07g023800.1 RLK Pelle RLCK VIII
1000
07g293400.1 RLK Pelle RLCK VIII
1000
01g201200.3 RLK Pelle RLCK VIII
07g129600.1 RLK Pelle RLCK VIII
760
03g408000.1 RLK Pelle RLCK VIII
01g198100.1 RLK Pelle Extensin
1000
07g126400.1 RLK Pelle Extensin
03g361100.1 RLK Pelle RLCK IV
1000
06g074500.1 RLK Pelle RLCK IV
879
08g117100.1 RLK Pelle RLCK IV
950
10g131500.1 RLK Pelle RLCK IV
56
1000
01g149300.1 RLK Pelle Extensin
07g089000.1 RLK Pelle Extensin
1000
1000
08g067700.2 RLK Pelle Extensin
1000
05g275300.2 RLK Pelle Extensin
10g173300.1 RLK Pelle Extensin
1000
09g033200.1 RLK Pelle Extensin
05g207500.1 RLK Pelle Extensin
1000
07g148600.1 RLK Pelle Extensin
0
1000
01g237300.1 RLK Pelle LRR II
1000
03g077300.3 RLK Pelle LRR II
03g058900.1 RLK Pelle LRR II
572
03g133400.1 RLK Pelle LRR II
861
1000
04g119400.1 RLK Pelle LRR II
07g209900.1 RLK Pelle LRR II
996
1000
06g225500.1 RLK Pelle LRR II
03g133600.1 RLK Pelle LRR II
1000
02g028600.1 RLK Pelle LRR II
1000
04g068600.1 RLK Pelle LRR II
532
1000
05g024300.1 RLK Pelle LRR II
1000
03g375300.1 RLK Pelle LRR II
02g079200.1 RLK Pelle LRR II
1000
06g189200.4 RLK Pelle LRR II
03g379400.1 RLK Pelle RLCK XV
999
04g164400.1 RLK Pelle RLCK XV
1000
07g232100.1 RLK Pelle RLCK XV
995
05g295500.1 RLK Pelle RLCK XV
10g191300.1 RLK Pelle RLCK XV
1000
994
10g191350.1 RLK Pelle RLCK XV

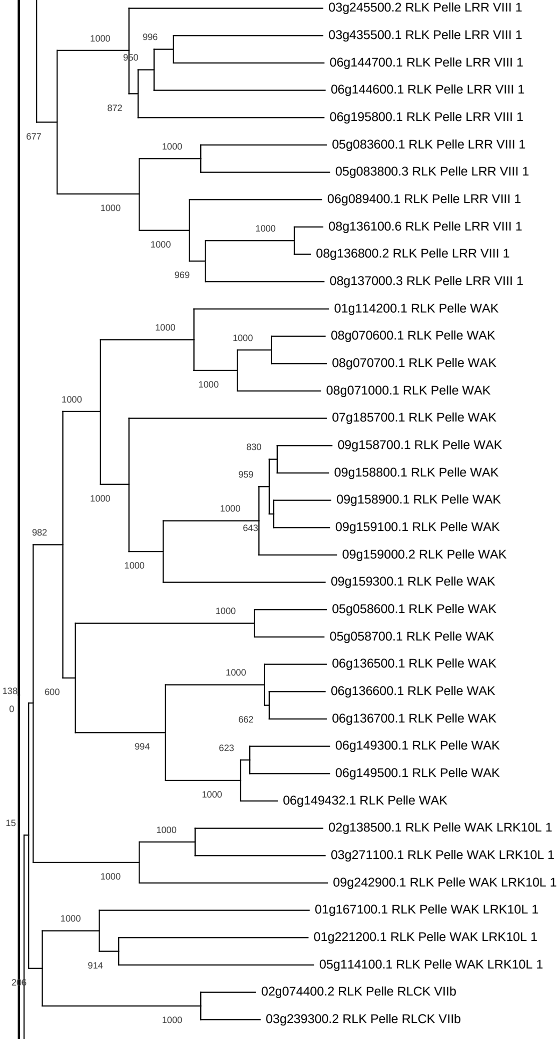
03g245500.2 RLK Pelle LRR VIII 1
03g435500.1 RLK Pelle LRR VIII 1
06g144700.1 RLK Pelle LRR VIII 1
06g144600.1 RLK Pelle LRR VIII 1
06g195800.1 RLK Pelle LRR VIII 1
05g083600.1 RLK Pelle LRR VIII 1
05g083800.3 RLK Pelle LRR VIII 1
06g089400.1 RLK Pelle LRR VIII 1
08g136100.6 RLK Pelle LRR VIII 1
08g136800.2 RLK Pelle LRR VIII 1
08g137000.3 RLK Pelle LRR VIII 1
01g114200.1 RLK Pelle WAK
08g070600.1 RLK Pelle WAK
08g070700.1 RLK Pelle WAK
08g071000.1 RLK Pelle WAK
07g185700.1 RLK Pelle WAK
09g158700.1 RLK Pelle WAK
09g158800.1 RLK Pelle WAK
09g158900.1 RLK Pelle WAK
09g159100.1 RLK Pelle WAK
09g159000.2 RLK Pelle WAK
09g159300.1 RLK Pelle WAK
05g058600.1 RLK Pelle WAK
05g058700.1 RLK Pelle WAK
06g136500.1 RLK Pelle WAK
06g136600.1 RLK Pelle WAK
06g136700.1 RLK Pelle WAK
06g149300.1 RLK Pelle WAK
06g149500.1 RLK Pelle WAK
06g149432.1 RLK Pelle WAK
02g138500.1 RLK Pelle WAK LRK10L 1
03g271100.1 RLK Pelle WAK LRK10L 1
09g242900.1 RLK Pelle WAK LRK10L 1
01g167100.1 RLK Pelle WAK LRK10L 1
01g221200.1 RLK Pelle WAK LRK10L 1
05g114100.1 RLK Pelle WAK LRK10L 1
02g074400.2 RLK Pelle RLCK VIIb
03g239300.2 RLK Pelle RLCK VIIb
1000
996
950
872
677
1000
1000
1000
1000
969
1000
1000
1000
1000
830
959
1000
1000
643
1000
982
1000
1000
138
0
600
662
994
623
1000
15
1000
1000
1000
914
206
1000

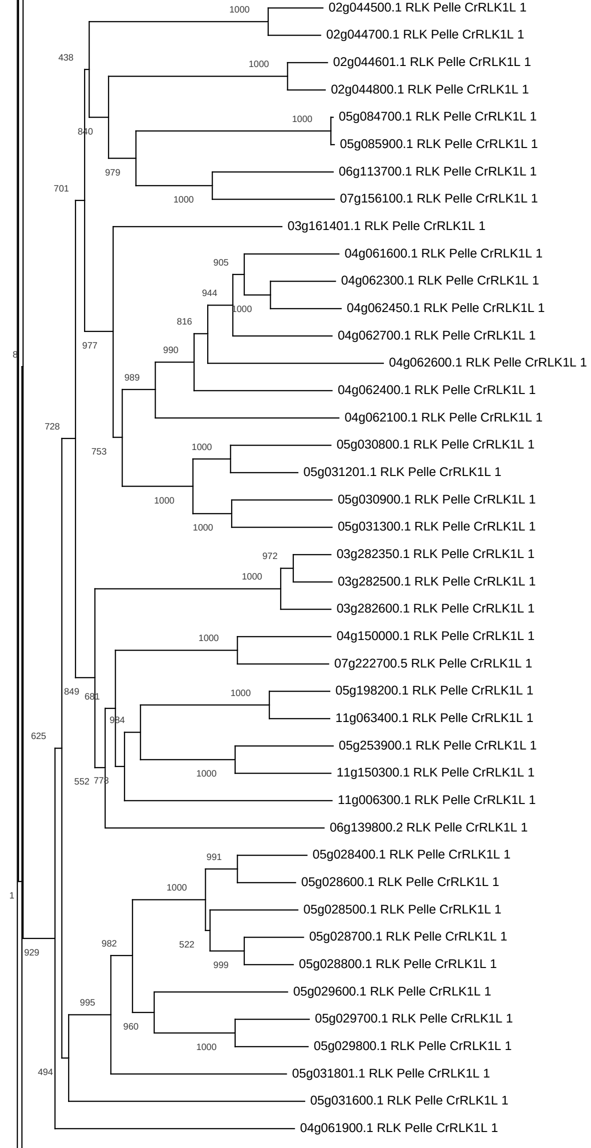

1000
02g044500.1 RLK Pelle CrRLK1L 1
02g044700.1 RLK Pelle CrRLK1L 1
438
1000
02g044601.1 RLK Pelle CrRLK1L 1
02g044800.1 RLK Pelle CrRLK1L 1
1000
05g084700.1 RLK Pelle CrRLK1L 1
840
05g085900.1 RLK Pelle CrRLK1L 1
979
06g113700.1 RLK Pelle CrRLK1L 1
701
1000
07g156100.1 RLK Pelle CrRLK1L 1
03g161401.1 RLK Pelle CrRLK1L 1
905
04g061600.1 RLK Pelle CrRLK1L 1
944
04g062300.1 RLK Pelle CrRLK1L 1
1000
04g062450.1 RLK Pelle CrRLK1L 1
816
04g062700.1 RLK Pelle CrRLK1L 1
977
990
04g062600.1 RLK Pelle CrRLK1L 1
989
04g062400.1 RLK Pelle CrRLK1L 1
04g062100.1 RLK Pelle CrRLK1L 1
728
1000
05g030800.1 RLK Pelle CrRLK1L 1
753
05g031201.1 RLK Pelle CrRLK1L 1
1000
05g030900.1 RLK Pelle CrRLK1L 1
1000
05g031300.1 RLK Pelle CrRLK1L 1
972
03g282350.1 RLK Pelle CrRLK1L 1
1000
03g282500.1 RLK Pelle CrRLK1L 1
03g282600.1 RLK Pelle CrRLK1L 1
1000
04g150000.1 RLK Pelle CrRLK1L 1
07g222700.5 RLK Pelle CrRLK1L 1
849
681
1000
05g198200.1 RLK Pelle CrRLK1L 1
984
11g063400.1 RLK Pelle CrRLK1L 1
625
05g253900.1 RLK Pelle CrRLK1L 1
1000
552
778
11g150300.1 RLK Pelle CrRLK1L 1
11g006300.1 RLK Pelle CrRLK1L 1
06g139800.2 RLK Pelle CrRLK1L 1
991
05g028400.1 RLK Pelle CrRLK1L 1
1000
05g028600.1 RLK Pelle CrRLK1L 1
1
05g028500.1 RLK Pelle CrRLK1L 1
982
522
05g028700.1 RLK Pelle CrRLK1L 1
929
999
05g028800.1 RLK Pelle CrRLK1L 1
05g029600.1 RLK Pelle CrRLK1L 1
995
05g029700.1 RLK Pelle CrRLK1L 1
960
1000
05g029800.1 RLK Pelle CrRLK1L 1
494
05g031801.1 RLK Pelle CrRLK1L 1
05g031600.1 RLK Pelle CrRLK1L 1
04g061900.1 RLK Pelle CrRLK1L 1

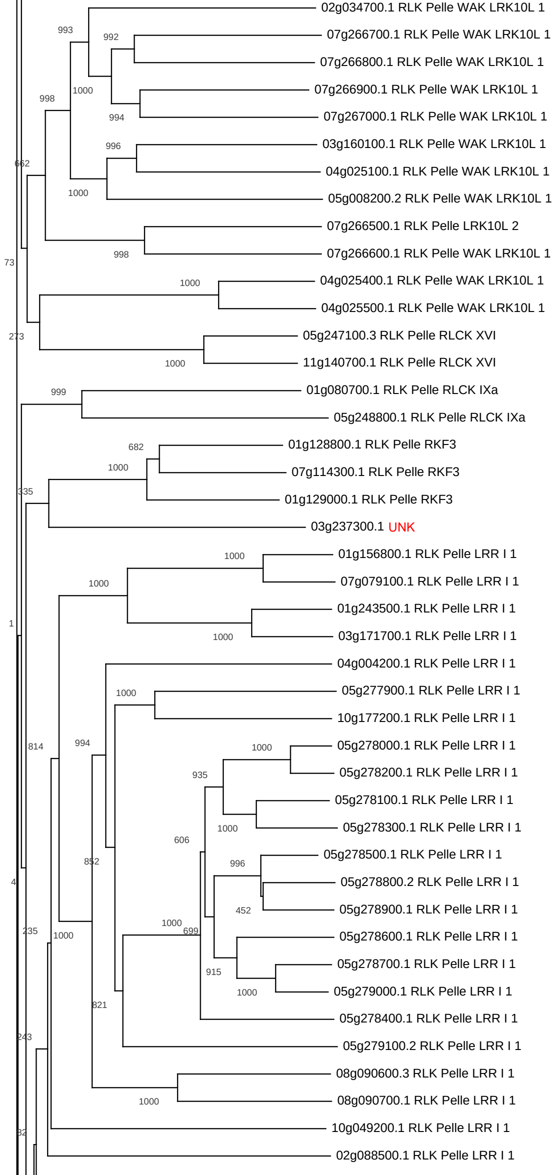
02g034700.1 RLK Pelle WAK LRK10L 1
07g266700.1 RLK Pelle WAK LRK10L 1
07g266800.1 RLK Pelle WAK LRK10L 1
07g266900.1 RLK Pelle WAK LRK10L 1
07g267000.1 RLK Pelle WAK LRK10L 1
03g160100.1 RLK Pelle WAK LRK10L 1
04g025100.1 RLK Pelle WAK LRK10L 1
05g008200.2 RLK Pelle WAK LRK10L 1
07g266500.1 RLK Pelle LRK10L 2
07g266600.1 RLK Pelle WAK LRK10L 1
04g025400.1 RLK Pelle WAK LRK10L 1
04g025500.1 RLK Pelle WAK LRK10L 1
05g247100.3 RLK Pelle RLCK XVI
11g140700.1 RLK Pelle RLCK XVI
01g080700.1 RLK Pelle RLCK IXa
05g248800.1 RLK Pelle RLCK IXa
01g128800.1 RLK Pelle RKF3
07g114300.1 RLK Pelle RKF3
01g129000.1 RLK Pelle RKF3
03g237300.1 UNK
01g156800.1 RLK Pelle LRR I 1
07g079100.1 RLK Pelle LRR I 1
01g243500.1 RLK Pelle LRR I 1
03g171700.1 RLK Pelle LRR I 1
04g004200.1 RLK Pelle LRR I 1
05g277900.1 RLK Pelle LRR I 1
10g177200.1 RLK Pelle LRR I 1
05g278000.1 RLK Pelle LRR I 1
05g278200.1 RLK Pelle LRR I 1
05g278100.1 RLK Pelle LRR I 1
05g278300.1 RLK Pelle LRR I 1
05g278500.1 RLK Pelle LRR I 1
05g278800.2 RLK Pelle LRR I 1
05g278900.1 RLK Pelle LRR I 1
05g278600.1 RLK Pelle LRR I 1
05g278700.1 RLK Pelle LRR I 1
05g279000.1 RLK Pelle LRR I 1
05g278400.1 RLK Pelle LRR I 1
05g279100.2 RLK Pelle LRR I 1
08g090600.3 RLK Pelle LRR I 1
08g090700.1 RLK Pelle LRR I 1
10g049200.1 RLK Pelle LRR I 1
02g088500.1 RLK Pelle LRR I 1

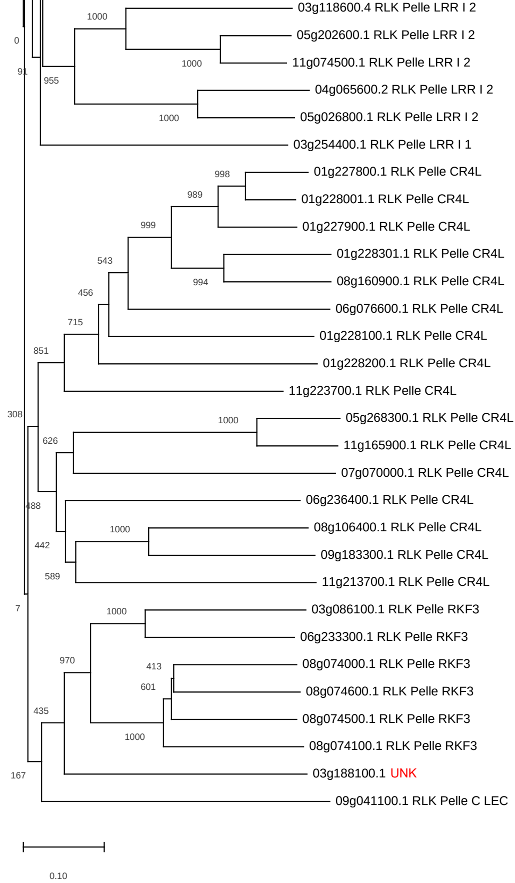
03g118600.4 RLK Pelle LRR I 2
05g202600.1 RLK Pelle LRR I 2
11g074500.1 RLK Pelle LRR I 2
04g065600.2 RLK Pelle LRR I 2
05g026800.1 RLK Pelle LRR I 2
03g254400.1 RLK Pelle LRR I 1
01g227800.1 RLK Pelle CR4L
01g228001.1 RLK Pelle CR4L
01g227900.1 RLK Pelle CR4L
01g228301.1 RLK Pelle CR4L
08g160900.1 RLK Pelle CR4L
06g076600.1 RLK Pelle CR4L
01g228100.1 RLK Pelle CR4L
01g228200.1 RLK Pelle CR4L
11g223700.1 RLK Pelle CR4L
05g268300.1 RLK Pelle CR4L
11g165900.1 RLK Pelle CR4L
07g070000.1 RLK Pelle CR4L
06g236400.1 RLK Pelle CR4L
08g106400.1 RLK Pelle CR4L
09g183300.1 RLK Pelle CR4L
11g213700.1 RLK Pelle CR4L
03g086100.1 RLK Pelle RKF3
06g233300.1 RLK Pelle RKF3
08g074000.1 RLK Pelle RKF3
08g074600.1 RLK Pelle RKF3
08g074500.1 RLK Pelle RKF3
08g074100.1 RLK Pelle RKF3
03g188100.1 UNK
09g041100.1 RLK Pelle C LEC
1000
0
1000
91
955
1000
998
989
999
543
994
456
715
851
308
1000
626
488
1000
442
589
7
1000
970
413
601
435
1000
167
0.10
